# Development of PCR primers enabling the design of flexible sticky ends for efficient concatenation of long DNA fragments†

**DOI:** 10.1039/d3cb00212h

**Published:** 2024-02-26

**Authors:** Kohei Nomura, Kaoru Onda, Hirotaka Murase, Fumitaka Hashiya, Yukiteru Ono, Goro Terai, Natsuhisa Oka, Kiyoshi Asai, Daisuke Suzuki, Naho Takahashi, Haruka Hiraoka, Masahito Inagaki, Yasuaki Kimura, Yoshihiro Shimizu, Naoko Abe, Hiroshi Abe

**Affiliations:** a Department of Chemistry, Graduate School of Science, Nagoya University, Furo-cho, Chikusa-ku Nagoya Aichi 464-8602 Japan h-abe@chem.nagoya-u.ac.jp; b Research Center for Materials Science, Nagoya University Furo-cho, Chikusa-ku Nagoya Aichi 464-8602 Japan; c Department of Computational Biology and Medical Sciences, Graduate School of Frontier Sciences, University of Tokyo Kashiwanoha, Kashiwa Chiba 277-8561 Japan; d Department of Chemistry and Biomolecular Science Faculty of Engineering, Gifu University Gifu 501-1193 Japan; e Laboratory for Cell-Free Protein Synthesis, RIKEN Center for Biosystems Dynamics Research Suita Osaka 565-0874 Japan; f CREST, Japan Science and Technology Agency 7 Gobancho Chiyoda-ku Tokyo 102-0076 Japan; g Institute for Glyco-core Research (iGCORE), Nagoya University Furo-cho, Chikusa-ku Nagoya Aichi 464-8601 Japan

## Abstract

We developed chemically modified PCR primers that allow the design of flexible sticky ends by introducing a photo-cleavable group at the phosphate moiety. Nucleic acid derivatives containing *o*-nitrobenzyl photo-cleavable groups with a *tert*-butyl group at the benzyl position were stable during strong base treatment for oligonucleotide synthesis and thermal cycling in PCR reactions. PCR using primers incorporating these nucleic acid derivatives confirmed that chain extension reactions completely stopped at position 1 before and after the site of the photo-cleavable group was introduced. DNA fragments of 2 and 3 kbp, with sticky ends of 50 bases, were successfully concatenated with a high yield of 77%. A plasmid was constructed using this method. Finally, we applied this approach to construct a 48.5 kbp lambda phage DNA, which is difficult to achieve using restriction enzyme-based methods. After 7 days, we were able to confirm the generation of DNA of the desired length. Although the efficiency is yet to be improved, the chemically modified PCR primer offers potential to complement enzymatic methods and serve as a DNA concatenation technique.

## Abstract 2

We report on chemically modified PCR primers that enable the design of flexible sticky ends by introducing a photo-cleavable group at the phosphate moiety. DNA fragments synthesized using these primers can be ligated with higher efficiency.

## Graphical abstract

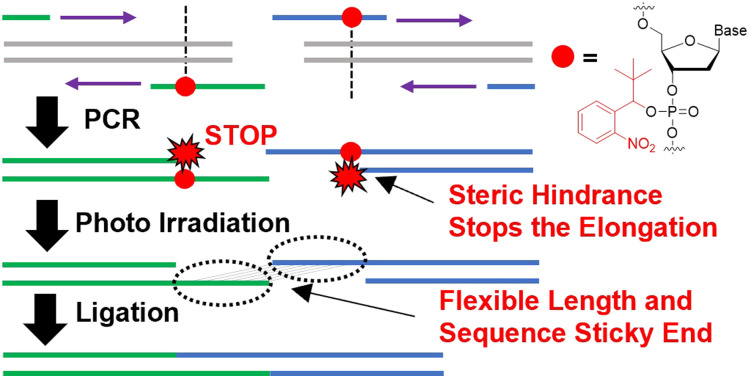

## Introduction

DNA recombination technology is important in the fields of molecular biology, biochemistry, and genetic engineering. The design of proteins with new functions and mRNA sequences to increase translation efficiency requires the creation of highly diverse DNA sequence libraries.^1–4^ The effective linking of DNA fragments to construct DNA libraries is essential for this purpose. In addition, synthetic biology aims to synthesize large genomic DNA, which requires efficient ligation reactions of many DNA fragments.^5–11^ For these applications, the demand for efficient and accurate DNA ligation technology is increasing and is one of the most important issues in the field of genetic engineering.

Different techniques have been developed for linking DNA fragments *in vitro* using enzymes. Restriction endonucleases (REases), which are widely found in prokaryotes, such as bacteria and archaea, have been used to cleave DNA at specific recognition sites.^12–15^ The recognition sequences of REases are often palindromic, and many REases can generate sticky ends that can be used to form hydrogen bonds between the ends of the fragments to be joined to ensure the specificity and efficiency of the subsequent ligation reaction.^16–20^ The disadvantage of using REases to make DNA fragments for ligation is that the recognition sequence of the restriction enzyme is short, approximately 10 bases at the most;^15^ thus, the same sequence can appear with a certain frequency when the target DNA is long. For a typical REase with a 6-base recognition sequence, the same sequence will theoretically occur once every 4096 bases. In addition, the formed sticky ends are too short, often only as long as four bases, to form a stable double strand between the fragments. These drawbacks have been overcome in more recently developed seamless cloning methods. For example, the Gibson Assembly method^21,22^ does not require special recognition sites for enzymes to produce a sticky end. Homologous sequences as short as 15 bases were introduced at both ends of the fragments to be linked. 5′ Exonuclease was applied to the DNA fragments to produce sticky ends with a protruding 3′ end. Single-stranded complementary strands are joined in the system by the action of polymerase and ligase.^21,22^ Gibson and co-workers have achieved the synthesis of a 582 970-base pair *Mycoplasma genitalium* genome from chemically synthesized oligonucleotides, utilizing the assembly method described above.^8^

**Fig. 1:**
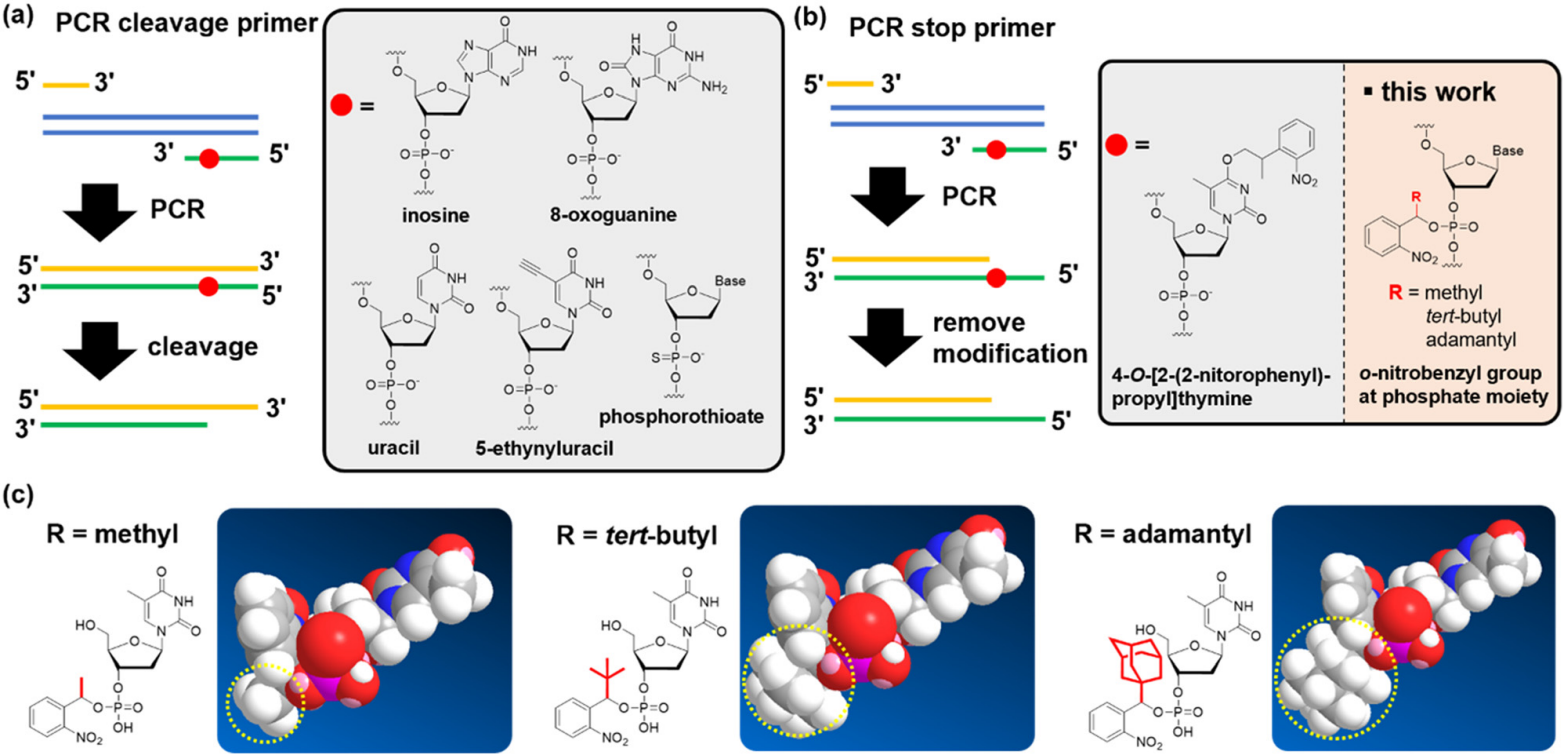
Chemical modifications of a PCR primer to subsequently form a sticky end on the PCR product. Strategies for forming sticky ends can be classified into two types: (a) sticky ends are formed by the cleavage of short DNA strands after the PCR. (b) Sticky ends are formed by stopping chain extension during the PCR. After the PCR, uracil, inosine, 8-oxoguanine, 5-ethynyluracil, and phosphorothioate were used for DNA strand cleavage. 4-*O*-[2-(2-nitrophenyl)-propyl]thymine stopped chain extension during the PCR. In this research, we developed an *o*-nitrobenzyl group at the phosphate part to stop chain extension. (c) We introduced methyl, *tert*-butyl, and adamantyl groups at the benzyl position.

Currently, the polymerase chain reaction (PCR) is extensively used to prepare DNA fragments. PCR primers with various chemical modifications have been developed to produce sticky ends on the dsDNA of the PCR product. These strategies for creating sticky ends with chemical modifications fall into two categories. The first uses a strand-cleavage reaction after the PCR that uses chemically-modified primers, producing a 3′ overhang (Fig. 1(a); defined as a “PCR cleavable primer”). Non-canonical bases, such as uracil,^23–30^ inosine,^31^ 8-oxoguanine,^32^ 5-ethynyluracil,^33^ and phosphorothioate,^34^ are used to induce cleavage (Fig. 1(a)). The position of the modification in the PCR-cleavable primer allows for free adjustment of the length of the sticky ends formed, thus compensating for the disadvantages of using restriction enzymes. However, challenges remain for each modification. In particular, harsh conditions are required to induce the strand cleavage reaction for some modifications, such as strongly basic conditions, elevated temperature, or exposure to oxidative iodine.^33,35^

In the second category, PCR primers that stop chain extension are used to form a sticky end. Specifically, 5′ over-hangs are formed by stopping the chain extension on the opposite strand (Fig. 1(b); defined as the “PCR stop primer”). To date, primers that contain an *o*-nitrobenzyl group, a photo-cleavable protecting group, on the thymidine base have been reported (Fig. 1(b)).^36–39^ By introducing such chemical modifications to the primer, chain extension was stopped because of the inhibition of hydrogen bond formation with the complementary base.^36,37^ One advantage of PCR stop primers for cleavable primers is that they do not require the removal of short-stranded oligodeoxynucleotide fragments. When using PCR-cleavable primers, the cleaved fragment must be quickly dissociated from the complementary strand, but this step is likely to be inefficient if the fragment becomes long. Therefore, if challenges such as the limitation of the relatively low deprotection yield^36^ or the position of the base that accepts the modification can be overcome, we believe that the stopping strategy has the potential to become a promising technology.

In this study, we developed nucleic acid derivatives that contain an *o*-nitrobenzyl modification to the phosphate moiety,^40^ aiming to develop versatile PCR stop primers (Fig. 1(b)). Modifying the phosphate moiety allows the introduction of any base, eliminating restrictions in sequence design. Furthermore, amidite reagents serve as common intermediates, enabling amidite synthesis for all bases, which is highly convenient for manufacturing. We investigated the molecular design of stop primers that can efficiently stop DNA chain extension. Specifically, we designed phosphoramidites with *o*-nitrobenzyl modifications at the phosphate moiety, synthesized various derivatives with substituents at the benzyl position, and examined their chain extension-stopping efficiency. We found that nucleic acid derivatives with an *o*-nitrobenzyl protecting group and a *tert*-butyl group at the benzyl position exhibited high extension-stopping efficiency. Furthermore, we confirmed that this reaction led to efficient ligation of DNA fragments. The use of *o*-nitrobenzyl-modified primers at the phosphate moiety developed in this study makes it possible to synthesize DNA fragments of any length or sequence of sticky ends. We discovered that this method allows for more efficient DNA ligation reactions than the restriction enzyme methods. We demonstrated that this approach can be applied to the ligation of long-chain DNA of more than 10k bases, which is impossible with restriction enzymes.

## Results and discussion

### Design and synthesis of a PCR stop primer

We designed a PCR stop primer with an *o*-nitrobenzyl protecting group that can be photo-cleaved^41^ at the phosphate moiety of the DNA. Herein, we describe the guidelines for designing this protective group. First, the protecting group must be stable at high temperatures before use in PCR. Although the *o*-nitrobenzyl group, with a methyl group at the benzyl position, has been reported to be introduced as a triester to oligonucleotides, it is prone to hydrolysis, causing stability issues.^40^ Second, the protecting group must efficiently and selectively block primer extension by polymerases to generate the desired product with sticky ends. In the past, caging protecting groups were introduced to the base moieties to stop chain extension; however, these protecting groups were designed to inhibit the hydrogen bonding of the bases.^36,37^ In contrast, when a protecting group is introduced into the phosphate moiety, it is expected that the steric bulkiness of the group physically inhibits chain extension. Third, the protecting group needs to be stable under strongly basic conditions, such as treatment with concentrated ammonia water, to remove the protecting group of the base moiety after the solid-phase synthesis of oligonucleotides. However, the *o*-nitrobenzyl triester structure introduced to the phosphate moiety may be unstable under such basic conditions;^40^ and therefore, it is necessary to improve stability by introducing a substituent group into the *o*-nitrobenzyl group. We designed molecules with progressively bulkier substituents at the benzyl position to achieve the above three requirements, as shown in Fig. 1(c). With this design, we aimed to develop a PCR stop primer that combines high temperature stability, efficient and selective stopping of chain extension, and stability under basic conditions.

**Scheme 1:**
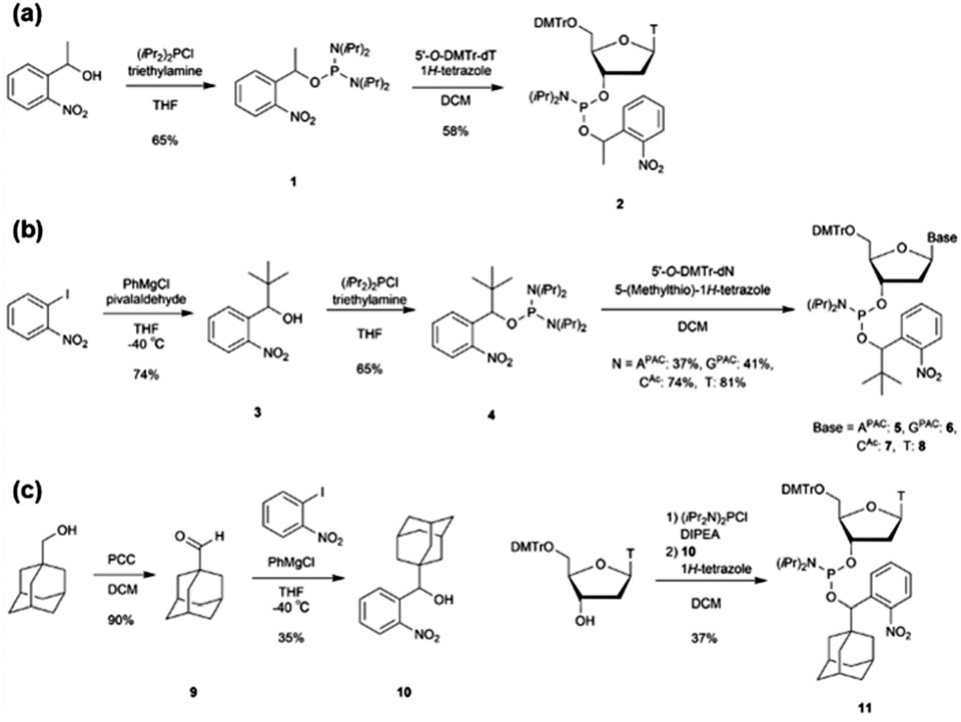
Synthesis of phosphoramidites that contain *o*-nitrobenzyl modification.

We designed *o*-nitrobenzyl protecting groups with methyl, *tert*-butyl, and adamantyl substituents at the benzyl position (R) and synthesized these compounds according to Scheme 1. For R = methyl (Scheme 1(a)) and *tert*-butyl (Scheme 1(b)), we synthesized amidite reagents 1^40^ and 4^42^ by reacting *o*-nitrobenzyl alcohol with an amidite reagent. We then activated the reagents with tetrazole and reacted them with DMTr-protected nucleosides to obtain phosphoramidites 2, 5–8, which contain the *o*-nitrobenzyl group on the phosphorus atom.^40^ For R = adamantyl (Scheme 1(c)), we reacted the DMTr-protected nucleoside with the amidite reagent, and then added *o*-nitrobenzyl alcohol 10^43^ under tetrazole activation to obtain the desired nucleoside phosphoramidite 11.

Using these amidites, we synthesized the DNA strands listed in Table 1 using a DNA synthesizer. Coupling reactions with amidites 2, 5–8, and 11 were confirmed to occur in high yields by extending the reaction time to 15 min. The obtained DNAs were then deprotected with concentrated ammonia water, followed by gel extraction and reverse-phase HPLC purification to obtain the target ODN (Table 1). Reverse-phase high-performance liquid chromatography (HPLC) and mass spectrometry (MS) analyses confirmed the purity of the obtained DNA.

**Table 1:** Sequences and molecular weights of used DNA

Name	Sequence^a^	Calculated	Observed	Used for
ODN_1	5′-ACGACTCAC**T**^**Me**^ATAGGGCGAATTCGAGCTCGGT-3′	9998.58	9998.50	Primer extension assay
ODN_2	5′-ACGACTCA**T**^**Me**^**T**^**Me**^ATAGGGCGAATTCGAGCTCGGT-3′	10 162.74	10 162.80	
ODN_3	5′-ACGACTCAC**T**^***t*Bu**^ATAGGGCGAATTCGAGCTCGGT-3′	10 040.66	10 040.50	
ODN_4	5′-ACGACTCA**T**^***t*Bu**^**T**^***t*Bu**^ATAGGGCGAATTCGAGCTCGGT-3′	10 246.90	10 247.76	
ODN_5	5′-ACGACTC**T**^***t*Bu**^**T**^***t*Bu**^**T**^***t*Bu**^ATAGGGCGAATTCGAGCTCGGT-3′	10 429.11	10 429.00	
ODN_6	5′-ACGACTCAC**T**^**Ad**^ATAGGGCGAATTCGAGCTCGGT-3′	10 118.55	10 118.70	
ODN_7	5′-ACGACTCA**T**^**Ad**^**T**^**Ad**^ATAGGGCGAATTCGAGCTCGGT-3′	10 402.69	10 403.00	
ODN_8	5′-ACGACTCA**A**^***t*Bu**^**A**^***t*Bu**^ATAGGGCGAATTCGAGCTCGGT-3′	10 264.93	10 265.85	
ODN_9	5′-ACGACTCA**A**^***t*Bu**^**G**^***t*Bu**^ATAGGGCGAATTCGAGCTCGGT-3′	10 280.93	10 281.13	
ODN_10	5′-ACGACTCA**A**^***t*Bu**^**C**^***t*Bu**^ATAGGGCGAATTCGAGCTCGGT-3′	10 240.90	10 242.67	
ODN_11	5′-ACGACTCA**A**^***t*Bu**^**T**^***t*Bu**^ATAGGGCGAATTCGAGCTCGGT-3′	10 255.91	10 257.02	
ODN_12	5′-ACGACTCA**G**^***t*Bu**^**A**^***t*Bu**^ATAGGGCGAATTCGAGCTCGGT-3′	10 280.93	10 282.16	
ODN_13	5′-ACGACTCA**G**^***t*Bu**^**G**^***t*Bu**^ATAGGGCGAATTCGAGCTCGGT-3′	10 296.93	10 297.89	
ODN_14	5′-ACGACTCA**G**^***t*Bu**^**C**^***t*Bu**^ATAGGGCGAATTCGAGCTCGGT-3′	10 256.90	10 258.86	
ODN_15	5′-ACGACTCA**G**^***t*Bu**^**T**^***t*Bu**^ATAGGGCGAATTCGAGCTCGGT-3′	10 271.91	10 272.64	
ODN_16	5′-ACGACTCA**C**^***t*Bu**^**A**^***t*Bu**^ATAGGGCGAATTCGAGCTCGGT-3′	10 240.90	10 242.61	
ODN_17	5′-ACGACTCA**C**^***t*Bu**^**G**^***t*Bu**^ATAGGGCGAATTCGAGCTCGGT-3′	10 256.90	10 258.59	
ODN_18	5′-ACGACTCA**C**^***t*Bu**^**C**^***t*Bu**^ATAGGGCGAATTCGAGCTCGGT-3′	10 216.88	10 218.27	
ODN_19	5′-ACGACTCA**C**^***t*Bu**^**T**^***t*Bu**^ATAGGGCGAATTCGAGCTCGGT-3′	10 231.89	10 233.67	
ODN_20	5′-ACGACTCA**T**^***t*Bu**^**A**^***t*Bu**^ATAGGGCGAATTCGAGCTCGGT-3′	10 255.91	10 257.07	
ODN_21	5′-ACGACTCA**T**^***t*Bu**^**G**^***t*Bu**^ATAGGGCGAATTCGAGCTCGGT-3′	10 271.91	10 272.99	
ODN_22	5′-ACGACTCA**T**^***t*Bu**^**C**^***t*Bu**^ATAGGGCGAATTCGAGCTCGGT-3′	10 231.89	10 233.44	
ODN_23	5′-ATAGGGCGAATTCGAGCTCGGT-3′	—	—	
ODN_24	5′-ACGACTCACTATAGGGCGAATTCGAGCTCGGT-3′	—	—	
ODN_25	5′-FAM-ACCGAGCTCGAATTCGCC-3′	—	—	
ODN_26	5′-ACGACTCAC**A**^**b**^ATAGGGCGAATTCGAGCTCGGT-3′	10 093.68	9858.40	
			9901.20	
ODN_27	5′-ACGACTCA**A**^**b**^**A**^**b**^ATAGGGCGAATTCGAGCTCGGT-3′	10 352.95	9882.40	
			9925.40	
			9968.20	
ODN_28	5′-ACGACTCAC**A**^**b,p**^ATAGGGCGAATTCGAGCTCGGT-3′	10 284.91	10 049.60	
			10 092.50	
ODN_29	5′-ACGACTCA**A**^**b,p**^**A**^**b,p**^ATAGGGCGAATTCGAGCTCGGT-3′	10 735.41	10 265.20	
			10 308.10	
			10 351.00	
ODN_30	5′-ACGACTCA**A**^**b,p**^**G**^**b,p**^ATAGGGCGAATTCGAGCTCGGT-3′	10 751.40	10 558.74	
ODN_31	5′-ACGACTCA**G**^**b,p**^**C**^**b,p**^ATAGGGCGAATTCGAGCTCGGT-3′	10 727.38	10 493.11	
ODN_32	5′-ACGACTCA**C**^**b,p**^**G**^**b,p**^ATAGGGCGAATTCGAGCTCGGT-3′	10 727.38	10 493.08	
			10 536.11	
ODN_33	5′-FAM-CAGAATGAGTGAACAACCACGGACC-3′	—	—	Ligation of 2 kbp and 3 kbp fragments (ODN_34, 35 were also used for ligation of 19 kbp fragments)
ODN_34	5′-pATGAAACGCCGAGTTAACGCCATCAAAAATAATTCGCGTCTGG	26 176.27	26 180.15	
	CCTTCCTCTCGAG**T**^***t*Bu**^**T**^***t*Bu**^**T**^***t*Bu**^AGCAACGTGTTAGCAGAGCCAAGC-3′			
ODN_35	5′-pAGGAAGGCCAGACGCGAATTATTTTTGATGGCGTTAACTCGGCGT	27 668.17	27 672.48	
	TTCATCTCGAG**T**^***t*Bu**^**T**^***t*Bu**^**T**^***t*Bu**^GATAGTGCGGGTGTTGAATGATTTCCAG-3′			
ODN_36	5′-AGTGAATGTCTGTTATGAGCGAGGAG-3′	—	—	
ODN_37	5′-pAATTCGCGTCTGGCCTTCCTCTCGAG**T**^***t*Bu**^**T**^***t*Bu**^**T**^***t*Bu**^AGCAACGTGTTAGCAGAGCCAAGC-3′	16 913.17	16 915.08	
ODN_38	5′-pAGGAAGGCCAGACGCGAATTCTCGAG**T**^***t*Bu**^**T**^***t*Bu**^**T**^***t*Bu**^GATAGTGCGGGTGTTGAATGATTTCCAG-3′	18 397.17	18 399.56	
ODN_39	5′-GACCTGGTCTCGTATGAGCAACGTGTTAGCAGAGC-3′	—	—	
ODN_40	5′-ACCTGGTCTCGCATAGATAGTGCGGGTGTTGAATG-3′	—	—	
ODN_41	5′-pACATTAGGCACCCCTGGCTTT**A**^***t*Bu**^**C**^***t*Bu**^**A**^***t*Bu**^CTTTATGCTTCCGGCTCGTATG-3′	14 671.79	14 670.74	Plasmid construction
ODN_42	5′-pACGGGGCTGGCTTATTTATTT**T**^***t*Bu**^**T**^***t*Bu**^**G**^***t*Bu**^ACACCAGACCAACTGGTAATGG-3′	14 848.89	14 848.05	
ODN_43	5′-pATAAGCCAGCCCCGTTGACGG**G**^***t*Bu**^**C**^***t*Bu**^**T**^***t*Bu**^TGTCTGCTCCCGGCATCCGCTTA-3′	14 996.99	14 995.80	
ODN_44	5′-pAGGGGTGCCTAATGTGTGAGC**T**^***t*Bu**^**A**^***t*Bu**^**A**^***t*Bu**^CTCACATTAATTGCGTTGCGCTCA-3′	15 467.29	15 465.52	
ODN_45	5′-TTGCCAGCATGGCCTTTAATGAGC-3′	—	—	Ligation of 19 kbp fragments
ODN_46	5′-GATTCGTTCGCGGTTCCAGATTACC-3′	—	—	
ODN_47	5′-CGCGGGTTTTCGCTATTTATGAAAATTTTCCG-3′	—	—	Ligation of 4 long DNA fragments
ODN_48	5′-pACGGTCATGCCGGTTGCCGCTGTTACCGTGCTGCGATCTTCTGC	26 109.09	26 108.34	
	CATCGACGGACG**T**^***t*Bu**^**C**^***t*Bu**^**C**^***t*Bu**^CACATTGGTGACTTTCACCGTGCG-3′			
ODN_49	5′-pTCGATGGCAGAAGATCGCAGCACGGTAACAGCGGCAACCGGCA	26 369.29	26 366.31	
	TGACCGTGACGCC**T**^***t*Bu**^**G**^***t*Bu**^**C**^***t*Bu**^CAGCACCTCGGTGGTGAAAGGGCA-3′			
ODN_50	5′-pCCAGCGCCGTCAGTGTCGCATTCTTCGGTTGTTTACCCGCAAG	26 032.09	26 034.93	
	CGCGTTAGTCATG**G**^***t*Bu**^**T**^***t*Bu**^**G**^***t*Bu**^GTAGCAAAATCTGGATCATTCCCGA-3′			
ODN_51	5′-pTAACGCGCTTGCGGGTAAACAACCGAAGAATGCGACACTGACG	26 638.49	26 641.54	
	GCGCTGGCAGGGC**T**^***t*Bu**^**T**^***t*Bu**^**T**^***t*Bu**^CCACGGCGAAAAATAAATTACCGTA-3′			
ODN_52	5′-pCGCCGCCGCGAACGTCGCGCAGAGAAACAGGCTCAATGGAAAG	26 032.09	26 034.93	
	CAGCAAATCCCCT**G**^***t*Bu**^**T**^***t*Bu**^**T**^***t*Bu**^GGTTGGGGTAAGCGCAAAACCAG-3′			
ODN_53	5′-pTTTGCTGCTTTCCATTGAGCCTGTTTCTCTGCGCGACGTTCGCGG	26 725.49	26 729.50	
	CGGCGTGTTTG**T**^***t*Bu**^**G**^***t*Bu**^**C**^***t*Bu**^ATCCATCTGGATTCTCCTGTCAGTTA-3′			
ODN_54	5′-CGTAACCTGTCGGATCACCGGAAAG-3′	—	—	

a **X** ^**Me**^ has *o*-nitrobenzyl modification at the phosphate moiety with a methyl group at the benzyl position. **X**^***t*Bu**^ has *o*-nitrobenzyl modification at the phosphate moiety with a *tert*-butyl group at the benzyl position. **X**^**Ad**^ has *o*-nitrobenzyl modification at the phosphate moiety with an adamantyl group at the benzyl position. **X**^**b**^ has *o*-nitrobenzyl modification at the base moiety with a *tert*-butyl group at the benzyl position. **X**^**b,p**^ has *o*-nitrobenzyl modification at both base and phosphate moieties with a *tert*-butyl group at the benzyl position.

**Fig. 2:**
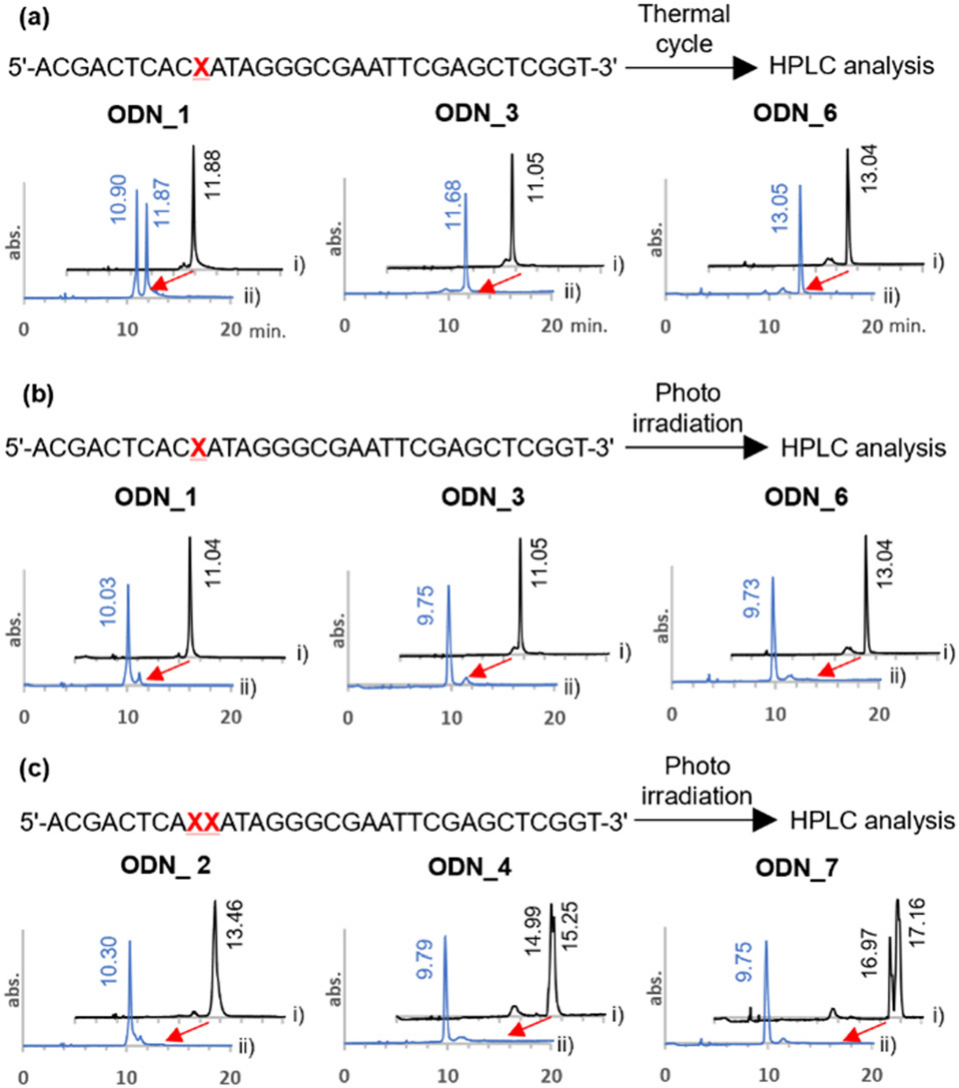
Evaluation of the stability of *o*-nitrobenzyl modified DNA oligo under PCR conditions and the deprotection reaction. (a) HPLC charts of *o*-nitrobenzyl-modified DNA oligos before the thermal cycling (i, black line) and after the thermal cycling (ii, blue line). Thermal cycle was carried out as (95 °C, 1 min → 50 °C, 30 s → 72 °C, 3 min) × 30 cycles. (b), (c) HPLC charts of *o*-nitrobenzyl-modified DNA oligos before photoirradiation (i, black line) and after photoirradiation (ii, blue line). Photoirradiation was performed with 365 nm light at 4 mW cm^−2^ for 10 minutes.

The *o*-nitrobenzyl protecting group of a stop primer must be stable under high temperature conditions of a PCR cycle. Therefore, we synthesized ODN_1, 3, and 6, where phosphoramidites 2, 8, and 11 were introduced at one site in a 32-base DNA strand. We examined the stability of the protecting group by treating ODN_1, 3, and 6 under the general thermal cycle of PCR conditions [(95 °C, 1 min → 50 °C, 30 s → 72 °C, 3 min) × 30 cycles]. After the thermal cycle, the reaction solution was analyzed by reverse-phase HPLC (Fig. 2(a)). As a result, for ODN_1 with a methyl group at R, the product without the *o*-nitrobenzyl group was observed at 47% in the peak area ratio. On the other hand, no deprotection was observed for ODN_3, 6, which introduced a *tert*-butyl group or an adamantyl group at R.

Next, we confirmed the deprotection efficiency by photoirradiation. The DNA solution was irradiated with light at 365 nm at an intensity of 4 mW cm^−2^ for 10 minutes. The deprotection efficiency was examined by analyzing the reaction solution with HPLC (Fig. 2(b)). As a result, quantitative deprotection was confirmed in all cases of ODN_1, 3, and 6. Upon conducting mass analysis of the products after light irradiation using electrospray ionization mass spectrometry (ESI-MS), no products derived from DNA damage were observed. Only the molecular weight of the target was detected (Fig. S1, ESI†). It should be noted that quantitative deprotection was also possible in the cases of ODN_2, 4, and 7, where amidites 2, 8, and 11 were consecutively introduced twice (Fig. 2(c)). The above results demonstrate that this chemical modification exhibits a higher deprotection efficiency compared to the existing 4-*O*-[2-(2-nitrophenyl)-propyl]thymine.^36^

### Effect of substituents at the *o*-nitrobenzyl protecting group for the PCR stop primer

**Fig. 3:**
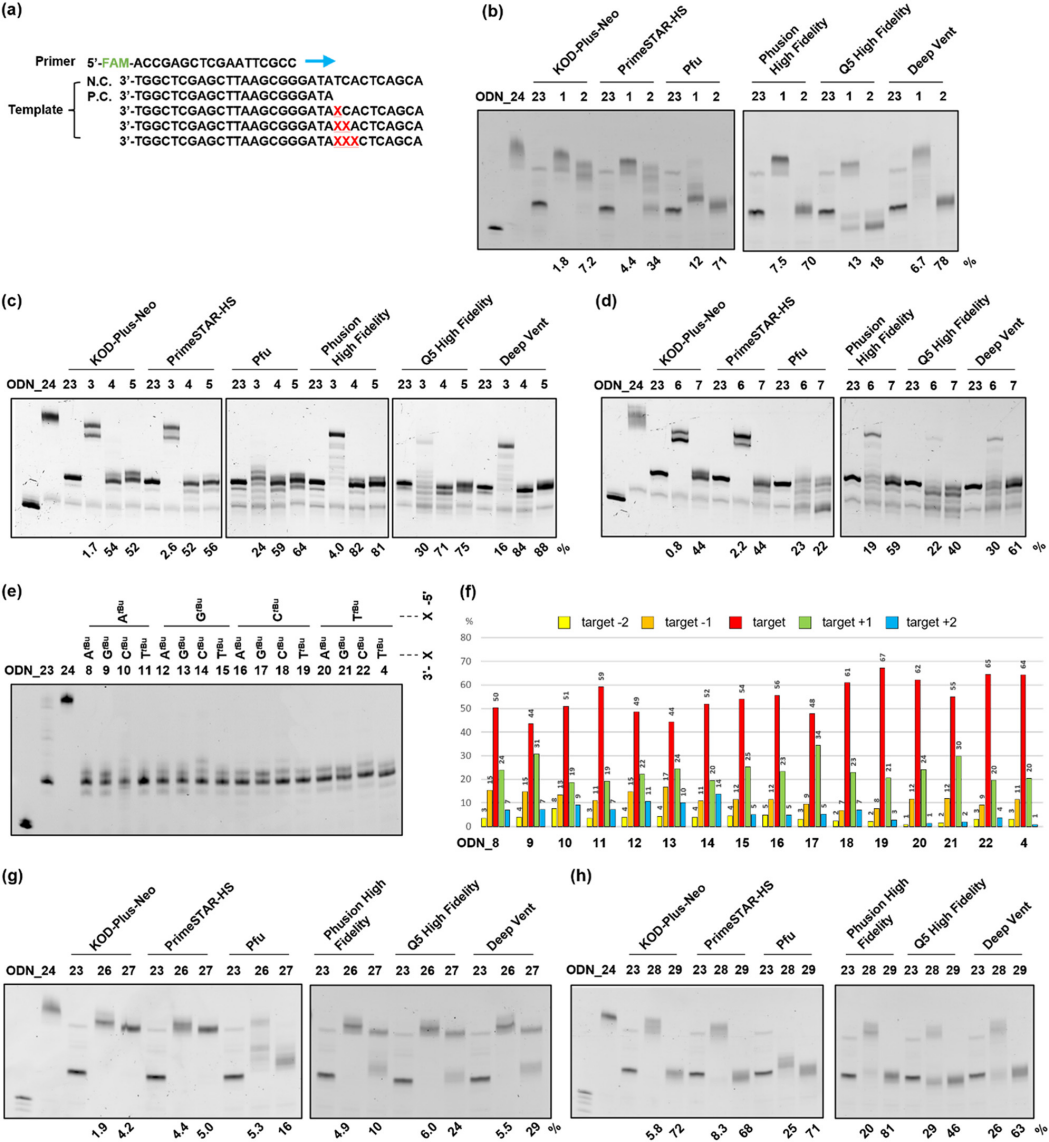
Primer extension assay using *o*-nitrobenzyl-modified DNAs as a template. (a) The sequences used for the primer extension assay. 5′FAM labeled DNA (ODN_25) was used as a primer. ODN_1–24 and 26–29 were used as the template. ODN_24, which has no modification, was used as a negative control. ODN_23, which has a length up to the position of modification, was used as a positive control. Symbol X indicates the position of the modification. (b)–(d), (g), (h) Denaturing PAGE (20%) results of primer extension using the following conditions; 95 °C, 1 min → 50 °C, 30 s → 72 °C, 30 min. (b)–(d) DNAs with the *o*-nitrobenzyl group at the phosphate moiety, which has (b) methyl, (c) *tert*-butyl, and (d) adamantyl group at the benzyl position, were used as templates. (g), (h) DNAs with the *o*-nitrobenzyl group at the (g) base moiety only or (h) both base and phosphate moieties, which have a *tert*-butyl group at the benzyl position, were used. The stopping efficiency was calculated using the ratio of the band intensity of the target length to the positive control. (e) Denaturing PAGE (20%) result when DNAs with two consecutive *o*-nitrobenzyl groups at the phosphate moiety with a *tert*-butyl group at the benzyl position were used. Primer extension was performed under the following PCR conditions; (95 °C for 15 s, 55 °C for 15 s, and 72 °C for 30 s) × 30. (f) The percentages of products at the positions of targets −2, −1, +1, and +2 were calculated as the ratio of the band intensities to the sum of the five band intensities. (b)–(e), (g), (h) The band at the left end of the gel is that of the primer (ODN_25).

Next, to evaluate the chain extension stopping efficiency of various substituents on the *o*-nitrobenzyl protecting group, we conducted a DNA primer extension assay using ODN_1–7, which introduced the *o*-nitrobenzyl protecting group, and ODN_25, which exhibits a fluorescent label at the 5′ end. Different types of DNA polymerases are commercially available with different fidelities and substrate specificities. Therefore, we analyzed the stopping efficiency using six different commercial DNA polymerases, namely, KOD-Plus-Neo (Takara), PrimeSTAR HS (Takara), Pfu DNA polymerase (Promega), Phusion High Fidelity DNA polymerase (New England Biolabs), Q5 High Fidelity DNA polymerase (New England Biolabs), and Deep Vent DNA polymerase (New England Biolabs). We used ODN_23, the target length at which chain extension stopped, as a positive control for the desired DNA product, and ODN_24, which does not have a protecting group, as a negative control for the PCR stop primer (Fig. 3(a)). We analyzed the reaction solutions by denaturing polyacrylamide gel electrophoresis (PAGE) and examined the extended length. The stopping efficiency was calculated as the ratio of the band intensity of the product to that of ODN_23.

In the case of the template DNA with an R = methyl group, ODN_1, which introduced one modification, almost no chain extension stop was observed at the modification site, regardless of the type of polymerase, and the product that extended to the full length was observed. In contrast, ODN_2, which introduced two consecutive modifications, confirmed an efficient stop at the modification site when using Pfu, Phusion High Fidelity, Q5 High Fidelity, and Deep Vent. The product that stopped at the modification position was the highest at 78% with Deep Vent (Fig. 3(b)). The reason some polymerases had low chain elongation termination efficiency is believed to be that the steric hindrance from the methyl group was insufficient to stop polymerase chain elongation.

Next, in the case of template DNA with R = *tert*-butyl group, ODN_3, which introduced one modification, stop products were observed before and after the target position only when using Pfu and Q5 High Fidelity. In contrast, no chain extension stop was observed with other polymerases, and extended to the entire length. In contrast, ODN_4, which introduced two consecutive modifications, gave extension-stopped products with a high efficiency of 52–84% with all six polymerases. To further enhance the extension-stopping ability, we used ODN_5, which introduces three consecutive modifications. No effect significantly exceeding ODN_4 with the two modifications was observed (Fig. 3(c)). These results suggest that in the case of R = *tert*-butyl, introducing two consecutive modifications creates sufficient steric hindrance to stop the polymerase chain elongation reaction.

In template DNA with an R = adamantyl group, ODN_6 and 7, which introduced one or two modifications, showed the same tendency as ODN_3 and 4 with a *tert*-butyl group. ODN_7, which introduced two modifications, had a high extension-stopping ability, but showed a slightly lower stopping efficiency than ODN_4 with a *tert*-butyl group (Fig. 3(d)). Chain elongation termination occurred before the target position when using the adamantyl group. The high steric hindrance of the adamantyl group inhibits the progression of the polymerase before the modification introduction position. Therefore, the chain elongation termination efficiency at the target position decreased with the adamantyl group compared to that with the *tert*-butyl group.

From the above results, the *o*-nitrobenzyl protecting group with R = methyl exhibits high chain extension-stopping efficiency with some polymerases, but is difficult to use as a PCR stop primer because of its low thermal stability. In contrast, the *o*-nitrobenzyl protecting group with R = *tert*-butyl or adamantyl has high thermal strength and extension-stopping ability and can be applied as a stop primer. In subsequent experiments, we decided to use an *o*-nitrobenzyl protecting group with R = *tert*-butyl as the stop primer because of its higher stopping efficiency and ease of synthesis. In terms of DNA polymerases, Pfu and Q5 high fidelity showed a tendency to halt chain elongation at an earlier stage than the other polymerases. This indicates that these polymerases are more susceptible to the influence of *o*-nitrobenzyl modification, suggesting that the area where the polymerase DNA strand binds may be more sterically constrained. Both Phusion High Fidelity and Deep Vent DNA polymerases demonstrated higher chain elongation termination efficiency at the target position. Therefore, we decided to use Phusion High Fidelity, which is known for its high accuracy, for future experiments.

### Sequence dependence of stopping efficiency

The previous experiment showed that two consecutive modifications provided a high chain extension-stopping efficiency. To further extend the sequence application range of the stop PCR primer, we examined the stopping efficiency of various sequences by fixing two modifications. When two modifications are introduced consecutively, 4 × 4 = 16 sequence patterns can be designed. In these 16 cases of ODN_4, 8–22, a primer extension assay was conducted using Phusion High Fidelity. In addition, we performed extension reactions using a thermal cycle that is closer to the actual PCR conditions. After the reaction, the stopping efficiency was investigated by analyzing the extension product using denaturing PAGE (Fig. 3(e)). In this experiment, in all samples, extension stops occurred within ±2 base positions from the modification site. Therefore, the sum of the band intensities from target −2 to target +2 was set to 100%, and the band intensity ratio of each extension product was calculated (Fig. 3(f)). It was confirmed that the extension stop occurred efficiently at 82–98% within ±1 base position from the modification site in all 16 patterns. This result indicates that, regardless of the sequence, the chain extension reaction can be stopped with high efficiency by two consecutive modifications in the phosphate moiety.

### PCR stop primer having a protecting group at the base moiety

If a chain extension stop becomes possible with only one modification, the convenience of primer synthesis and molecular design can be significantly improved. Therefore, nucleoside amidites were designed, in which a protecting group was introduced not only to the phosphate moiety but also to the base moiety. The *o*-nitrobenzyl group with R = *tert*-butyl was converted into a carbamate and introduced into the 6-position amino group of adenine, the 2-position amino group of guanine, the 4-position amino group of cytosine, and the *o*-nitrobenzyl group was introduced to the phosphate moiety to design double-protected nucleoside phosphoramidites 18, 24, and 29. In addition, to evaluate the effect of the protecting groups on the base moiety, nucleoside phosphoramidite 17 was designed, in which only the base moiety was protected with *o*-nitrobenzyl carbamate.

**Scheme 2:**
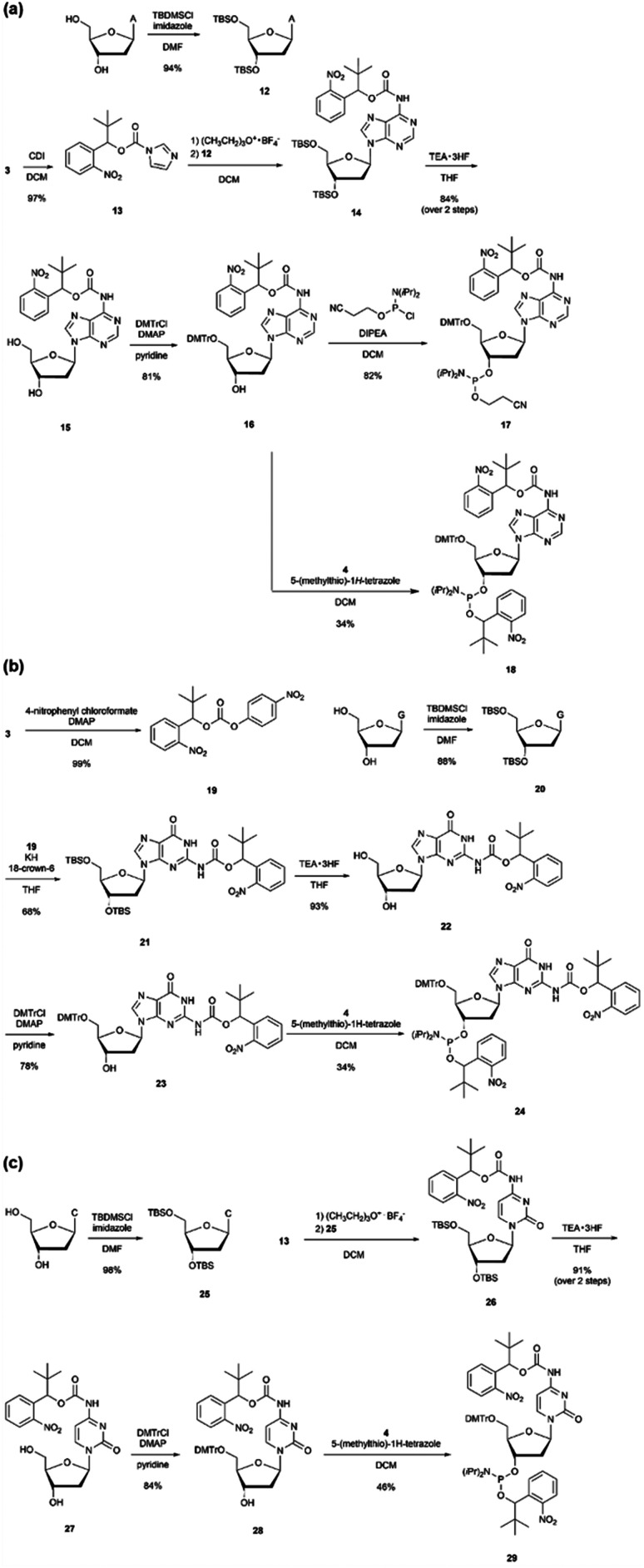
Synthesis of phosphoramidites with *o*-nitrobenzyl modifications at the base and the phosphate moieties.

As shown in Scheme 2(a), the imidazole intermediate 13 was synthesized by reacting *o*-nitrobenzyl alcohol 3 with carbonyldiimidazole. Furthermore, by reacting adenosine 12 with TBS-protected 3′,5′-hydroxyl groups and imidazole intermediate 13, the base protecting group 14 was synthesized.^44^ Compound 16 was obtained *via* the desilylation and tritylation of compound 14. By reacting with commercially available *N*,*N*-diisopropylamino cyanoethyl phosphonamidic-Cl, base-protected adenosine amidite 17, and by reacting with compound 4, adenosine amidite 18 protecting both the base part and phosphate part was synthesized. For guanosine and cytidine, amidites 24 and 29, *o*-nitrobenzyl modification was introduced to both the base and phosphate parts, as shown in Scheme 2(b) and (c). For guanosine, intermediate 19 with *p*-nitrophenol as a leaving group was synthesized by reacting *o*-nitrobenzyl alcohol 3 with 4-nitrophenyl chloroformate. Then, the amino group of guanosine 20 with TBS-protected 3′,5′-hydroxyl groups was deprotonated with potassium hydride, and compound 21 was synthesized by reacting it with intermediate 19.^45^

ODN_26–29, which introduced compound 17, protecting only the base moiety, or compound 18, protecting both the base and phosphate moiety, was synthesized. The number of introduced modifications was set to one or two. The DNA synthesized using a DNA synthesizer was treated with concentrated ammonia water to remove the protecting groups, and the product that lost a certain proportion of the *o*-nitrobenzyl group in the base moiety was confirmed by mass analysis (Fig. S2, ESI†). A primer extension assay was performed using six types of DNA polymerases with the obtained ODN_26–29. The results are presented in Fig. 3(g) and (h). In this experiment, the chain extension-stopping efficiency was calculated as the ratio of the band intensity of the product extended to the modification introduction position to that of the sample using ODN_23. ODN_26 and 27, in which one or two *o*-nitrobenzyl modifications were introduced into only the base moiety, showed almost no chain extension stop in any of the polymerases. This result suggests that the loss of the *o*-nitrobenzyl group from the DNA is one of the causes (Fig. 3(g)).

ODN_28, which introduced compound 18 with a protecting group in both the base and phosphate moieties, gave a slight extension-stopped product of approximately 25–30% when using Pfu, Q5 high fidelity, and Deep Vent. When using Q5 high fidelity, many stopping products shorter than the target position were observed. Furthermore, ODN_29, which introduced two compounds 18, gave all polymerases a high chain extension-stopping efficiency (Fig. 3(h)). However, the chain extension-stopping efficiency was almost the same as that when ODN_4 was used. This result is also believed to be due to the departure of the *o*-nitrobenzyl modification from the base part.

ODN_30–32, which introduced adenosine amidite 18, guanosine amidite 24, and cytidine amidite 29, was also synthesized to protect both the base and phosphate parts with *o*-nitrobenzyl groups. At this time, the protecting group in the base part tends to be partially lost under general deprotection conditions (Fig. S2, ESI†). After thorough analysis, we determined that the most effective molecular design involves a stop PCR primer that introduces a nucleic acid derivative that is only protected by the phosphate group.

### Ligation reaction of DNA fragments using the stop primer

**Fig. 4:**
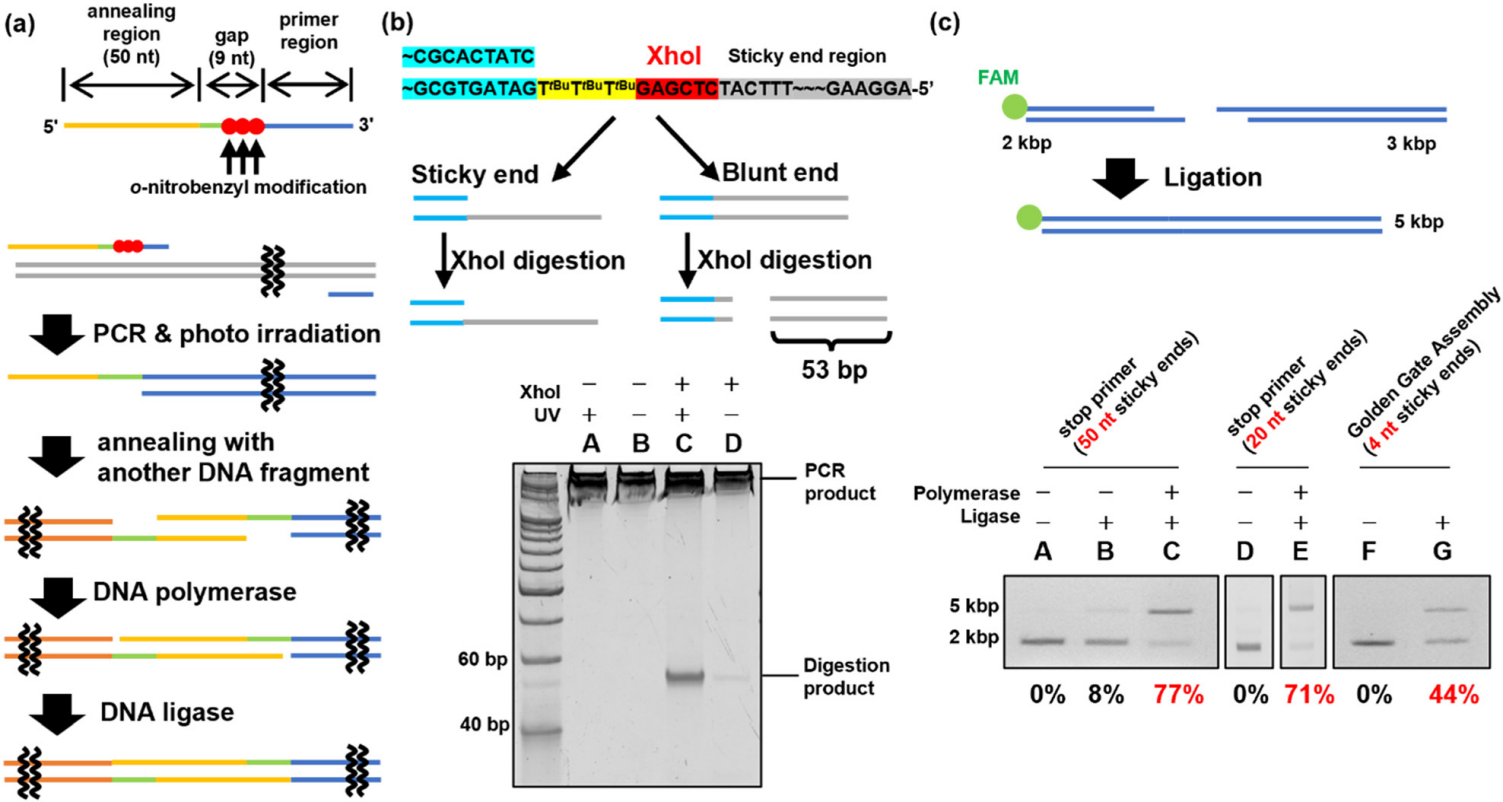
Evaluation of the stop primer. (a) The design of the stop primer and the way of ligation using the stop primer. Three consecutive *o*-nitrobenzyl modifications were introduced at the 5′ side of the primer region. An annealing region was provided after a 9 nucleotide gap. When a PCR using a stop primer and photo-irradiation are performed, natural sticky ends are formed. After the sticky ends are annealed with others, chain extension is performed by DNA polymerase, and ligation is performed by DNA ligase. (b) Confirmation of sticky end formation. XhoI-treated PCR products were synthesized using a stop primer with or without photoirradiation. The sample was analyzed by 16% native PAGE. (c) Ligation using a stop primer or Golden Gate assembly. Lanes (A)–(E): the sample using a stop primer (A)–(C): (50 nt sticky ends, D, E: 20 nt sticky ends). Lanes F and G: the sample using Golden Gate assembly. Lanes A, D, and F: the sample was only hybridized. Lane B: only ligase was added to the sample. Lanes C and E: the samples in which chain extension and ligation were performed. Lane G: the sample in which Golden Gate assembly was performed. All samples were treated with Klenow fragments after hybridization or ligation. The ligation yields were calculated using the band intensity.

In previous experiments, when two *o*-nitrobenzyl modifications with R = *tert*-butyl were introduced consecutively, stopping products were observed at a position ±1 base relative to the modification site. Based on these results, the stop primer is designed as shown in Fig. 4(a) under the following guidelines. 1; to deal with the stopping position either forward or backward from the modification site, a 9-base gap was included when the sticky ends were annealed. After annealing, the DNA polymerase fills the gap and the DNA ligase connects the nick. 2; the formed sticky ends are set to 50 bases, which is difficult to form when using restriction enzymes, to improve the thermodynamic stability in forming a double strand between the sticky ends. 3; three modifications were introduced consecutively to ensure reliability.

PCR was performed using ODN_34 and ODN_35, which introduced modifications with *o*-nitrobenzyl groups in the phosphate moiety to synthesize DNA fragments of 2 and 3 kb, as shown in Fig. 4(c). After amplifying the fragments by PCR, the *o*-nitrobenzyl groups were removed by irradiating with 365 nm light at an intensity of 4 mW cm^−2^ for 15 min, resulting in DNA fragments with sticky ends.

First, sticky ends in the prepared DNA fragments were confirmed using the XhoI restriction enzyme. The sticky ends of the prepared DNA fragments contained an XhoI recognition sequence. Therefore, if sticky ends are formed, the XhoI recognition site remains single-stranded and does not react with XhoI. On the other hand, if chain extension stops do not occur and double-stranded DNA is formed in the annealing region, XhoI digests the double-stranded DNA to produce 53 bp short DNA fragments (Fig. 4(b)). XhoI-treated samples of the 3 kbp DNA fragment were analyzed by native PAGE to confirm the formation of sticky ends by detecting the short DNA fragment. The results are shown in Fig. 4(b). The DNA fragments prepared using the PCR stop primer with the *o*-nitrobenzyl modification pre-removed by photoirradiation produced a short DNA fragment by XhoI digestion, suggesting that no sticky ends were formed (Fig. 4(b), lane C). In contrast, the XhoI digestion product was not observed in the DNA fragments prepared using the PCR stop primer (Fig. 4(b), lane D). Therefore, it was confirmed that the PCR stop primer formed a sticky end in the 3 kb DNA fragment.

DNA fragment ligation was performed to confirm the formation of sticky ends. The 2 kbp and 3 kbp DNA fragments were mixed and annealed to generate the hybridized product. Subsequently, T4 DNA polymerase and T4 DNA ligase were added to the hybridized product to perform gap-filling and nick ligation. The ligation products synthesized by PCR using the stop primer were treated with Klenow Fragment (3′ → 5′ exo-)^46^ before agarose gel electrophoresis. The Klenow fragment is a DNA polymerase with strand displacement activity that can start extension from the nick.^47^ By filling the sticky end region, the non-ligated DNA fragments were re-separated into two fragments, allowing confirmation of ligation. Ligation efficiency was evaluated by the electrophoresis mobility shift assay (EMSA), in which the FAM-labeled 2 kbp DNA fragment was detected. The EMSA of the 2 kbp and 3 kbp DNA fragment ligation indicated that the ligation efficiency was 77% (Fig. 4(c), lane C). For comparison, the Golden Gate Assembly,^16,17^ a commonly used method to construct plasmids, was performed using the same sequence. The EMSA of the Golden Gate Assembly product showed an efficiency of 44% (Fig. 4(c), lane G). Using a PCR stop primer for DNA ligation significantly improved the ligation efficiency compared to the conventional restriction enzyme method. This is believed to be due to the higher thermodynamic stability of the sticky ends formed by the stop primer, with a *T*_m_ value of approximately 76 °C for the 50-base sticky ends, compared to the 4-base sticky ends formed by the restriction enzyme treatment. Additionally, when shorter sticky ends of 20 bases (*T*_m_ value of 64 °C) were synthesized using ODN_37 and ODN_38 as PCR stop primers, a high ligation efficiency of 71% was achieved (Fig. 4(c), lane E). This confirmed that a sticky end length of 20 bases was sufficient, depending on the *T*_m_ value. In addition to the restriction enzyme method used for comparison in this study, there are existing DNA ligation methods such as Gibson assembly^21,22^ and In-Fusion cloning.^48^ However, these techniques are limited to the synthesis of circular DNA. In contrast, DNA ligation using stop primers can be applied to the ligation of linear DNA as well, offering a broader range of applications.

### Plasmid construction using PCR stop primers

**Fig. 5:**
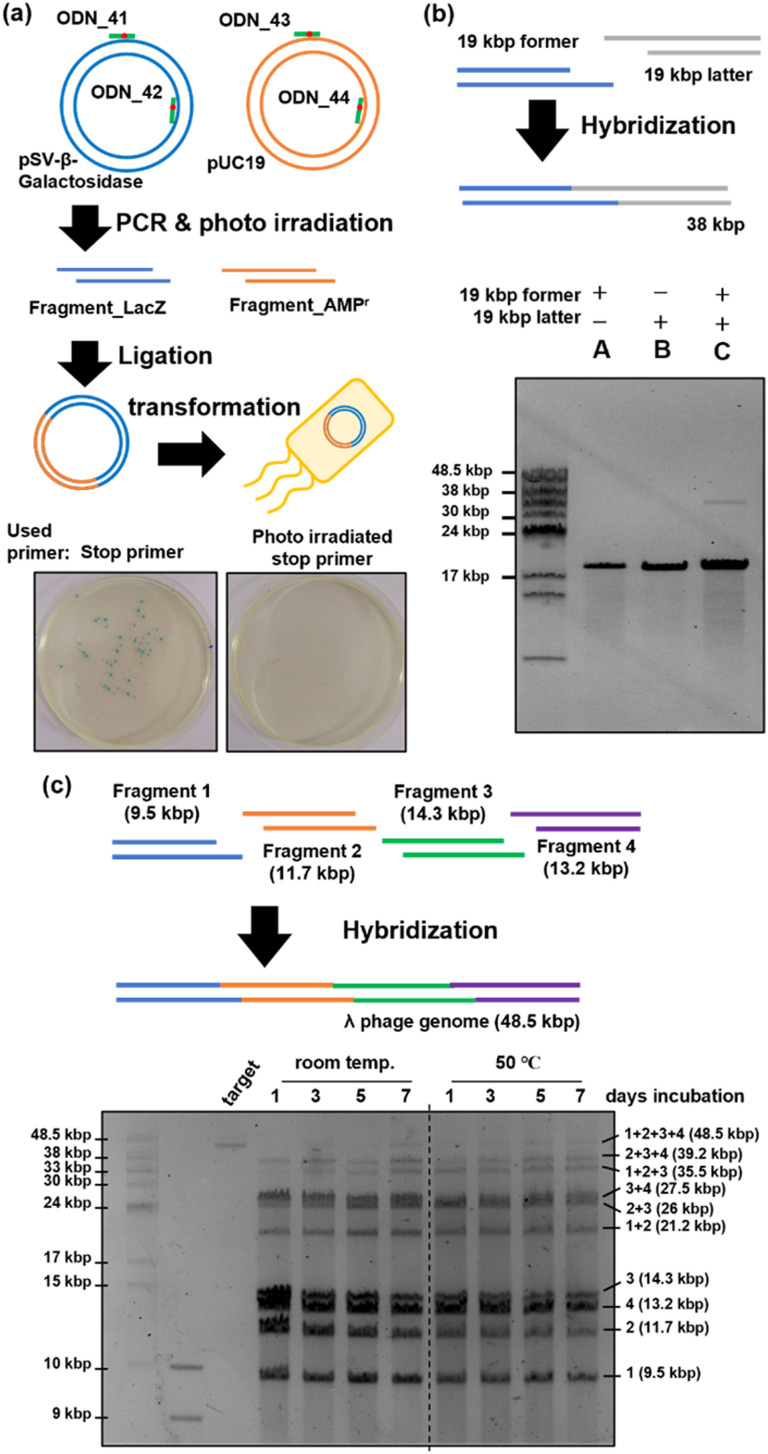
Application of a stop primer. (a) Plasmid construction using a stop primer and confirmation by the blue colony. DNA fragments coding the LacZ gene and ampicillin-resistant gene were synthesized using a photo-irradiated or non-irradiated stop primer. After a ligation reaction like the previous experiment, ligated samples were transformed into competent *E. coli* JM109 cells and cultured on an agar medium containing ampicillin, X-gal, and IPTG. (b) Hybridization of 19 kbp DNA fragments synthesized using a stop primer. (c) Hybridization of 4 long DNA fragments synthesized using a stop primer. (b, c) Pulsed-field gel electrophoresis result of long DNA fragment concatenation.

Plasmid construction was performed using DNA fragments amplified with PCR stop primers (Fig. 5(a)). The stop primers, ODN_41 to ODN_44, were used in PCR to amplify fragment_LacZ as an insert coding the LacZ gene^49^ and fragment_AMPr as a vector backbone coding the ampicillin resistance gene.^50^ Two DNA fragments with sticky ends consisting of 15 bases were mixed and annealed, followed by gap filling and ligation using T4 DNA polymerase and T4 DNA ligase. The reaction mixture was transformed into competent *E. coli* cells and cultured on LB plates containing ampicillin, IPTG, and X-gal. When ligation proceeded and plasmids were formed, the *E. coli* cells exhibited ampicillin resistance, leading to colony formation. Additionally, the construction of correct plasmids would result in the expression of LacZ in the presence of IPTG, leading to the hydrolysis of X-gal in the plate and the formation of the blue pigment 5,5′-dibromo-4,4′-dichloro-indigo.^51,52^ Therefore, the presence of blue colonies indicated successful plasmid formation. Photographs of the LB plates taken 24 h after transformation are shown in Fig. 5(a). When plasmids were prepared using PCR stop primers, blue colonies were observed on the left side of Fig. 5(a), indicating successful ligation and plasmid construction. On the other hand, when the fragments were prepared using stop primers deprotected by photoirradiation before PCR, no colonies were formed, as shown in the right photograph of Fig. 5(a). This indicated a lack of ligation and failure in plasmid construction. Sixteen blue colonies were picked and cultured, and plasmids were extracted from them. The sequences of the obtained plasmids were analysed using the Sanger method. The results revealed that mutations occurred at three of the 32 ligation sites (Fig. S3, ESI†). The observed mutations were mismatch mutations that occurred during gap-filling by the polymerase or base skipping. However, the construction of plasmids with the correct sequence was confirmed in most of the samples. In addition, no mutations were observed in regions other than the ligation site. Thus, the 365 nm photoirradiation used to remove the *o*-nitrobenzyl modification did not induce DNA mutations. These results showed that plasmid construction can be achieved by synthesizing DNA fragments using PCR stop primers.

### Concatenation of long DNA fragments

We attempted to concatenate long DNA fragments in which restriction enzymes cannot generate sticky ends. The former and latter DNA fragments of 19 kbp each were amplified by PCR using ODN_34 and ODN_35 as stop primers. After fragment amplification by PCR, the *o*-nitrobenzyl groups were removed by photo-irradiation to prepare DNA fragments with sticky ends. As shown in Fig. 5(b), the two DNA fragments were mixed, annealed, and analyzed by pulse-field agarose gel electrophoresis. The result showed the formation of a 38 kbp product band, which corresponds to the hybridized product of the 19 kbp DNA fragments (lane C). Because the recognition sequence of restriction enzymes is typically six base pairs, the occurrence of the recognition sequence is calculated to be once per 4096 base pairs. For example, the recognition sequence of BsaI, used in the Golden Gate assembly, appears twice on this 19 kbp DNA fragment, other than the target region. Long DNA fragments are likely to contain recognition sequences in addition to the target sites when using restriction enzymes. In contrast, stop primers allow flexible design of the sequence and length of sticky ends, enabling the concatenation of long DNA fragments that are difficult with restriction enzymes.

Next, we attempted to construct a long genomic DNA using multiple simultaneous concatenations of DNA fragments, targeting lambda phage's 48.5 kbp genome DNA.^53^ Fragments 1 (9.5 kb), 2 (11.7 kb), 3 (14.3 kb), and 4 (13.2 kb) were synthesized using PCR with ODN_48 to ODN_53 as stop primers. These stop primers were designed such that the length of the DNA fragments was similar and the binding energy between the annealing regions was as low as possible. We initially used MFEprimer software^54^ to extract the potential primer regions. Subsequently, we employed the NSGA-II multi-objective genetic algorithm^55^ to select a suitable set of primers, considering both the length of DNA fragments and the binding energy. Binding energies were calculated using the UNAfold package.^56^ After amplification by PCR, DNA fragments with sticky ends were prepared following the same procedure as before. As shown in Fig. 5(c), the prepared fragments 1–4 were mixed and placed at room temperature or 50 °C for seven days. The time course of the reaction was analyzed by pulsed-field gel electrophoresis. The results are shown in Fig. 5(c). When the mixed DNA fragments were left standing at room temperature, a concatenation product of up to three fragments was observed after one day of reaction, but no full-length product composed of four fragments was observed. A slight observation of the full-length product was noted after allowing it to stand for three days, and a denser band at the full-length position was observed after seven days. In contrast, when the mixed DNA fragments were reacted at 50 °C, a band of the full-length product was observed one day later, unlike at room temperature. As the reaction time increased, the proportion of this band increased, and no significant change in band intensity was observed between days 5 and 7, with the final full-length product band intensity becoming comparable to that observed at room temperature. From the above results, it became clear that heating accelerates the concatenation between fragments when mixing and connecting four fragments, but an improvement in yield was not confirmed. However, it has been suggested that the simultaneous concatenation of multiple long DNA fragments is possible using stop primers. Further improvements in the yield are expected by optimizing the annealing conditions.

## Conclusions

Our study focused on developing PCR stop primers that enabled the creation of sticky ends in a PCR with a flexible design. To achieve this, we introduced highly bulky photo-protecting groups into the phosphate moiety of oligo DNA. We confirmed that introducing this modification into two consecutive bases effectively halts the polymerase chain elongation reaction for all types of bases. Using these PCR stop primers, we were able to construct sticky ends with a length of 50 bases and accomplish DNA concatenation reactions with greater efficiency than restriction enzymes. This high-efficiency concatenation reaction can be used to construct DNA libraries while maintaining their diversity. We also successfully created concatenated products of a 48.5 kbp lambda phage DNA using this method. Applying restriction enzymes is theoretically impossible for DNA of this length, as recognition sequences occur multiple times, probabilistically. These results demonstrated the superiority of the stop primers.

## Data availability

Experimental procedures, NMR spectra, and Fig. S1–S3 are available in the ESI.†

## Author contributions

The manuscript was written through contributions of H. A., F. H., and N. A. Amidite synthesis was performed by K. N., K. O., H. M., N. T., D. S., M. I, and Y. S. DNA oligo sequences were designed by Y. O., G. T., and K. A. Biological evaluations were conducted by K. N., T. N., and F. H. All authors have discussed the results and approved the final version of the manuscript.

## Conflicts of interest

The authors declare that they have no conflicts of interest.

## Supplementary Material

CB-005-D3CB00212H-s001
