# Supplementary material for: Development of PCR primers enabling the design of flexible sticky ends for efficient concatenation of long DNA fragments
Source: RSC Chem Biol. 2024 Feb 26;5(4):360–71. doi: 10.1039/d3cb00212h (PMC10989509; doi:10.1039/d3cb00212h)
Supplement: CB-005-D3CB00212H-s001 [file CB-005-D3CB00212H-s001.pdf]

Electronic Supplementary Material (ESI) for RSC Chemical Biology.
This journal is © The Royal Society of Chemistry 2024

Supporting information

Development of PCR Primer Enabling the Design of Flexible Sticky Ends for Efficient Concatenation of Long DNA Fragments

Kohei Nomura,[a] Kaoru Onda,[a] Hirotaka Murase,[a] Fumitaka Hashiya,[b] Yukiteru Ono,[c] Goro Terai,[c] Natsuhisa Oka,[d] Kiyoshi Asai,[c] Daisuke Suzuki,[a] Naho Takahashi,[a] Haruka Hiraoka,[a] Masahito Inagaki,[a] Yasuaki Kimura,[a] Yoshihiro Shimizu,[e] Naoko Abe[a] and Hiroshi Abe*[abfg]

[a]Department of Chemistry, Graduate School of Science, Nagoya University, Furo-cho, Chikusa-ku, Nagoya, Aichi 464-8602, Japan. Email: h-abe@chem.nagoya-u.ac.jp

[b]Research Center for Materials Science, Nagoya University, Furo-cho, Chikusa-ku, Nagoya, Aichi 464-8602, Japan

[c]Department of Computational Biology and Medical Sciences, Graduate School of Frontier Sciences, Uni-versity of Tokyo, Kashiwanoha, Kashiwa, Chiba 277-8561, Japan

[d]Department of Chemistry and Biomolecular Science Faculty of Engineering, Gifu University, Gifu 501-1193, Japan

[e]Laboratory for Cell-Free Protein Synthesis, RIKEN Center for Biosystems Dynamics Research, Suita, Osaka 565-0874, Japan

[f]CREST, Japan Science and Technology Agency, 7 Gobancho, Chiyoda-ku, Tokyo 102-0076, Japan

[g]Institute for Glyco-core Research (iGCORE), Nagoya University, Furo-cho, Chikusa-ku, Nagoya, Aichi 464-8601, Japan

*Corresponding authors

Contents

Experimental procedure and NMR spectra ……….…………….…………………………………S2
Supplementary Fig. S1 ……….…………………………………………………………………….S65
Supplementary Fig. S2 ……….…………………….…….………………………………….S66
Supplementary Fig. S3 ……….…………………..………….………………………………S67

General experimental details

Material

Reagents and solvents were purchased as reagent-grade, first-grade, or special-grade from TCI, FUJIFILM Wako, KANTO CHEMICAL and Sigma-Aldrich unless otherwise stated. All were used without further purification. Sterile water used in this research was produced by MicroPure UV (Thermo SCIENTIFIC) and Pacific TII 3 UV (Thermo SCIENTIFIC). For TLC, Silicagel 70 F254 TLC Plate-Wako was used. For silica gel column chromatography, Silica Gel 60 (spherical) 40-50 μm (KANTO CHEMICAL) was used.

NMR and MS spectra

NMR spectra were measured using JNM-ECS400 (JEOL) (400 MHz). The chemical shift values of 1H-NMR spectra were adjusted using the residual proton of the solvent (chloroform: 7.26 ppm, DMSO: 2.50 ppm) as an internal standard. The chemical shift values of 13C-NMR spectra were adjusted using the signals from the measured solvents (chloroform: 77.16 ppm, DMSO: 39.52 ppm) as an internal standard. The chemical shift values of 31P-NMR spectra were not standardized. The multiplicity of the signal is shown as s: singlet, d: doublet, t: triplet, q: quartet, sext: sextet, m: multiplet. MS spectra were measured using compact (Bruker) (ESI-MS).

Synthesis of phosphoramidite with *o*-nitrobenzyl group

Compound 1

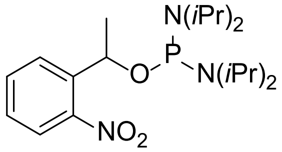

Bis(diisopropylamino)chlorophosphine (800 mg, 3.00 mmol, 1.0 eq.) and triethylamine (1.9 mL, 13.5 mmol, 4.5 eq.) were dissolved in THF (30 mL) and stirred at -10 °C. To the mixture, 1-(2-nitrophenyl)ethanol (431 μL, 3.20 mmol, 1.05 eq.) in THF (10 mL) was added dropwise then stirred at room temperature overnight. After that, the reaction mixture was filtered and concentrated. The crude was purified by silica gel column chromatography (Hexane/AcOEt = 1/1 with 3% TEA) and Compound **1** (770 mg, 1.94 mmol, 65%) was obtained. ^{1}H-NMR (392 MHz, $CDCl_3$): δ 7.922-7.872 (m, 2H), 7.627-7.585 (m, 1H), 7.384-7.341 (m, 1H), 5.349 (sext, J = 6.4 Hz, 1H), 3.602-3.411 (m, 4H), 1.549 (d, J = 6.4 Hz, 3H), 1.210-1.000 (m, 24H); ^{13}C-NMR (99 MHz, $CDCl_3$): δ 142.318, 133.314, 128.822, 127.496, 123.968, 67.058, 44.778, 25.350, 24.263; ^{31}P-NMR (159 MHz, $CDCl_3$): 113.234; HRMS (ESI) calc. m/z 398.2567 (M + H^+), found m/z 398.2600 (M + H^+).

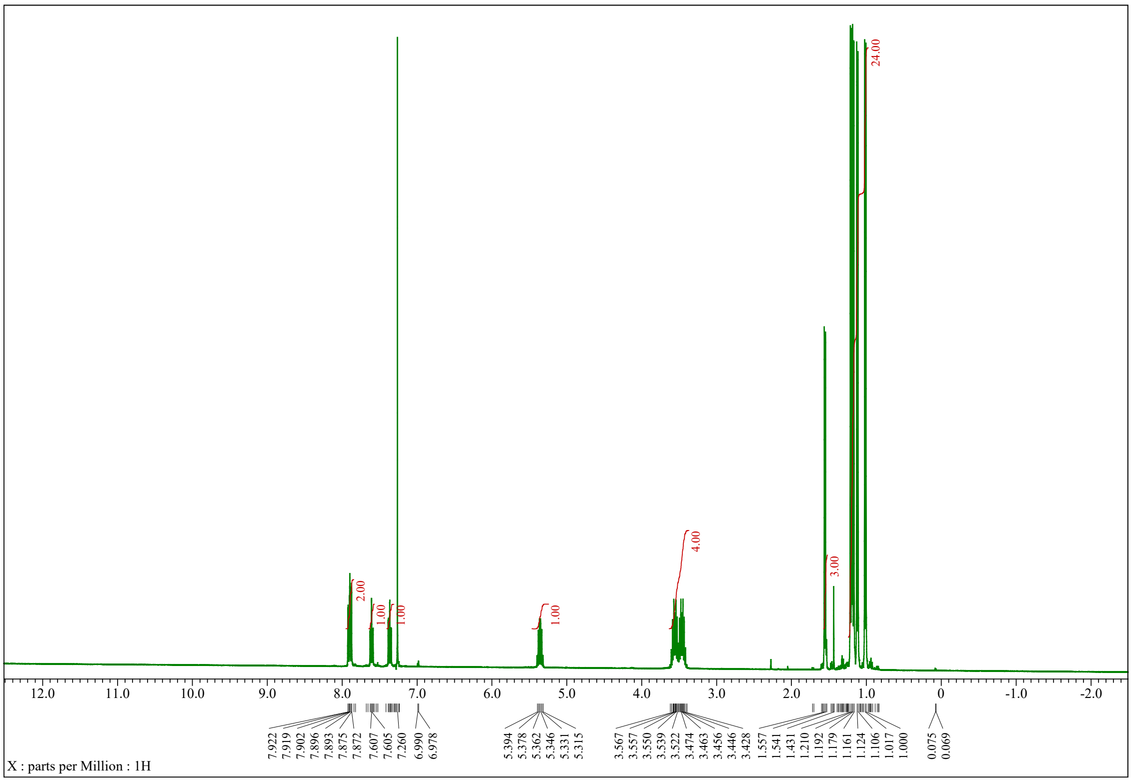

X : parts per Million : 1H

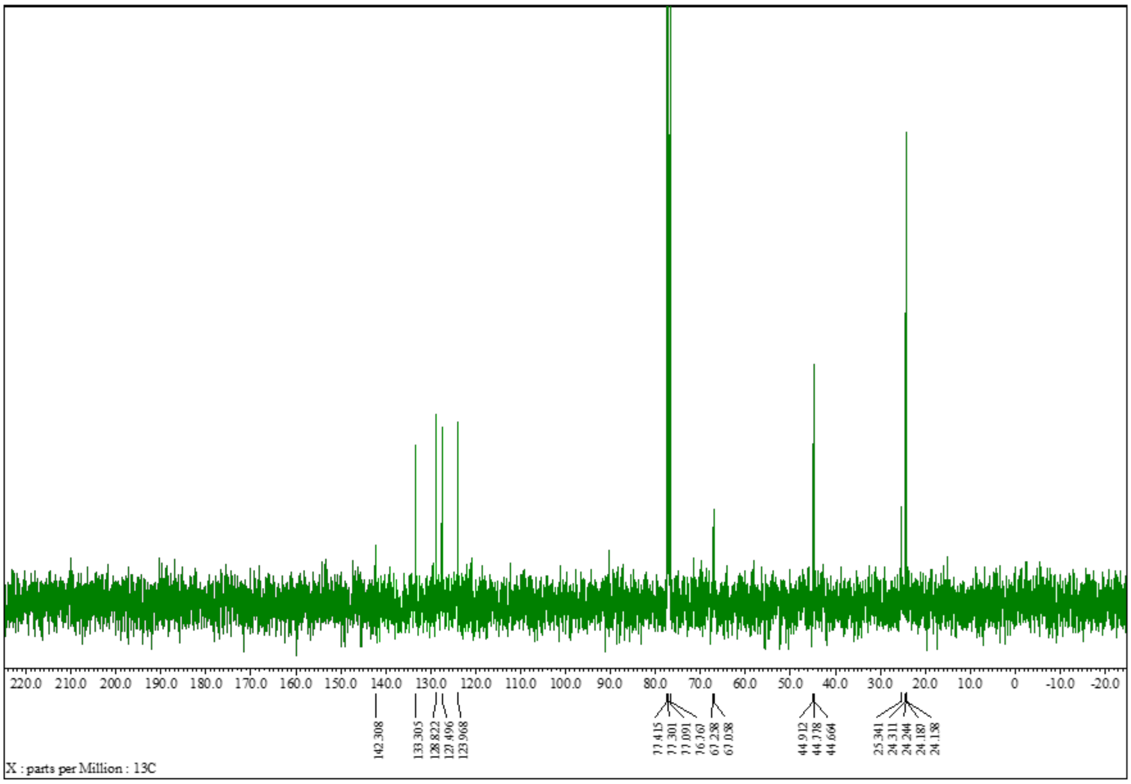

X : parts per Million : 13C

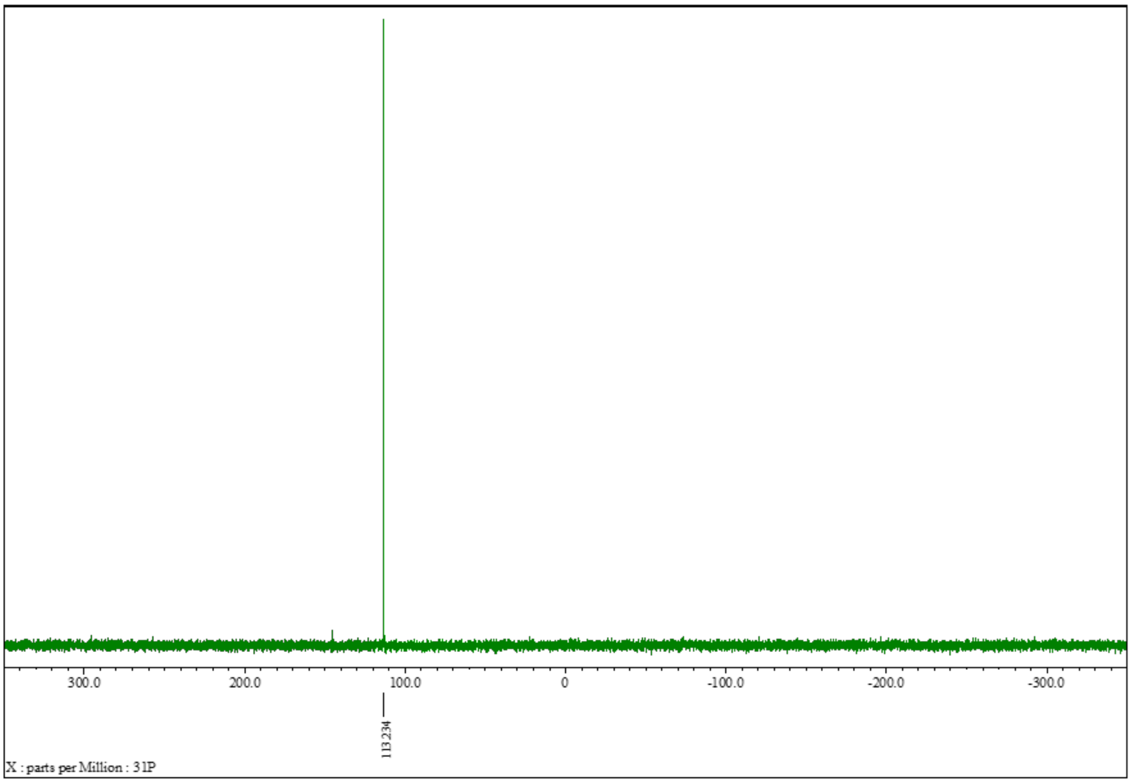

Compound 2

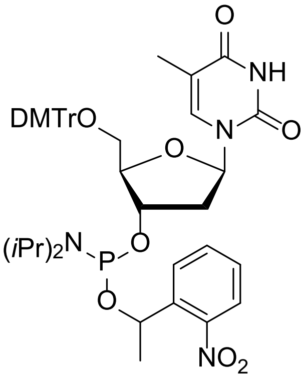

2'-deoxy-5'-*O*-(4,4'-dimethoxytrityl)-thymidine (343 mg, 0.63 mmol, 1.0 eq.) was dissolved in DCM (1.7 mL) and stirred at 0 °C. To the solution, Compound **1** (300 mg, 0.75 mmol, 1.2 eq.) and 1*H*-tetrazole (53.0 mg, 0.75 mmol, 1.2 eq.) were added then stirred at room temperature. After 4 hours, the reaction mixture was loaded onto a silica gel column directly. The product was purified by silica gel column chromatography (Hexane/AcOEt = 1/1 with 3% TEA) and compound **2** (309 mg, 0.37 mmol, 58%) was obtained. ^{1}H-NMR (392 MHz, $CDCl_3$) (diastereomixture): δ 7.886-7.471 (m, 4H), 7.402-7.172 (m, 10H), 6.825-6.769 (m, 4H), 6.450-6.415, 6.258-6.198 (m, 1H), 5.509-5.378 (m, 1H), 4.677-4.645, 4.498-4.436 (m, 1H), 4.263-3.914 (m, 1H), 3.771-3.759 (m, 6H), 3.636-3.176 (m, 4H), 2.580-2.130 (m, 2H), 1.578-0.836 (m, 18H); ^{13}C-NMR (99 MHz, $CDCl_3$): δ 163.720, 158.798,

144.340, 135.813, 135.355, 133.591, 133.362, 130.205, 128.651, 128.574, 128.250, 128.183, 128.069, 127.983, 127.239, 124.034, 113.333, 111.264, 87.039, 85.474, 81.564, 77.310, 67.172, 63.462, 55.327, 43.290, 40.267, 24.606, 14.287, 11.759; ^{31}P-NMR (159 MHz, $CDCl_3$): 148.752, 147.817, 147.710; HRMS (ESI) calc. *m*/*z* 863.3392 (M + Na^+), found *m*/*z* 863.3415 (M + Na^+).

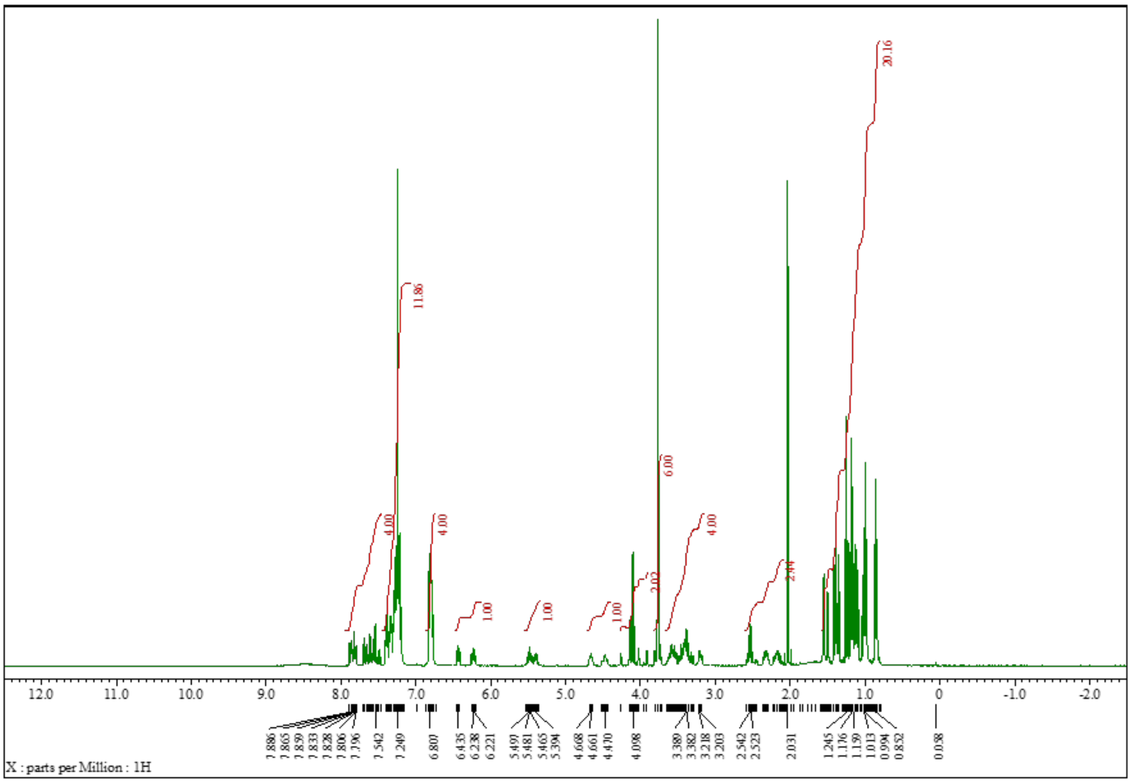

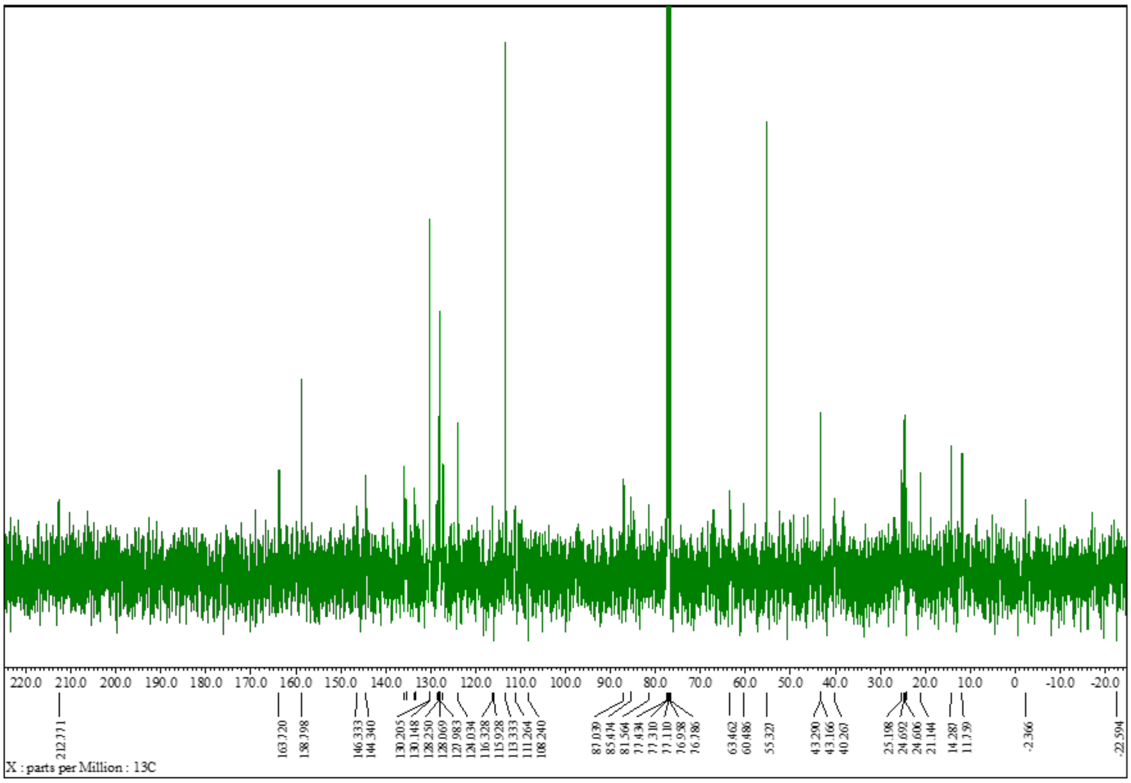
220.0 210.0 200.0 190.0 180.0 170.0 160.0 150.0 140.0 130.0 120.0 110.0 100.0 90.0 80.0 70.0 60.0 50.0 40.0 30.0 20.0 10.0 0 -10.0 -20.0
212.771
163.720
158.798
146.333
144.340
130.205
130.148
128.250
128.069
127.983
124.034
116.328
115.928
113.333
111.264
108.240
87.039
85.474
81.564
77.434
77.310
77.110
76.958
76.786
63.462
60.486
55.327
43.290
43.166
40.267
25.198
24.692
24.606
21.144
14.287
11.799
-2.366
-22.594
X : parts per Million : 13C

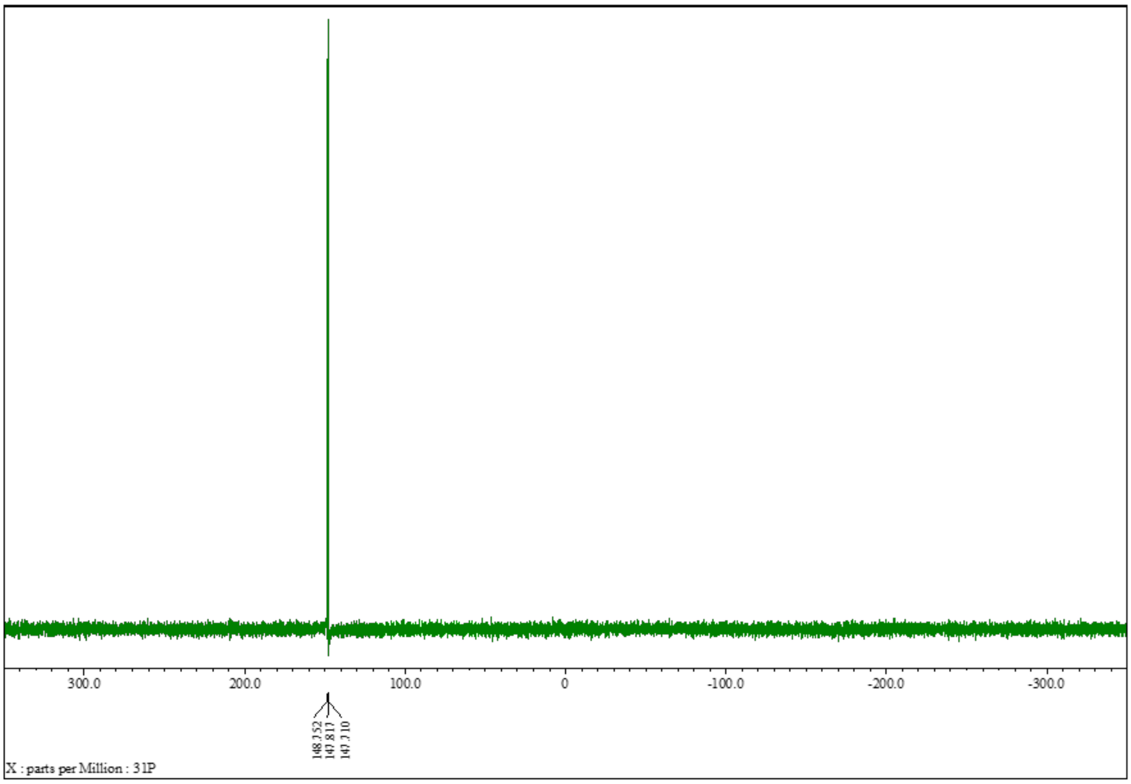
300.0 200.0 100.0 0 -100.0 -200.0 -300.0
148.552
147.817
147.110
X : parts per Million : 31P

Compound 3

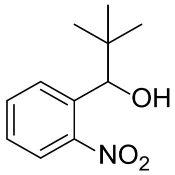

1-iodo-2-nitrobenzene (25.0 g, 100 mmol, 1.0 eq.) was dissolved in THF (150 mL) and cooled to -40 °C. 2 M phenylmagnesium chloride in THF solution (55.2 mL, 110 mmol, 1.1 eq.) was added dropwise and stirred at -40 °C. After stirring for 2 h, pivalaldehyde (10.9 mL, 100 mmol, 1.0 eq.) was added dropwise and stirred at - 40 °C. After stirring for 6 h, saturated NH_4Cl aqueous solution (200 mL) was added and warmed to room temperature. Brine (100 mL) was added and extracted with AcOEt. The organic layer was dried with Na_2SO_4, filtered, and concentrated in vacuo. The crude product was purified by silica gel column chromatography (Hexane/DCM = 94/6→80/20→6/94) and Compound **3** (15.5785 g, 74.5 mmol, 74%) was obtained. ^{1}H-NMR (392 MHz, $CDCl_3$): δ 7.797 (dd, *J* = 8.0, 1.6 Hz, 1H), 7.735 (dd, *J* = 8.2, 1.0 Hz, 1H), 7.604-7.561 (m, 1H), 7.413-7.370 (m, 1H), 5.361 (d, *J* = 3.2 Hz, 1H), 2.154 (d, *J* = 3.6 Hz, 1H), 0.874 (s, 9H); ^{13}C-NMR (99 MHz, $CDCl_3$): δ 149.693, 136,348, 131.941, 129.814, 128.022, 123.769, 74.401, 36.751, 25.590; HRMS (ESI) calc. m/z 232.0944 (M + Na^+), found m/z 232.0996 (M + Na^+).

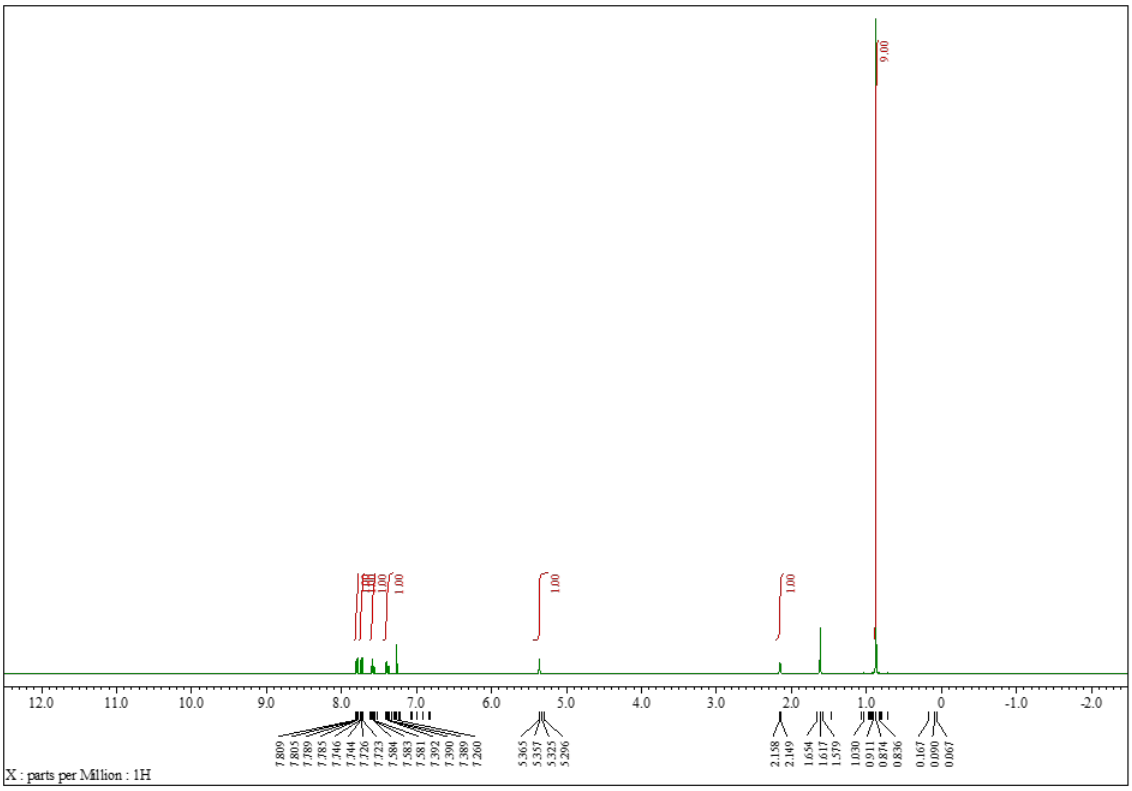

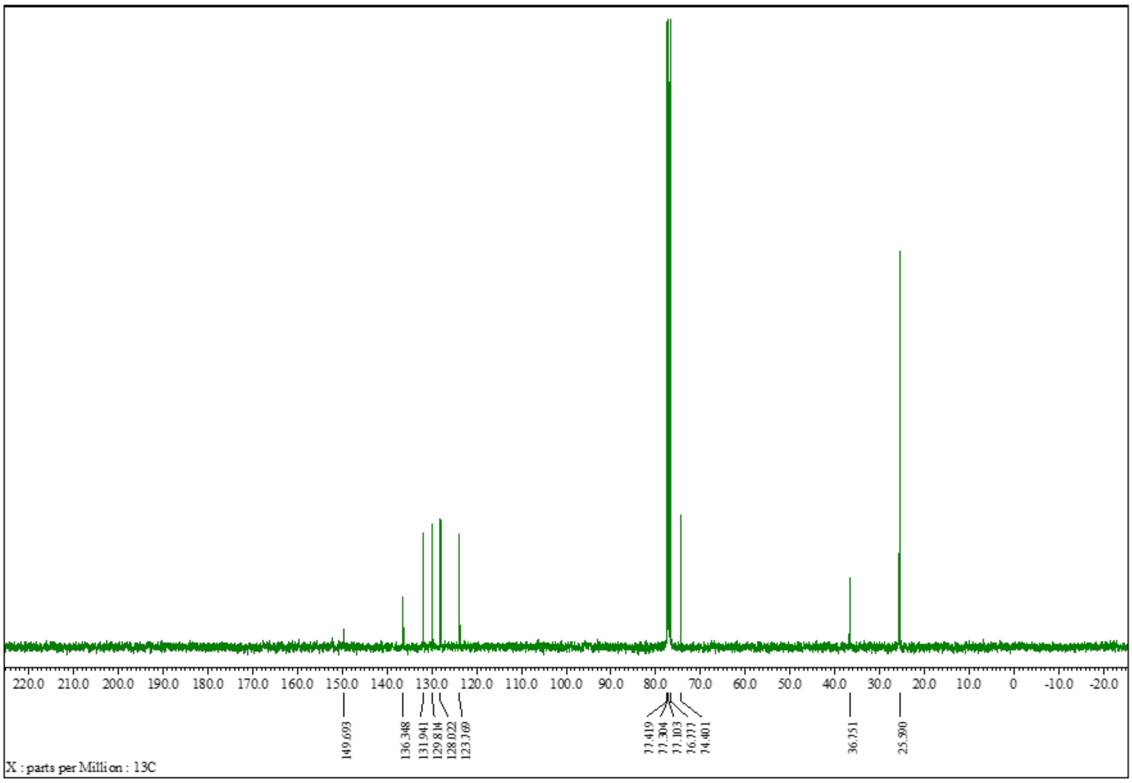

Compound 4

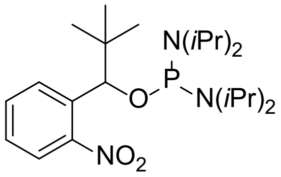

Compound **3** (3.00 g, 14.3 mmol, 1.0 eq.) was co-evaporated with pyridine and dissolved in THF (70 mL). Triethylamine (8.94 mL, 64.5 mmol, 4.5 eq.) was added and stirred at 0 °C. Bis(diisopropylamino)chlorophosphine (4.78 g, 17.9 mmol, 1.25 eq.) was added and stirred at 0 °C. After stirring for 1 h, stirring was further continued at room temperature for 17 h, then the reaction mixture was concentrated in vacuo. The crude product was purified by silica gel column chromatography (Hexane/AcOEt = 97/3→84:16 with 1% TEA) and Compound **4** (3.30 g, 7.51 mmol, 52%) was obtained. ^{1}H-NMR (392 MHz, $CDCl_3$): δ 7.891-7.721 (m, 2H), 7.556 (t, J = 7.8 Hz, 1H), 7.382-7.342 (m, 1H), 5.494 (2s, 1H), 3.655-3.511 (m, 2H), 3.497-3.402 (m, 2H), 1.209 (d, J = 6.8 Hz, 6H), 1.175 (d, J = 6.8 Hz, 6H), 1.138 (d, J = 7.2 Hz, 6H), 1.048 (d, J = 7.2 Hz, 6H), 0.903 (s, 9H); ^{13}C-NMR (99 MHz, $CDCl_3$): δ 149.280, 137.549, 131.788, 131.426, 127.554, 124.301, 75.908, 45.207, 37.749, 26.218, 24.645; ^{31}P-NMR (159 MHz, $CDCl_3$): δ 111.738; HRMS (ESI) calc. m/z 440.3037 ($M + H^+$), found m/z 440.3032 ($M + H^+$).

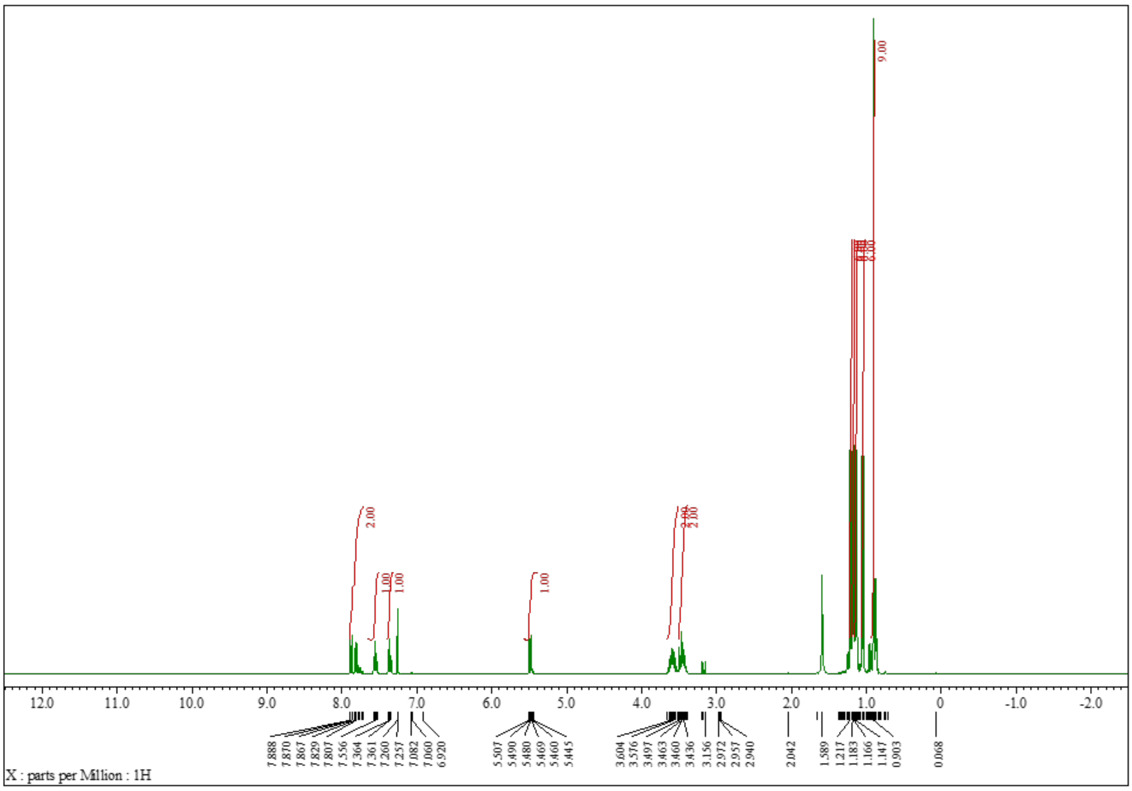
X : parts per Million : 1H

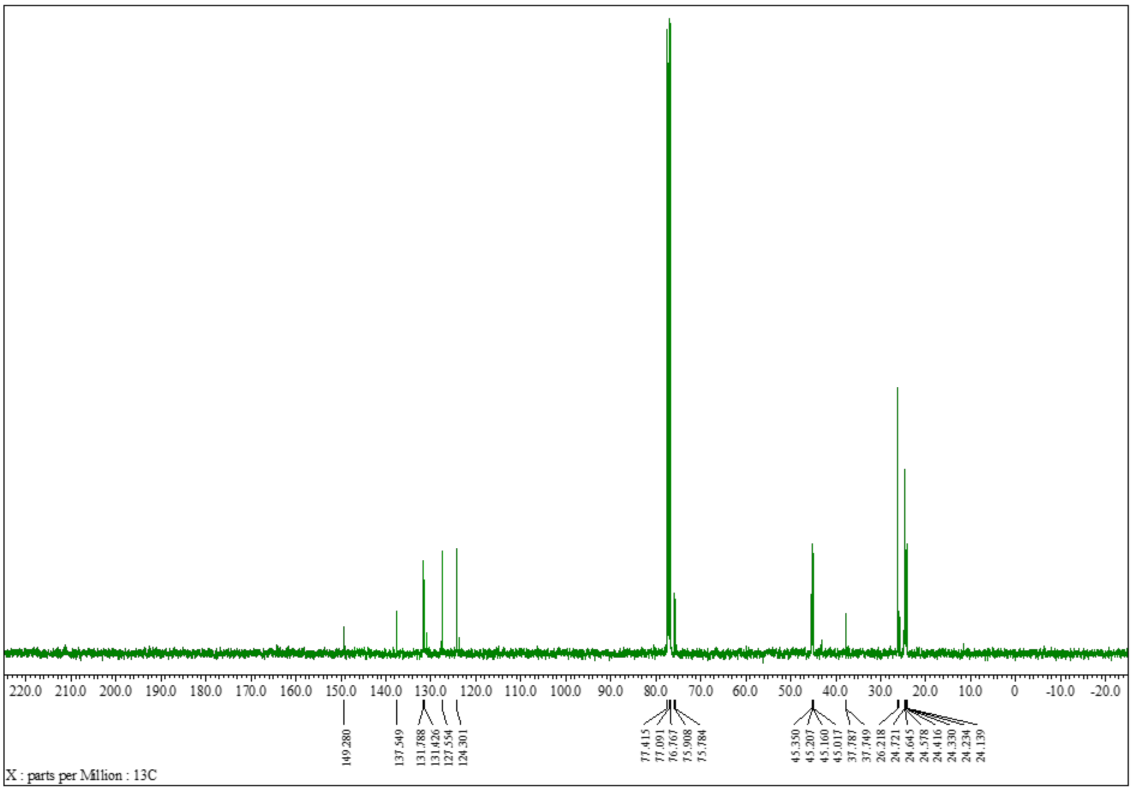
X : parts per Million : 13C

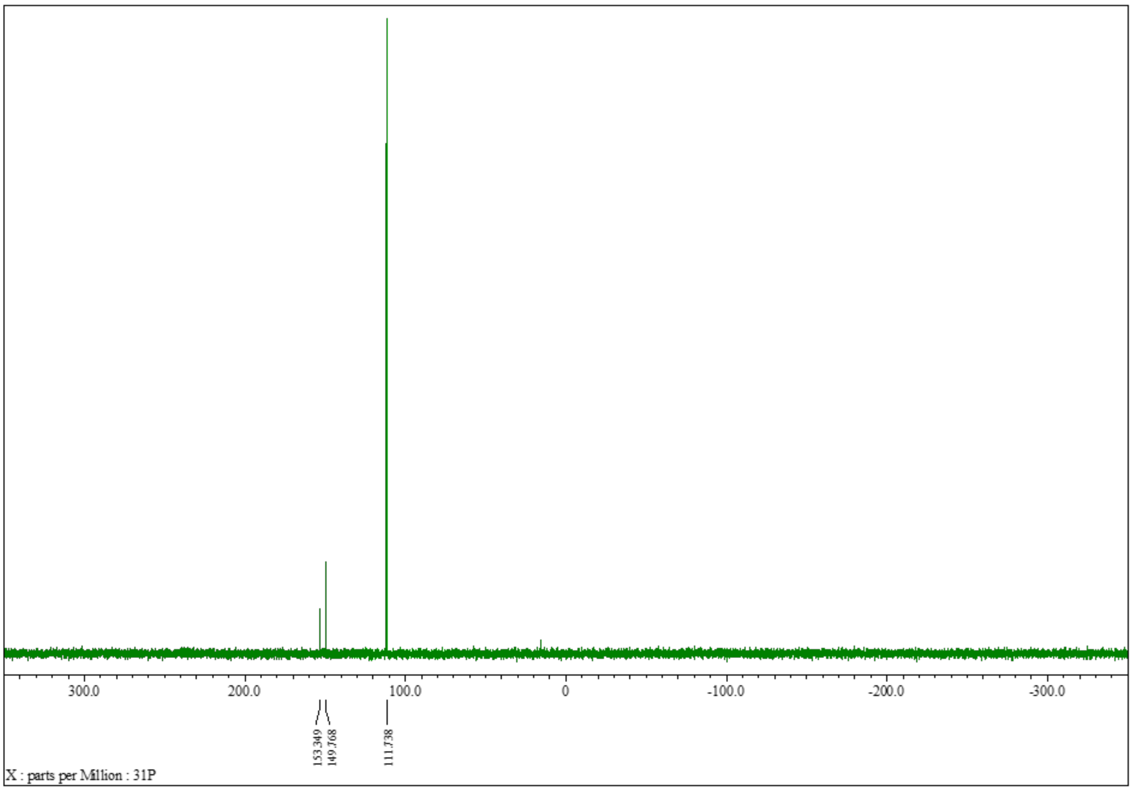

Compound 5

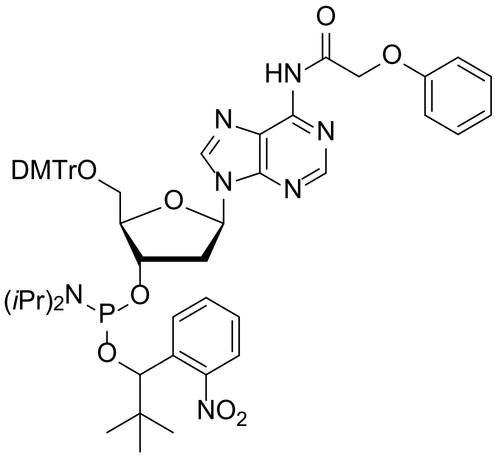

Compound **4** (2.56 g, 5.82 mmol, 2.0 eq.) and 5-(methylthio)-1*H*-tetrazole (1.01 g, 8.70 mmol, 3.0 eq.) were dissolved in DCM (7.5 mL). To the solution, 2'-deoxy-5'-*O*-(4,4'-dimethoxytrityl)-*N*6-phenoxyacetyladenosine (2.00 g, 2.91 mmol, 1.0 eq.) was added at 0 °C, then stirred at room temperature for 6 hours. Afterwards, AcOEt (200 mL) was added to the reaction mixture then washed with saturated $NaHCO_3$ aqueous solution and brine. The organic layer was dried with Na_2SO_4, filtered, and concentrated in vacuo. The crude product was purified by silica gel column chromatography (Hexane/AcOEt = 2/1→1/1 with 1% TEA) and Compound **5** (1.10 g, 1.07 mmol, 37 %) was obtained.

^{1}H-NMR (392 MHz, $CDCl_3$) (diastereomixture): δ 9.494 (br, 1H), 8.716-8.688 (m, 1H), 8.211-8.009 (m, 1H), 7.758-7.002 (m, 18H), 6.775-6.719 (m, 4H), 6.557-6.490, 5.931-5.722 (m, 1H), 5.491-5.393 (m, 1H), 4.857-4.487 (m, 3H), 4.336-4.306, 3.950-3.840 (m, 1H), 3.755-3.733 (m, 6H), 3.702-3.133 (m, 4H), 3.068-2.181 (m, 2H), 1.304-1.273 (m, 3H), 1.182-0.815 (m, 18H); ^{13}C-NMR (99 MHz, $CDCl_3$): δ 166.839, 158.598, 157.177, 152.361, 151.569, 149.442, 148.269, 144.616, 142.404, 141.917, 135.651, 131.483, 131.178, 130.119, 129.900, 128.155, 127.878, 127.811, 126.953, 123.910, 123.128, 122.451, 115.041, 113.200, 85.524, 84.750, 77.434, 75.718, 73.562, 68.240, 63.650, 55.279, 43.367, 39.504, 37.129, 25.675, 24.625; ^{31}P-NMR (159 MHz, $CDCl_3$): 152.601, 151.558, 148.164, 147.656; HRMS (ESI) calc. *m*/*z* 1026.4525 (M + H$^+$), found *m*/*z* 1026.4514 (M + H$^+$).

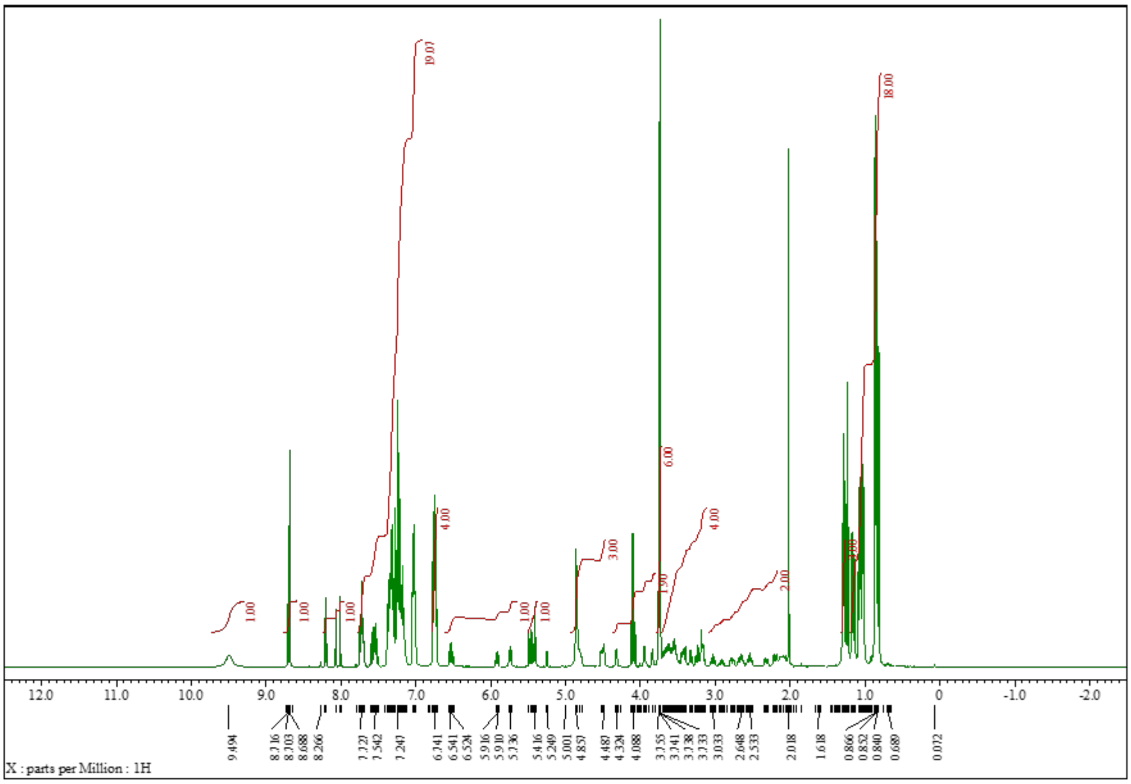

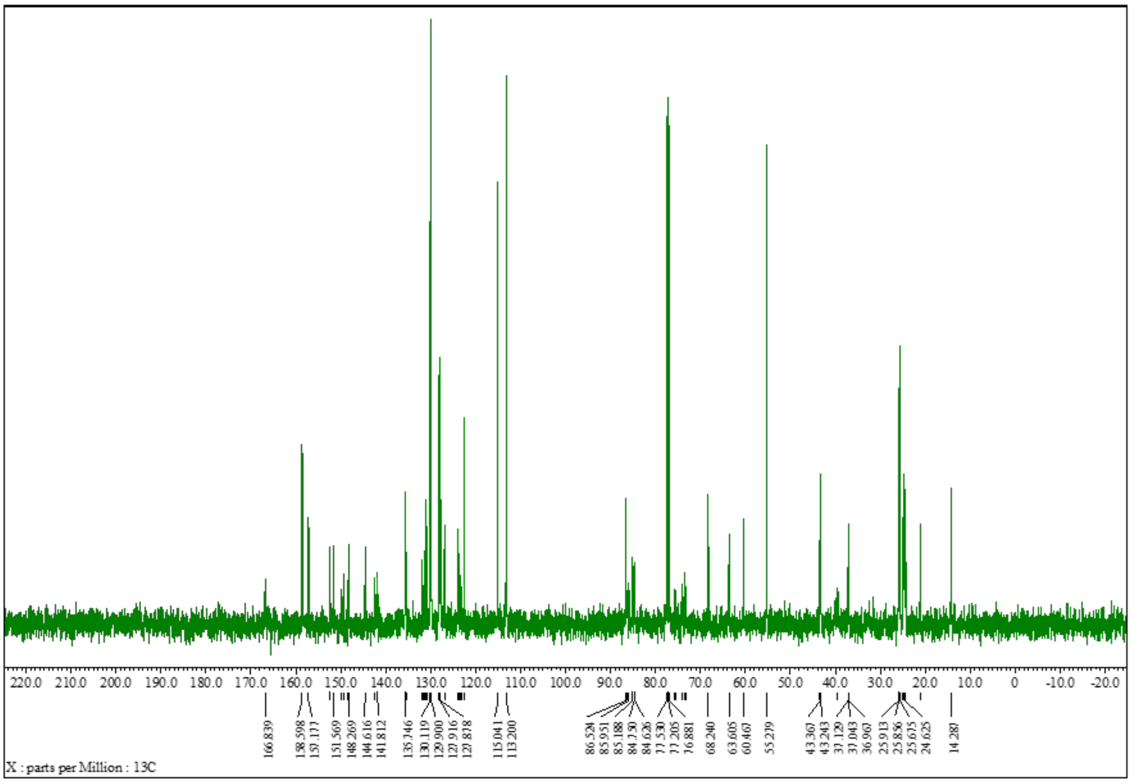
X : parts per Million : 13C
166.839
158.598
157.177
151.569
148.269
144.616
141.812
135.146
130.119
129.900
127.916
121.878
115.041
113.200
86.524
85.951
85.188
84.750
84.626
77.530
77.205
76.881
68.240
63.605
60.467
55.279
43.367
43.243
37.129
37.043
36.967
25.913
25.856
25.615
24.625
14.287

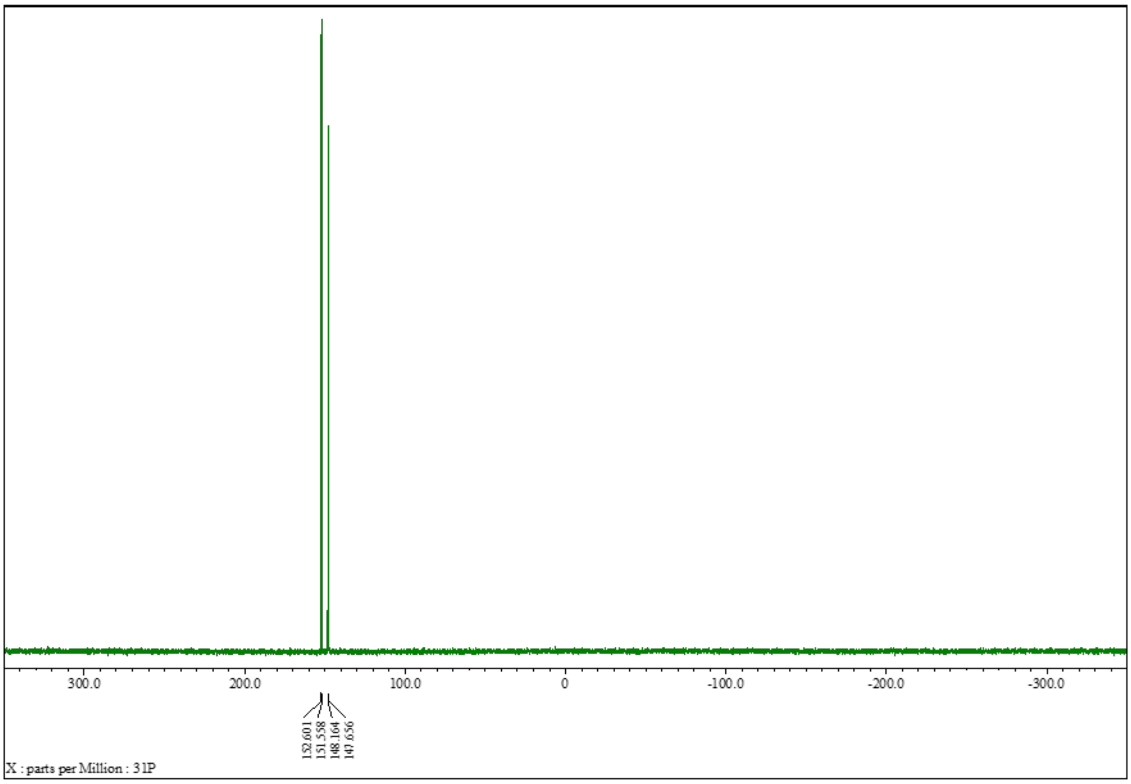
X : parts per Million : 31P
152.601
151.558
148.164
147.656

Compound 6

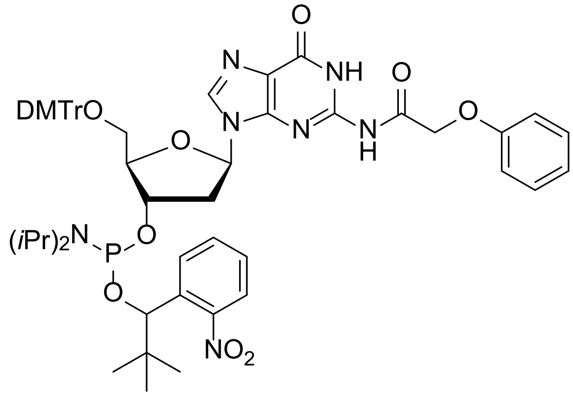

Compound **4** (2.50 g, 5.68 mmol, 2.0 eq.) and 5-(methylthio)-1*H*-tetrazole (0.990 g, 8.53 mmol, 3.0 eq.) were dissolved in DCM (10 mL). To the solution, 2'-deoxy-5'-*O*-(4,4'-dimethoxytrityl)-*N*2-phenoxyacetylguanosine (2.00 g, 2.84 mmol, 1.0 eq.) was added at 0 °C, then stirred at room temperature for 6 hours. Afterwards, AcOEt (200 mL) was added to the reaction mixture then washed with saturated $NaHCO_3$ aqueous solution and brine. The organic layer was dried with Na_2SO_4, filtered, and concentrated in vacuo. The crude product was purified by silica gel column chromatography (Hexane/AcOEt = 2/1→1/1 with 1% TEA) and Compound **6** (1.20 g, 1.15 mmol, 41 %) was obtained. ^{1}H-NMR (392 MHz, $CDCl_3$) (diastereomixture): δ 7.856-7.483 (m, 4H), 7.402-7.152 (m, 12H), 7.086-6.914 (m, 3H), 6.787-6.749 (m, 4H), 6.387-6.279, 5.802-5.686 (m, 1H), 5.458-5.301 (m, 1H), 4.738-4.613, 4.424-4.284, 3.928 (m, 4H), 3.795-3.711 (m, 6H), 3.689-3.510 (m, 2H), 3.399-3.289 (m, 1H), 3.171-3.080 (m, 1H), 2.762-2.551, 2.509-2.440, 2.157-2.088 (m, 2H), 1.322-0.822 (m, 21H); ^{13}C-NMR (99 MHz, $CDCl_3$): δ 170.186, 158.627, 156.662, 149.690, 148.727, 146.676, 144.549, 144.321, 137.082, 135.909, 135.603, 131.874, 130.692, 130.053, 129.890, 128.155, 127.954,127.764, 126.991, 123.968, 122.957, 122.012, 114.878, 113.238, 86.476, 85.999, 84.387, 83.548, 73.610, 67.086, 63.872, 55.307, 43.262, 40.362, 37.043, 25.608, 24.635; ^{31}P-NMR (159 MHz, $CDCl_3$): 151.050, 150.783, 147.282, 146.962; HRMS (ESI) calc. *m*/*z* 1064.4294 (M + Na^+), found *m*/*z* 1064.4245 (M + Na^+).

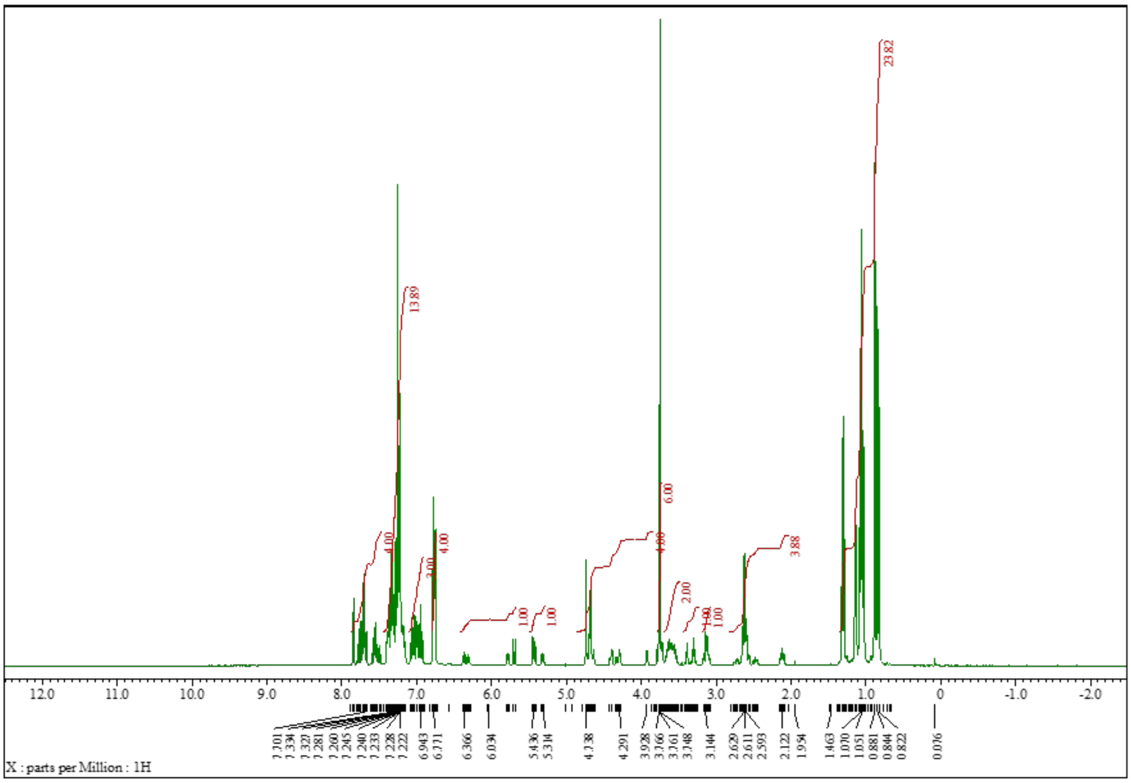
12.0 11.0 10.0 9.0 8.0 7.0 6.0 5.0 4.0 3.0 2.0 1.0 0 -1.0 -2.0
X : parts per Million : 1H

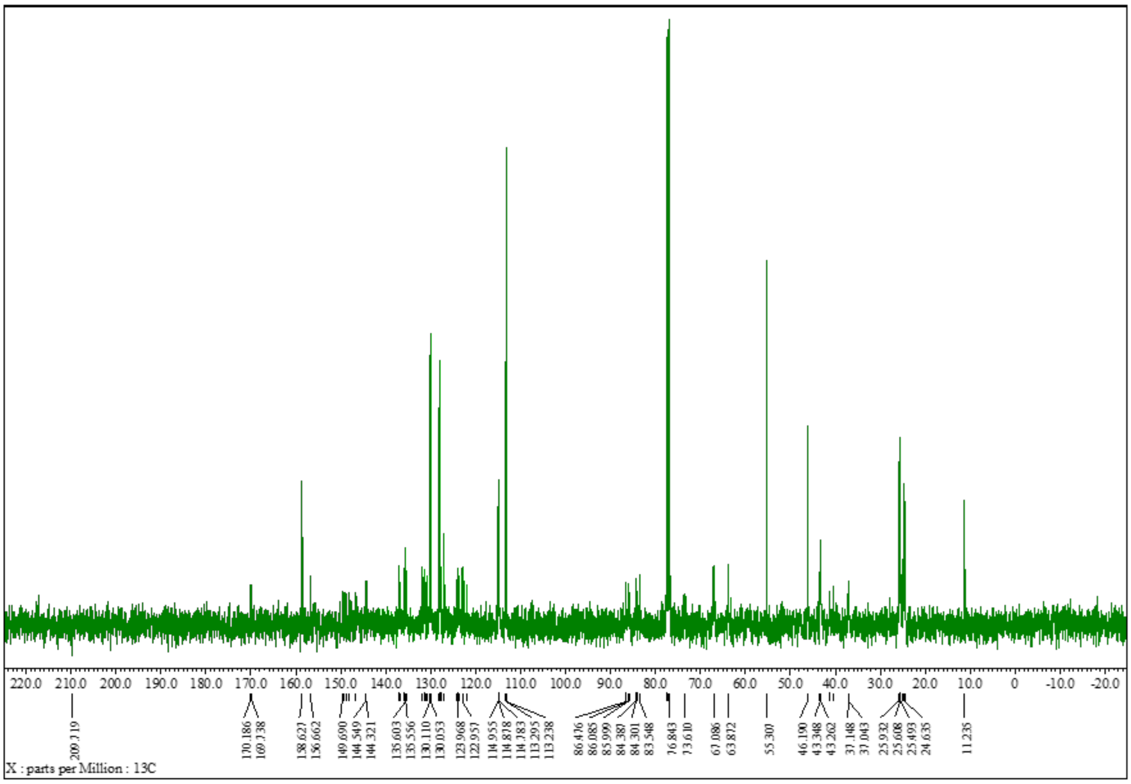
220.0 210.0 200.0 190.0 180.0 170.0 160.0 150.0 140.0 130.0 120.0 110.0 100.0 90.0 80.0 70.0 60.0 50.0 40.0 30.0 20.0 10.0 0 -10.0 -20.0
X : parts per Million : 13C

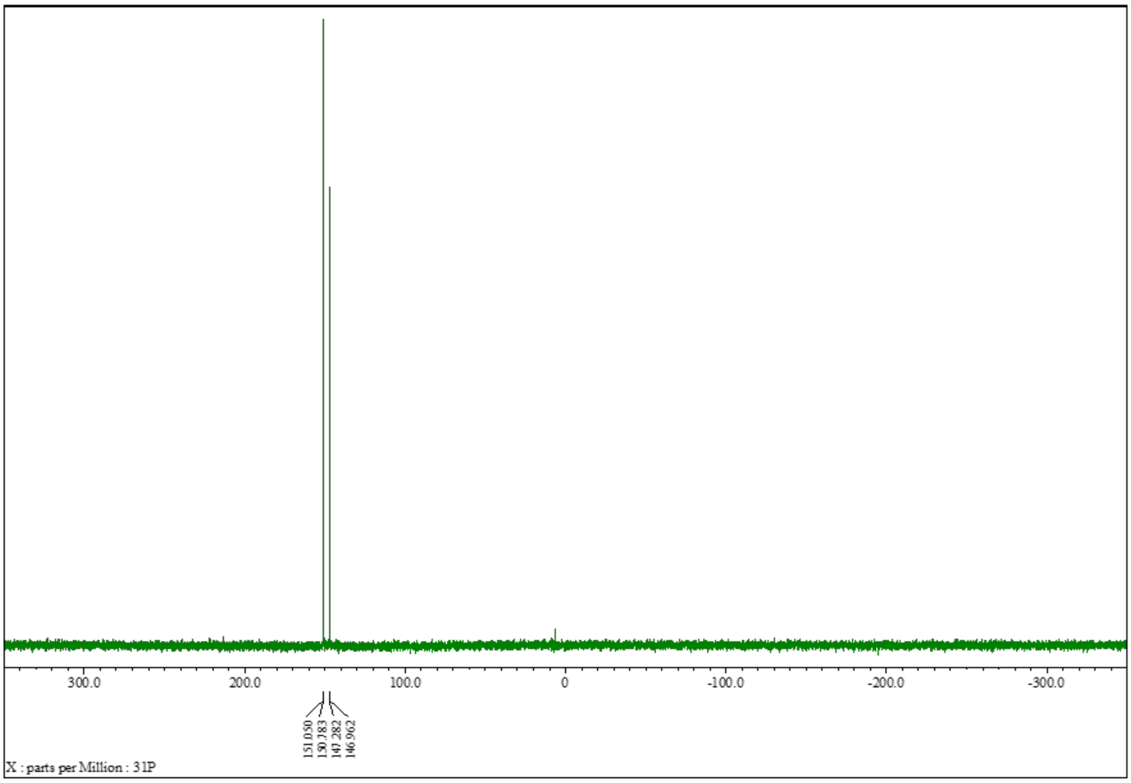

Compound 7

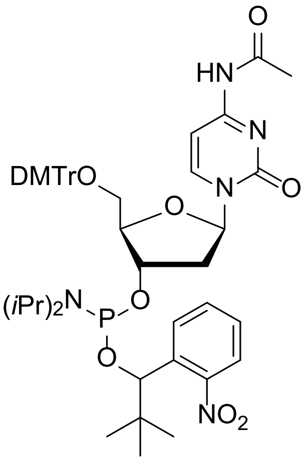

Compound **4** (2.77 g, 6.30 mmol, 2.0 eq.) and 5-(methylthio)-1*H*-tetrazole (0.731 g, 6.30 mmol, 2.0 eq.) were dissolved in DCM (10 mL). To the solution, *N*4-acetyl-2'-deoxy-5'-*O*-(4,4'-dimethoxytrityl)-cytidine (1.80 g, 3.15 mmol, 1.0 eq.) was added at 0 °C, then stirred at room temperature for 6 hours. Afterwards, AcOEt (200 mL) was added to the reaction mixture then washed with saturated $NaHCO_3$ aqueous solution and brine. The organic layer was dried with Na_2SO_4, filtered, and concentrated in vacuo. The crude product was purified by silica gel column chromatography (Hexane/AcOEt = 1/1 with 1% TEA) and Compound **7** (2.12 g, 2.32 mmol, 74 %) was obtained. ^{1}H-NMR (392 MHz, $CDCl_3$)

(diastereomixture): δ 9.899-9.802 (br, 1H), 8.234-7.991 (m, 1H), 7.770-7.488 (m, 3H), 7.395-7.212 (m, 10H), 7.120-7.044 (m, 1H), 6.846-6.783 (m, 4H), 6.345-6.256, 5.793-5.722 (m, 1H), 5.465-5.338 (m, 1H), 4.744-4.636, 4.419-4.339 (m, 1H), 4.246-4.207, 3.938-3.912, 3.719-3.712 (m, 1H), 3.801-3.779 (m, 6H), 3.675-3.175 (m, 4H), 2.878-2.726, 2.432-2.282 (m, 1H), 2.259-2.241 (m, 3H), 2.215-2.076, 1.760-1.694 (m, 1H), 1.293-0.797 (m, 21H); ^{13}C-NMR (99 MHz, $CDCl_3$): δ 170.873, 162.804, 158.713, 154.840, 149.375, 144.654, 144.330, 135.374, 131.455, 130.920, 130.167, 128.174, 128.031, 127.802, 127.144, 123.882, 123.386, 113.333, 96.404, 87.000, 86.924, 85.856, 77.339, 72.809, 62.709, 55.317, 43.443, 41.287, 37.024, 25.684, 24.959, 24.664; ^{31}P-NMR (159 MHz, $CDCl_3$): 153.295, 151.799, 149.955, 147.576; HRMS (ESI) calc. *m*/*z* 910.4151 (M + H$^+$), found *m*/*z* 910.4133 (M + H$^+$).

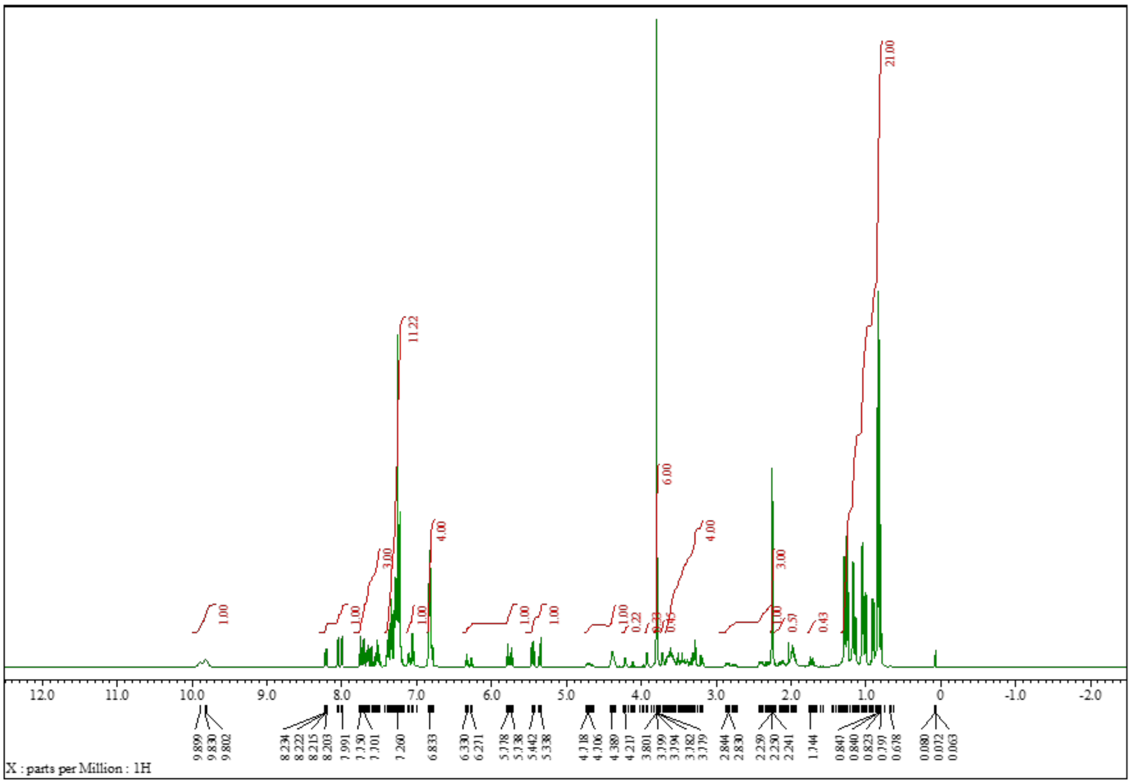

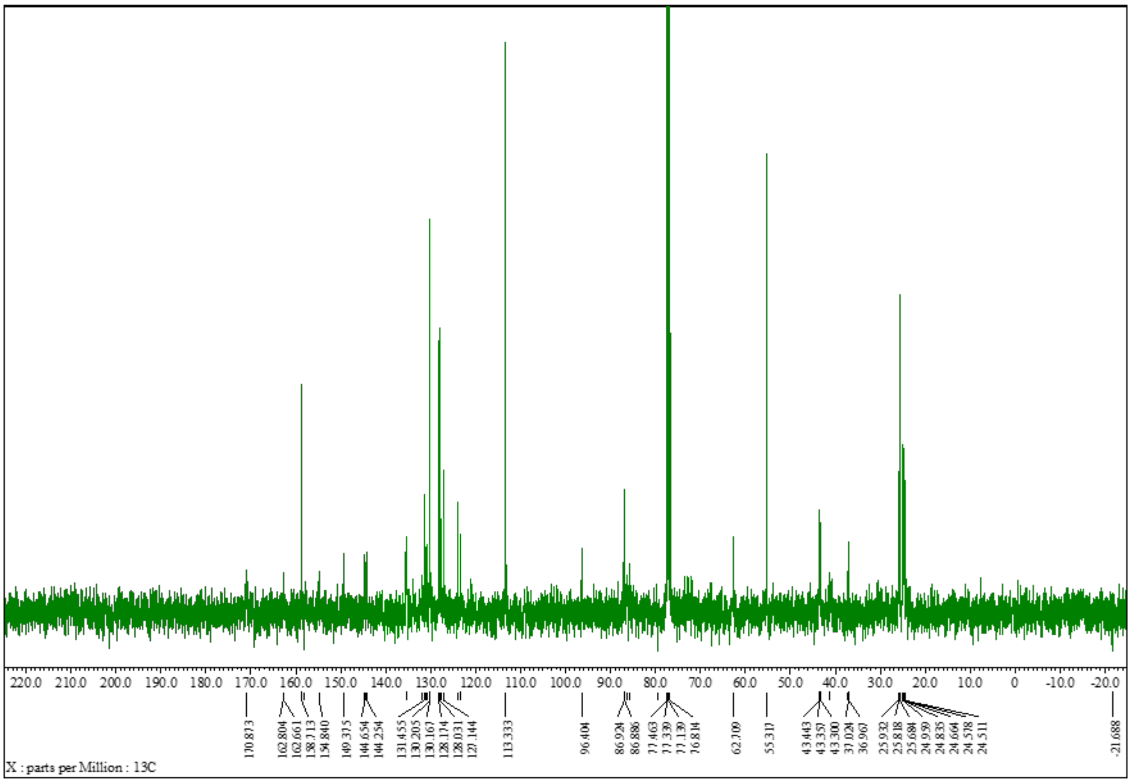
X : parts per Million : 13C
170.873
162.804
162.661
158.713
154.840
149.375
144.654
144.254
131.455
130.205
130.167
128.174
128.031
127.144
113.333
96.404
86.924
86.886
77.463
77.339
77.139
76.814
62.109
55.317
43.443
43.357
43.300
37.024
36.967
25.932
25.818
25.684
24.959
24.835
24.664
24.578
24.511
-21.688

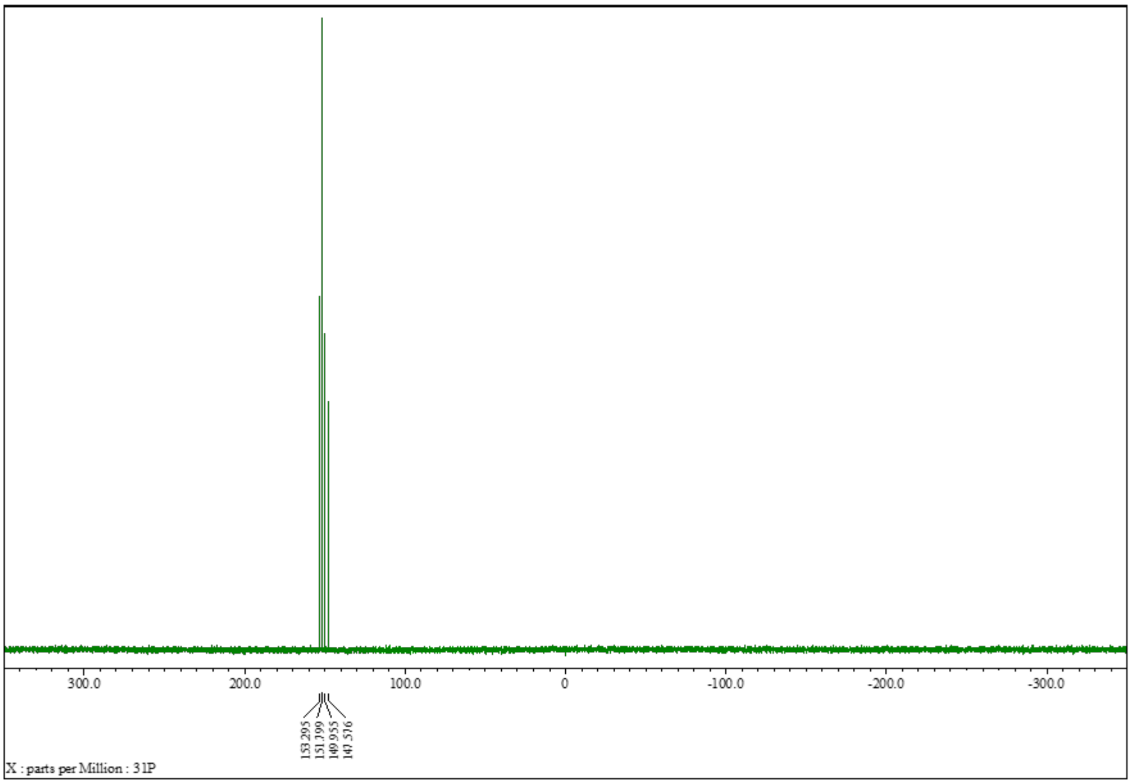
X : parts per Million : 31P
153.295
151.399
149.955
147.576

Compound 8

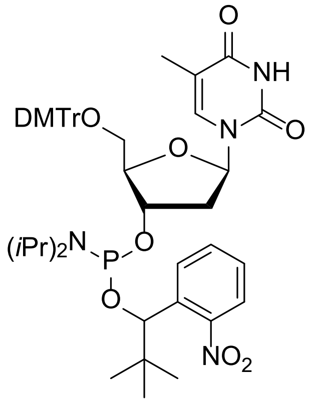

Compound **4** (2.42 g, 5.51 mmol, 2.0 eq.) and 5-(methylthio)-1*H*-tetrazole (0.640 g, 5.51 mmol, 2.0 eq.) were dissolved in DCM (10 mL). To the solution, 2'-deoxy-5'-*O*-(4,4'-dimethoxytrityl)-thymidine (1.50 g, 2.75 mmol, 1.0 eq.) was added at 0 °C, then stirred at room temperature for 7 hours. Afterwards, AcOEt (200 mL) was added to the reaction mixture then washed with saturated $NaHCO_3$ aqueous solution and brine. The organic layer was dried with Na_2SO_4, filtered, and concentrated in vacuo. The crude product was purified by silica gel column chromatography (Hexane/AcOEt = 2/1→1/1 with 1% TEA) and Compound **8** (2.00 g, 2.2 mmol, 81 %) was obtained. ^{1}H-NMR (392 MHz, $CDCl_3$) (diastereomixture): δ 8.528 (br, 1H), 7.774-7.192 (m, 14H), 6.812-6.777 (m, 4H), 6.489-6.414, 5.816-5.710 (m, 1H), 5.469-5.281 (m, 1H), 4.775-4.691, 4.441-4.414 (m, 1H), 4.324-3.801 (m, 1H), 3.779-3.763 (m, 6H), 3.724-3.085 (m, 4H), 2.662-1.873 (m, 2H), 1.412-0.773 (m, 24H); ^{13}C-NMR (99 MHz, $CDCl_3$): δ 164.006, 158.770, 150.463, 144.387, 135.832, 135.422, 135.250, 131.817, 131.512, 130.816, 130.138, 128.231, 128.050, 127.191, 123.939, 123.262, 113.324, 111.330, 86.953, 85.541, 84.511, 78.350, 73.705, 63.834, 55.327, 43.386, 40.305, 36.948, 25.646, 24.482, 11.635; ^{31}P-NMR (159 MHz, $CDCl_3$): 152.921, 151.665, 149.474, 147.897; HRMS (ESI) calc. *m*/*z* 905.3861 (M + Na^+), found *m*/*z* 905.3862 (M + Na^+).

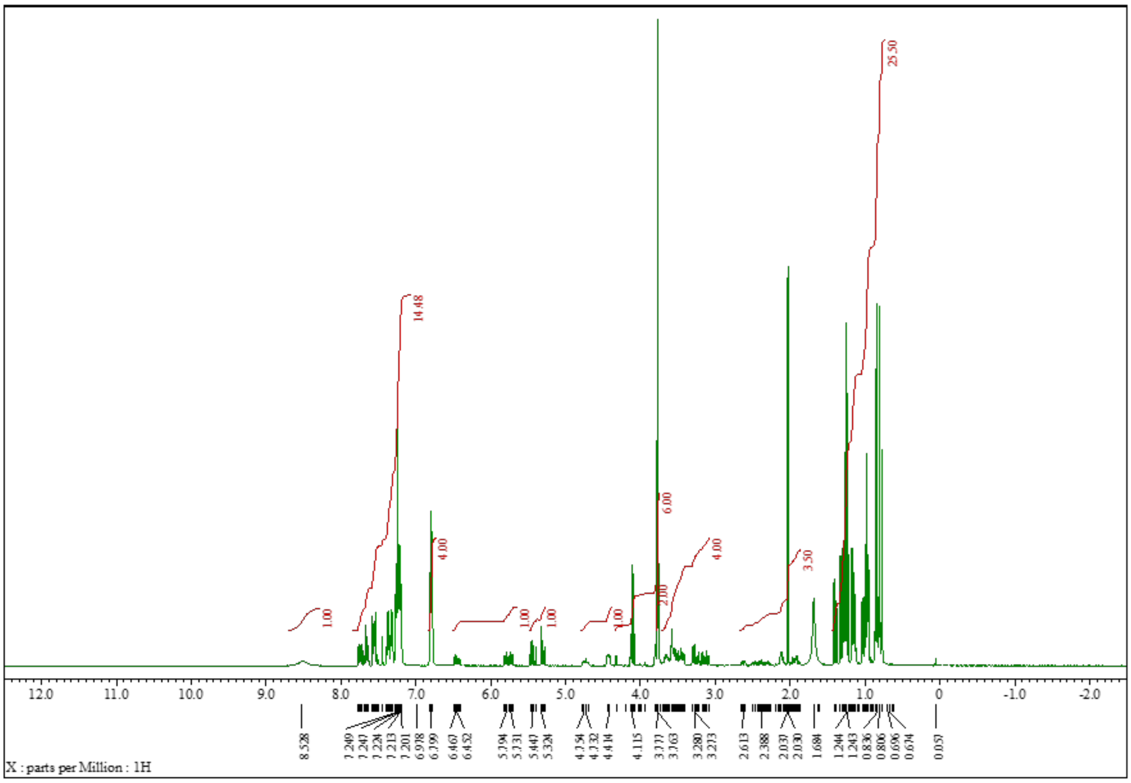
14.48
4.00
6.00
4.00
25.50
1.00
1.00
1.00
1.00
2.00
3.50
8.528
7.249
7.247
7.224
7.213
7.201
6.978
6.799
6.467
6.452
5.794
5.731
5.447
5.324
4.754
4.732
4.414
4.115
3.777
3.763
3.280
3.213
2.613
2.388
2.037
2.030
1.684
1.244
1.243
0.836
0.806
0.696
0.674
0.057
X : parts per Million : 1H

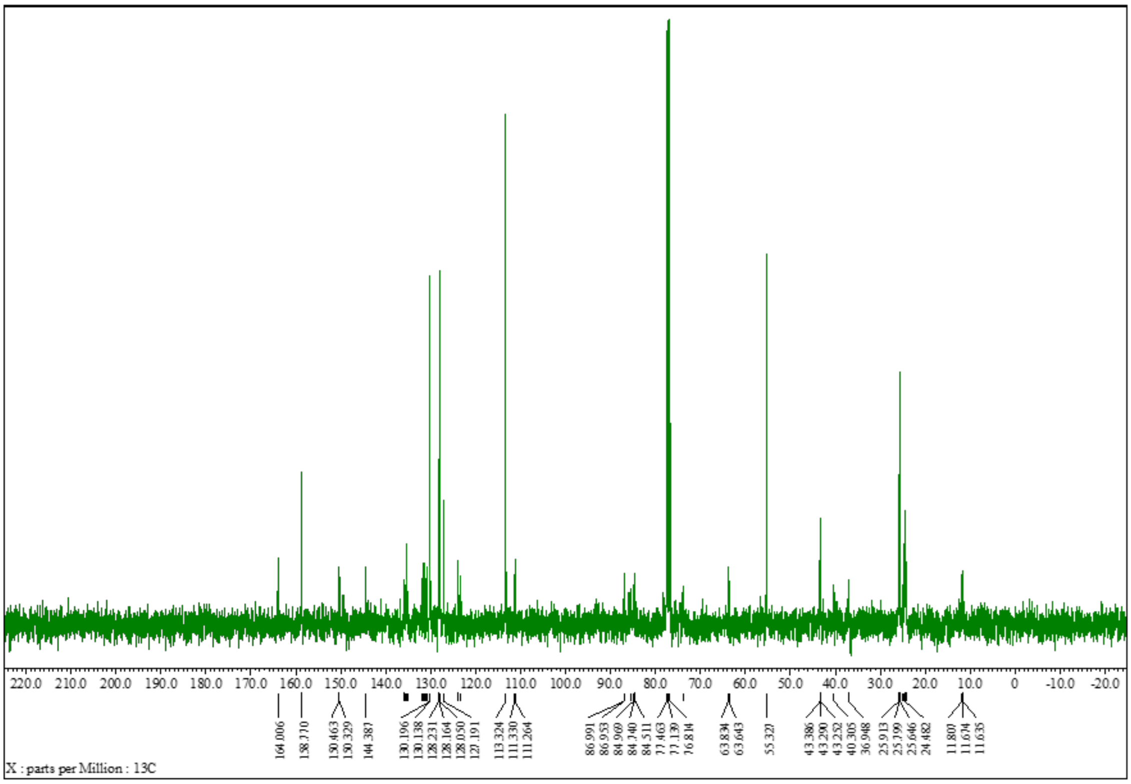
164.006
158.770
150.463
150.329
144.387
130.196
130.138
128.231
128.164
128.050
127.191
113.324
111.330
111.264
86.991
86.953
84.969
84.740
84.511
77.463
77.139
76.814
63.834
63.643
55.327
43.386
43.290
43.252
40.305
36.948
25.913
25.799
25.646
24.482
11.807
11.674
11.635
X : parts per Million : 13C

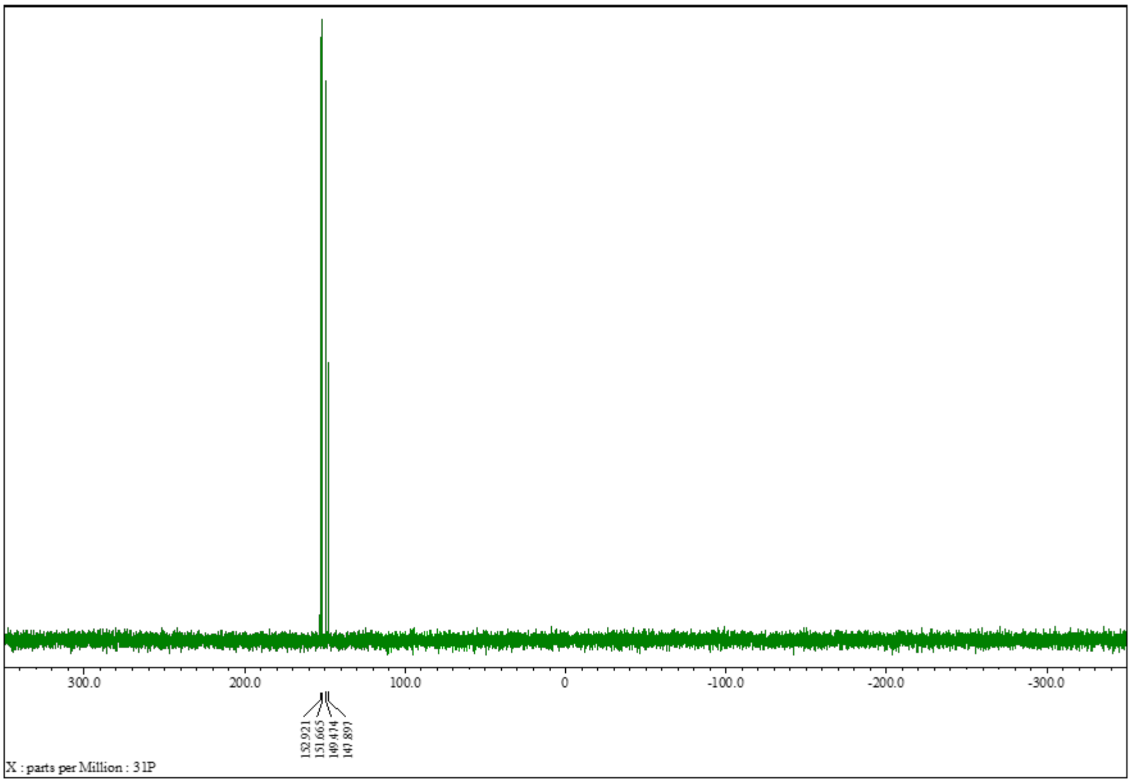

Compound 9

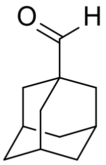

1-adamantanemethanol (1.80 g, 10.8 mmol, 1.0 eq.) was dissolved in DCM (160 mL). Then, PCC (3.50 g, 16.2 mmol, 1.5 eq.) was added and stirred at room temperature for 2 hours. After that, the reaction mixture was loaded directly to a silica gel column (about 10 cm). The sample was eluted by DCM and concentrated in vacuo. Compound **9** (1.60 g, 9.74 mmol, 90%) was obtained. ^{1}H-NMR (392 MHz, $CDCl_3$): δ 9.321 (s, 1H), 2.066 (m, 3H), 1.793-1.671 (m, 12H); ^{13}C-NMR (99 MHz, $CDCl_3$): δ 206.095, 44.921, 36.643, 35.918, 27.429; HRMS (ESI) calc. m/z 187.1093 (M +Na^+), found m/z 187.1092 (M + Na^+).

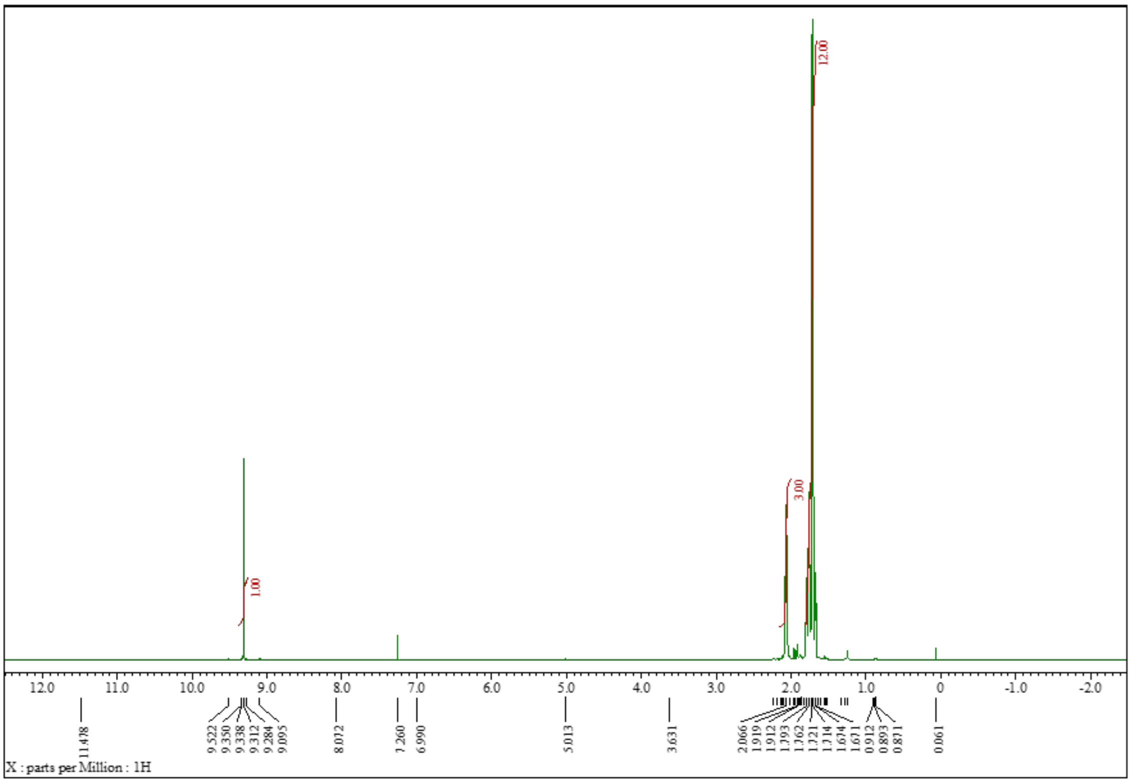
12.00
3.00
1.00
X : parts per Million : 1H

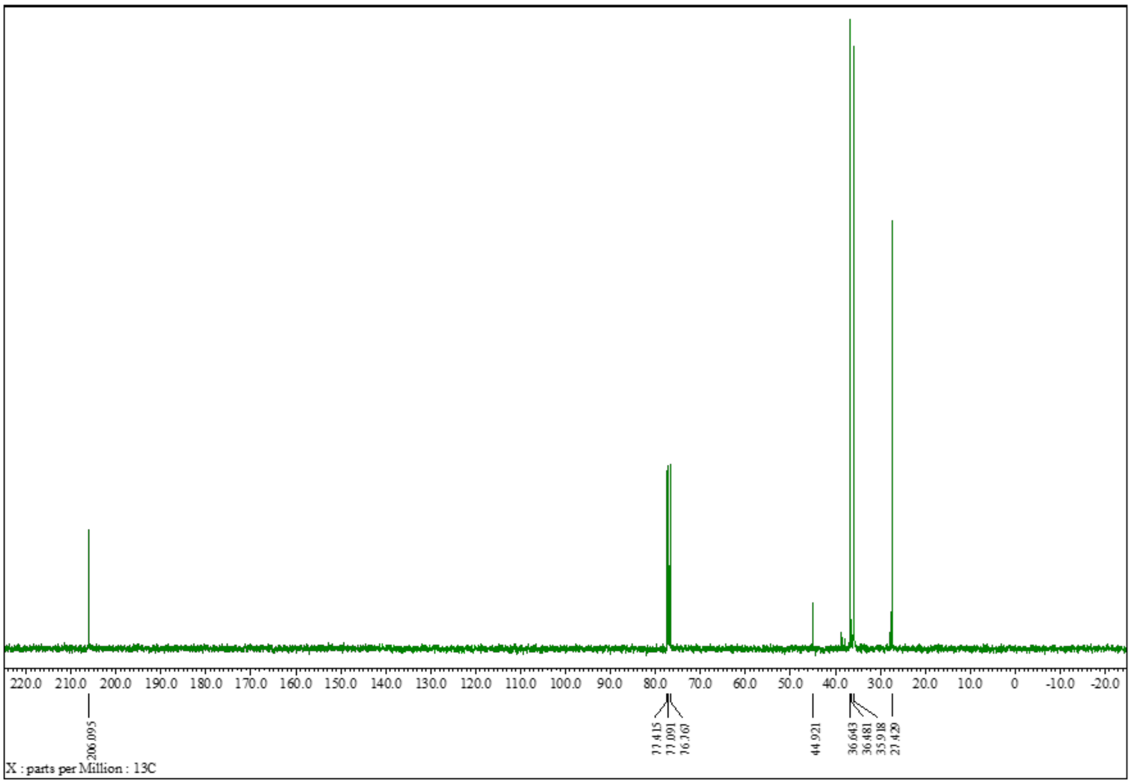
X : parts per Million : 13C

Compound 10

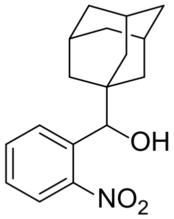

1-iodo-2-nitrobenzene (758 mg, 3.00 mmol, 1.0 eq.) was dissolved in THF (28 mL) and stirred at -40 °C. To the solvent, 2 M phenylmagnesium chloride in THF solution (1.65 mL, 3.30 mmol, 1.1 eq.) was added dropwise and stirred at -40 °C for 30 min. Then Compound **9** (500 mg, 3.00 mmol, 1.0 eq.) in THF (2 mL) was added. After stirring for 2 hours, the reaction was quenched by adding saturated NH_4Cl aqueous solution. The temperature of reaction mixture was gradually increased to room temperature and diluted with water. After extraction by AcOEt, the organic layer was washed with brine, dried by Na_2SO_4, and concentrated in vacuo. After purification by silica gel column chromatoghraphy (Hexane/DCM = 1/2), Compound **10** (300 mg, 1.04 mmol, 35%) was obtained. ^{1}H-NMR (392 MHz, $CDCl_3$): δ 7.753-7.713 (m, 2H), 7.605-7.563 (m, 1H), 7.418-7.375 (m, 1H), 5.210 (d, J = 3.6 Hz, 1H), 2.013 (d, J = 4.0 Hz, 1H), 1.941 (m, 3H), 1.659-1.401 (m, 12H); ^{13}C-NMR (99 MHz, $CDCl_3$): δ 135.203, 131.722, 130.138, 127.945, 123.710, 75.126, 38.350, 37.558, 36.891, 28.250 ; HRMS (ESI) calc. m/z 310.1414 (M + Na^+), found m/z 310.1407 (M + Na^+).

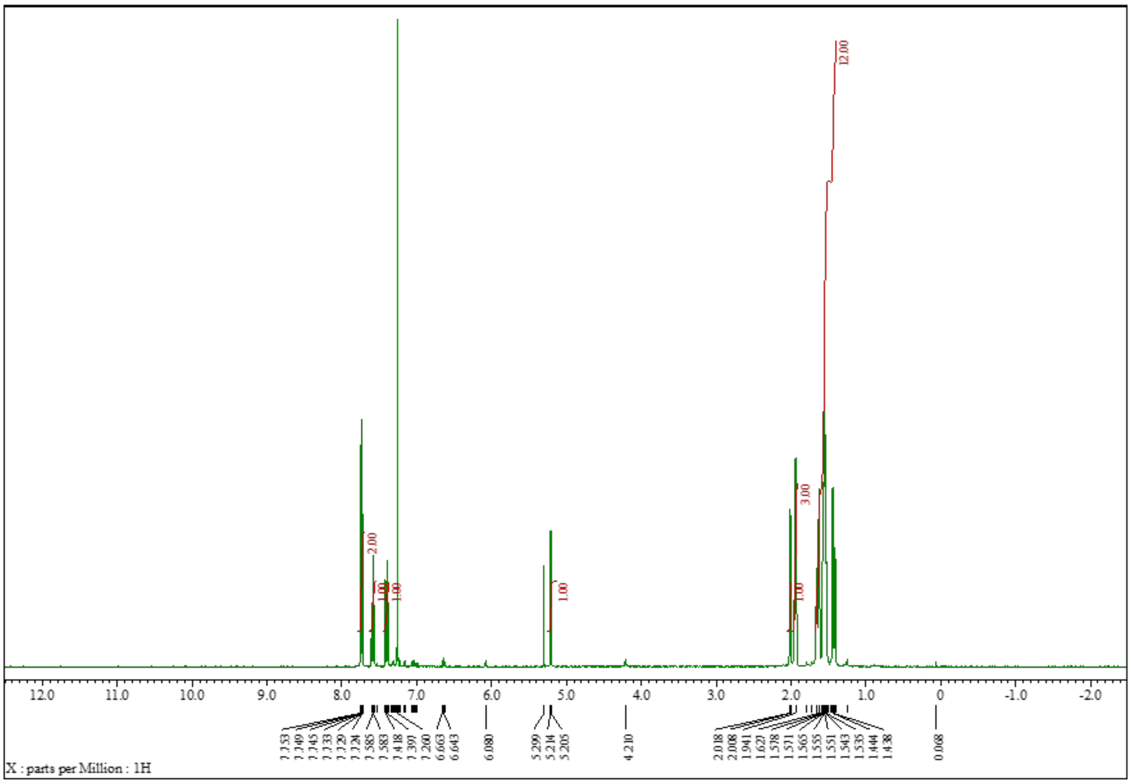

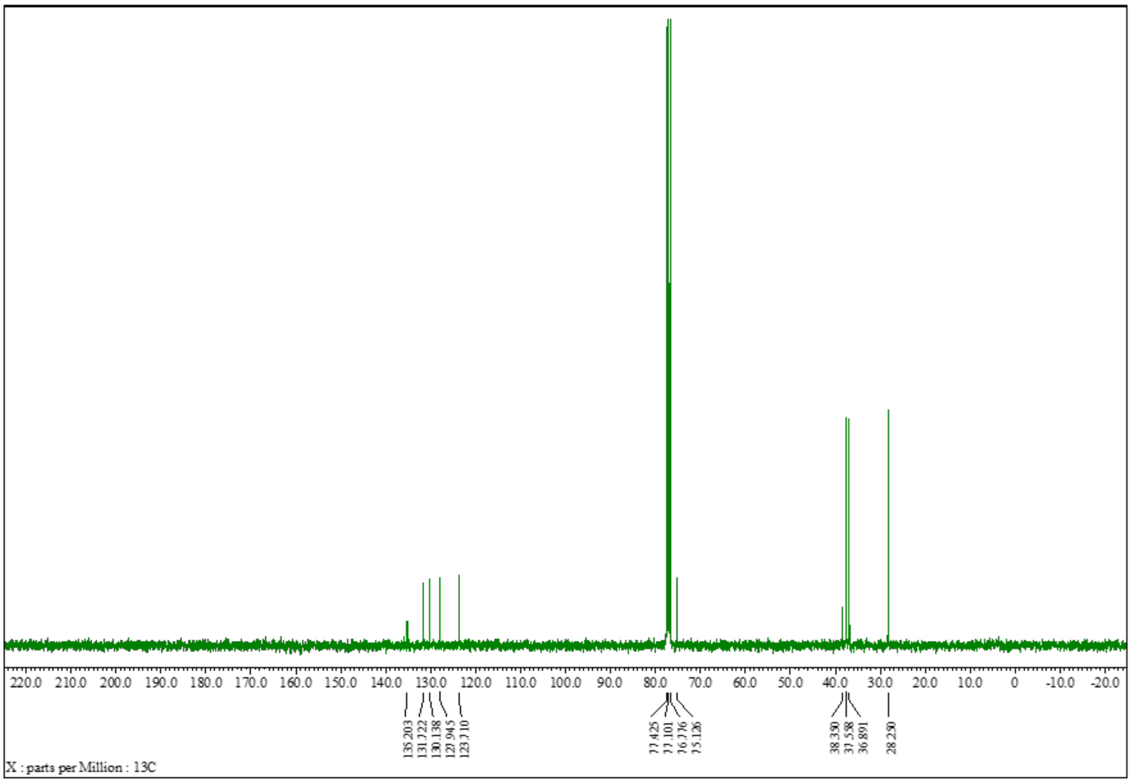

Compound 11

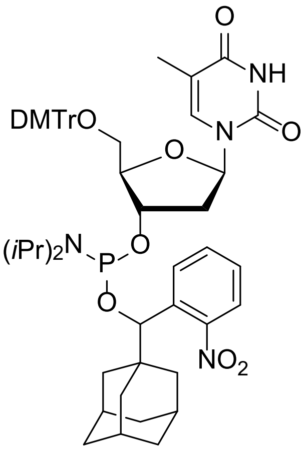

2'-deoxy-5'-*O*-(4,4'-dimethoxytrityl)-thymidine (258 mg, 0.47 mmol, 1.0 eq.) was dissolved in DCM (3 mL) and stirred at room temperature. To the solution, DIPEA (107 µL, 0.61 mmol, 1.3 eq.) and bis(diisopropylamino)chlorophosphine (163 mg, 0.61 mmol, 1.3 eq.) were added and stirred at room temperature for 2 hours. Then, Compound **10** (115 mg, 0.55 mmol, 1.0 eq.) and 1*H*-tetrazole (58.0 mg, 0.83 mmol, 1.5 eq.) were added to the reaction mixture and stirred at room temperature for 1.5 hours. After that, water was added to the reaction mixture and extracted by AcOEt. The organic layer was washed with saturated $NaHCO_3$ aqueous solution and brine, dried by Na_2SO_4, and concentrated. The

crude compound was purified by column silica gel column chromatography (DCM/AcOEt = 15/1→ 2/1) and Compound **11** (135 mg, 0.153 mmol, 28%) was obtained. ^{1}H-NMR (392 MHz, $CDCl_3$) (diastereomixture): δ 8.262 (br, 1H), 7.788-7.146 (m, 15H), 6.836-6.776 (m, 4H), 6.491-5.159 (m, 2H), 4.928-4.409 (m, 1H), 4.328-3.804 (m, 1H), 3.789-3.765 (m, 6H), 3.727-3.022 (m, 4H), 2.818-1.992 (m, 2H), 1.630-0.923 (m, 30H); ^{13}C-NMR (99 MHz, $CDCl_3$): δ 168.584, 158.760, 150.014, 149.089, 144.359, 135.889, 135.250, 131.579, 131.245, 130.186, 130.138, 128.231, 128.164, 128.050, 127.191, 123.853, 113.324, 111.159, 86.981, 85.570, 84.540, 77.310, 76.051, 63.624, 55.327, 43.376, 38.560, 37.692, 36.919, 36.738, 28.259, 24.912, 11.740; ^{31}P-NMR (159 MHz, $CDCl_3$): 153.108, 151.906, 150.169, 148.084; HRMS (ESI) calc. m/z 983.4331 (M + Na^+), found m/z 983.4336 (M + Na^+).

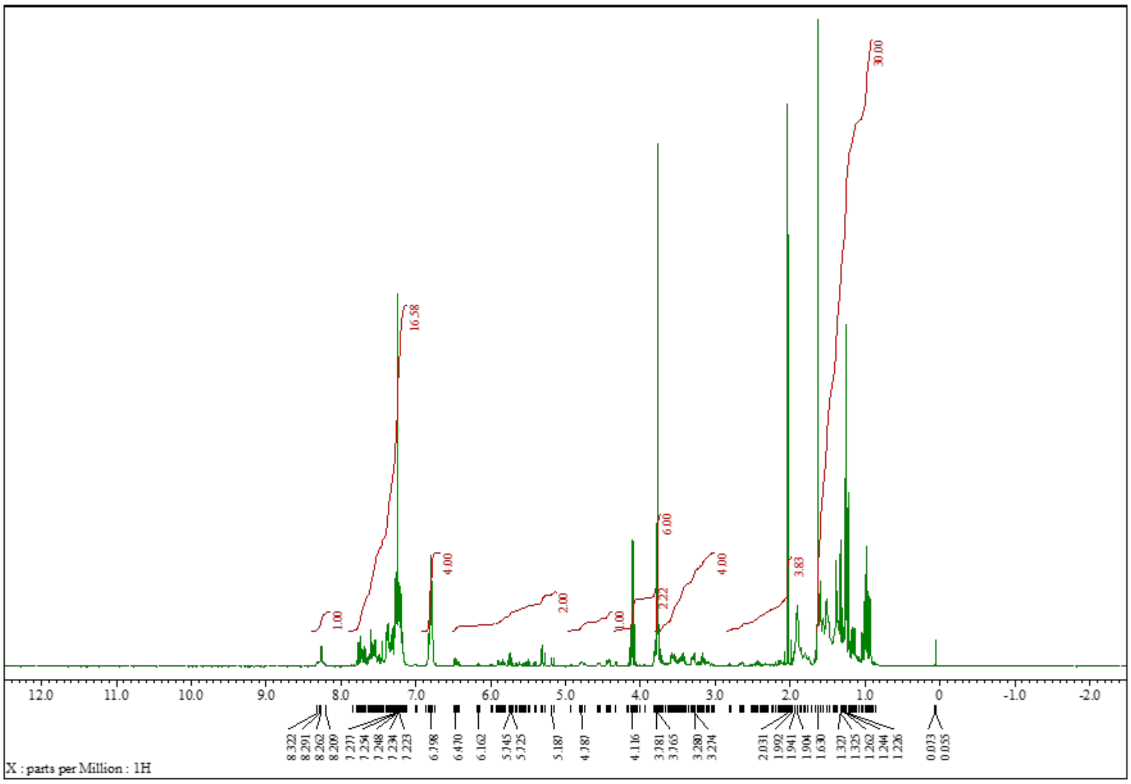

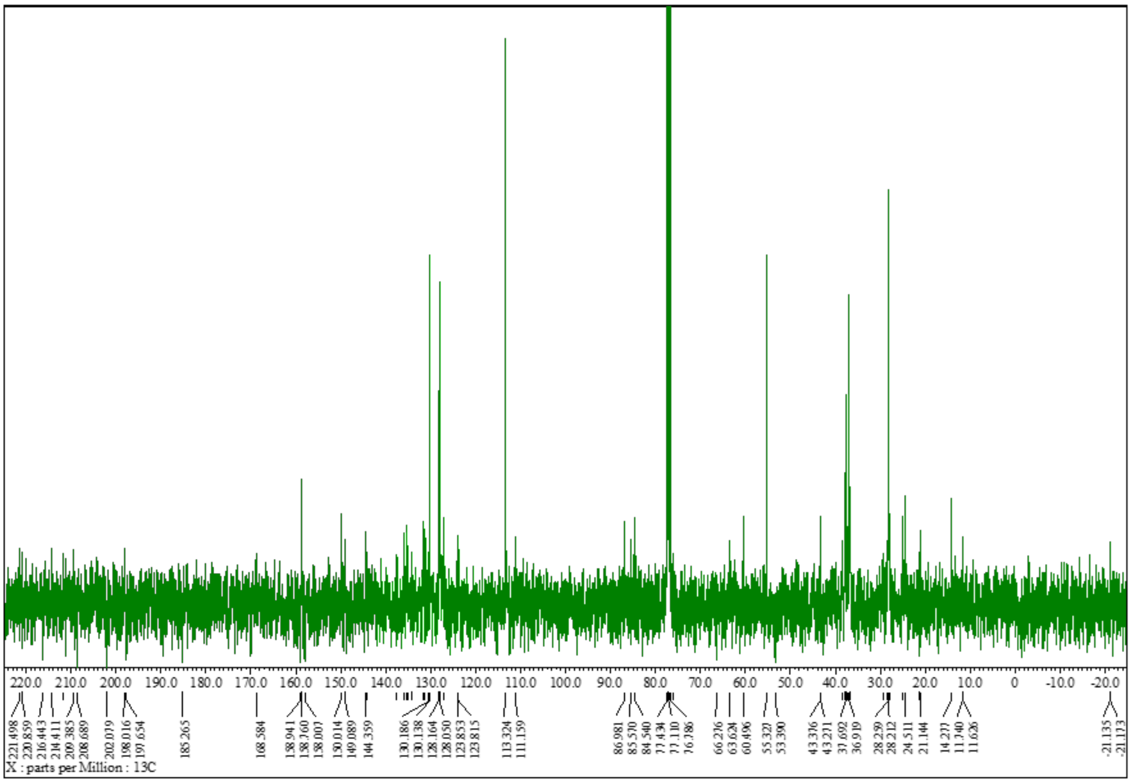
220.0 210.0 200.0 190.0 180.0 170.0 160.0 150.0 140.0 130.0 120.0 110.0 100.0 90.0 80.0 70.0 60.0 50.0 40.0 30.0 20.0 10.0 0 -10.0 -20.0
221.498 220.859 216.443 214.411 209.385 208.689 202.019 198.016 197.654 185.265 168.584 158.941 158.760 158.007 150.014 149.089 144.359 130.186 130.138 128.164 128.050 123.853 123.815 113.324 111.159 86.981 85.570 84.540 77.434 77.110 76.786 66.276 63.624 60.496 55.327 53.390 43.376 43.211 37.692 36.919 28.259 28.212 24.511 21.144 14.277 11.740 11.626 -21.135 -21.173
X : parts per Million : 13C

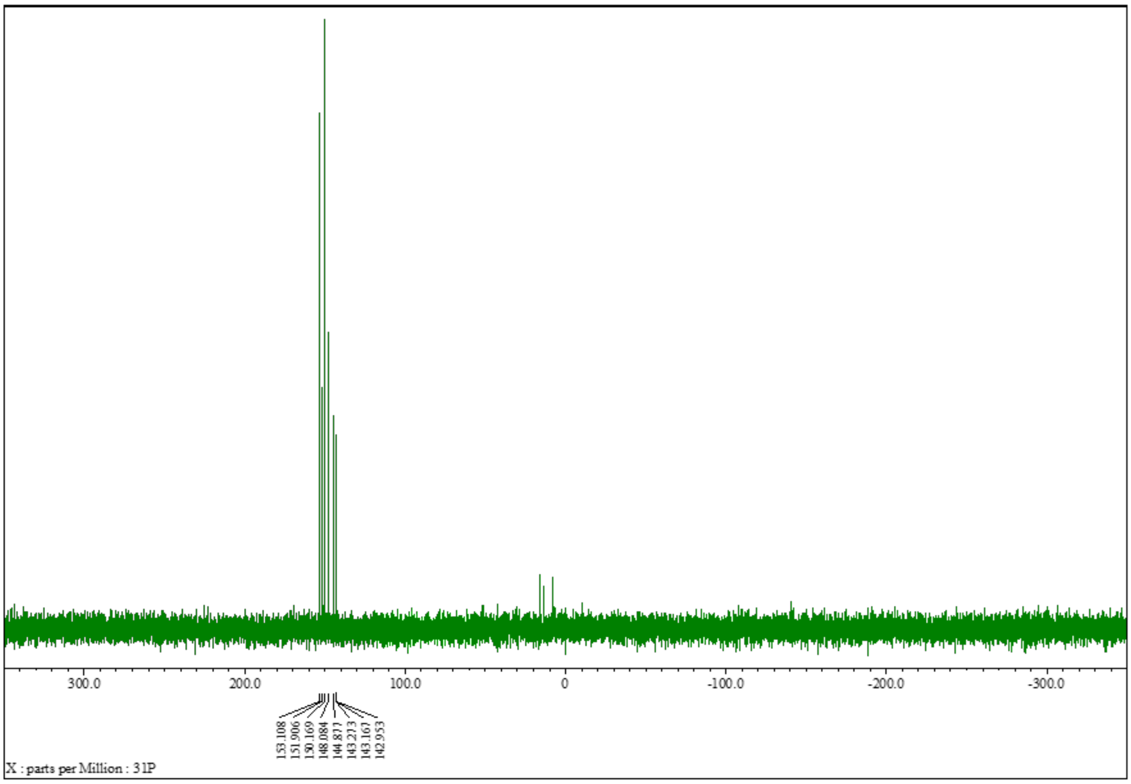
300.0 200.0 100.0 0 -100.0 -200.0 -300.0
153.108 151.906 150.169 148.084 144.877 143.273 143.167 142.953
X : parts per Million : 31P

Compound 12

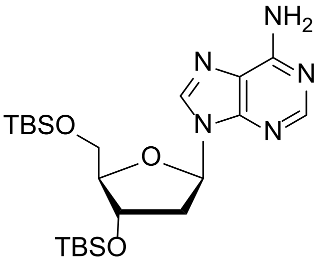

2'-deoxyadenosine (1.00 g, 3.98 mmol, 1.0 eq.) was dissolved in DMF (40 mL). Imidazole (1.63 g, 23.9 mmol, 6.0 eq.) and *tert*-butyldimethylchlorosilane (1.80 g, 11.9 mmol, 3.0 eq.) were added and stirred at room temperature. After stirring for 16 h, the reaction mixture was concentrated, diluted with AcOEt then washed with saturated $NaHCO_3$ aqueous solution and brine. The organic layer was dried with Na_2SO_4, filtered, and concentrated in vacuo. The crude product was purified by silica gel column chromatography (Hexane/AcOEt = 89/11→61:39→6:94) and Compound **12** (1.80 g, 3.76 mmol, 94%) was obtained. ^{1}H-NMR (392 MHz, $CDCl_3$): δ 8.337 (s, 1H), 8.144 (s, 1H), 6.448 (t, J = 6.6 Hz, 1H), 5.801 (br, 2H), 4.621-4.588 (m, 1H), 4.007 (q, J = 3.6 Hz, 1H), 3.871 (dd, J = 11.4, 4.2 Hz, 1H), 3.768 (dd, J = 11.6, 3.2 Hz, 1H), 0.909 (s, 9H), 0.904 (s, 9H), 0.093 (s, 6H), 0.084 (s, 6H); ^{13}C-NMR (99 MHz, $CDCl_3$): δ 155.451, 153.038, 149.623, 139.085, 119.991, 87.935, 84.387, 71.855, 62.794, 41.430, 26.037, 25.837, 18.512, 18.092, -4.731, -5.303; HRMS (ESI) calc. m/z 480.2821 (M + H$^+$), found m/z 480.2837 (M + H$^+$).

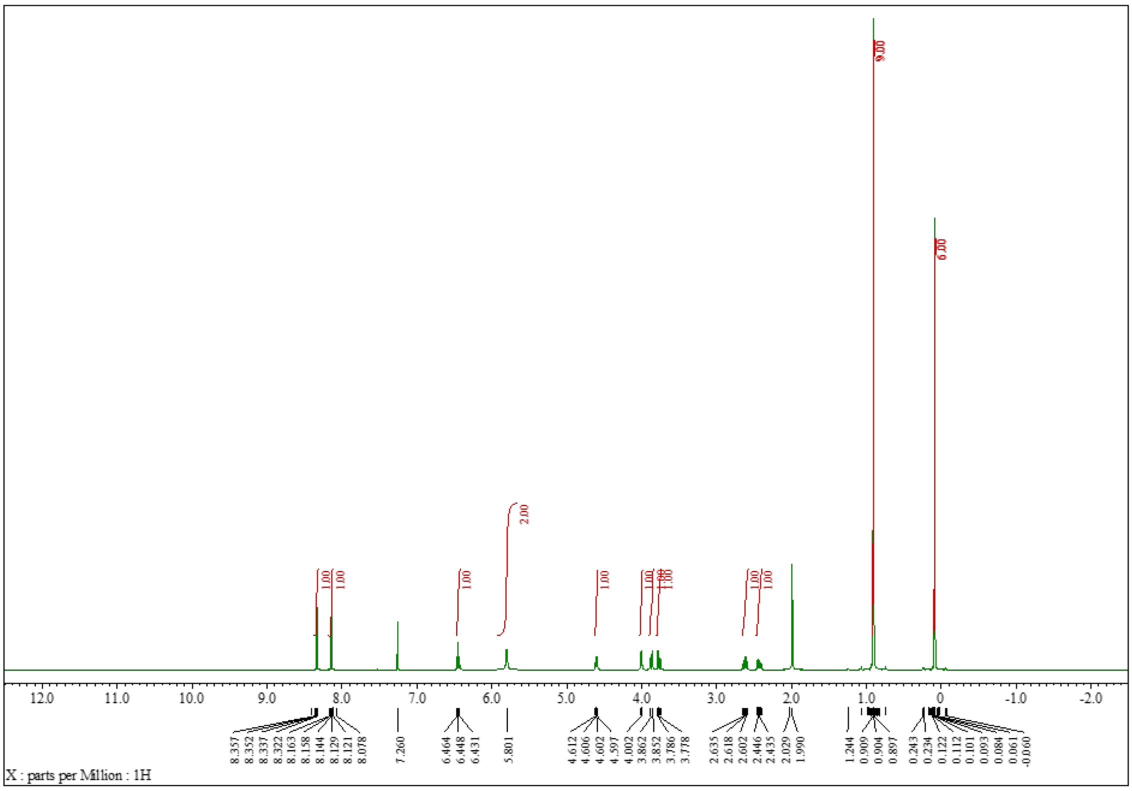

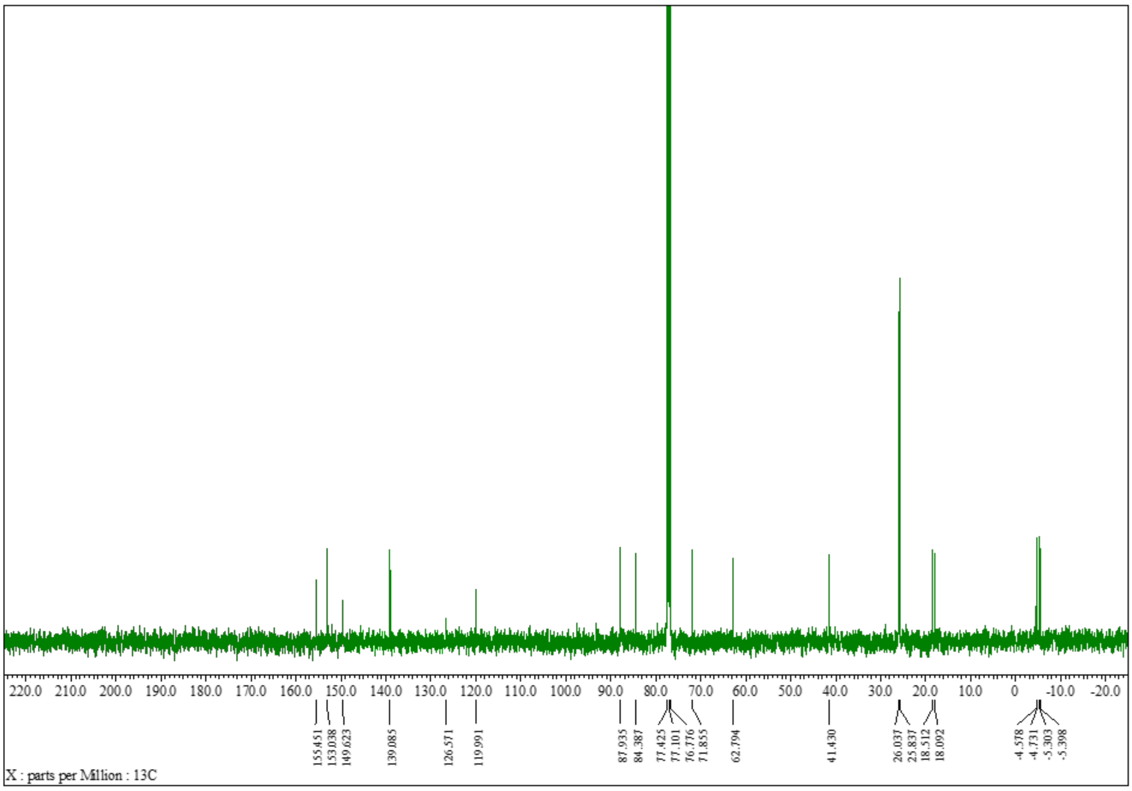

Compound 13

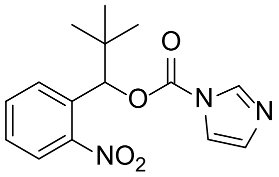

Compound **3** (1.00 g, 4.78 mmol, 1 eq.) was dissolved in dichloromethane (24 mL). 1,1-carbonyldiimidazole (1.94 g, 11.9 mmol, 2.5 eq.) was added and stirred at room temperature. After stirring for 20 h, the reaction mixture was diluted with dichloromethane and washed with saturated $NaHCO_3$ aqueous solution and brine. The organic layer was dried with Na_2SO_4, filtered, and concentrated in vacuo. The crude product was purified by silica gel column chromatography (Hexane/AcOEt = 85/15→57/43→35/65) and Compound **13** (1.41 g, 4.65 mmol, 97%) was obtained. ^{1}H-NMR (392 MHz, $CDCl_3$): δ 8.181 (s, 1H), 7.956 (dd, J = 8.4, 1.2 Hz, 1H), 7.638-7.554 (m, 2H), 7.516-7.473 (m, 1H), 7.449 (t, 1.4 Hz, 1H), 7.095 (q, J = 0.8 Hz, 1H), 6.693 (s, 1H), 1.040 (s, 9H); ^{13}C-NMR (99 MHz, $CDCl_3$): δ 149.281, 147.844, 136.980, 132.803, 131.787, 131.040, 129.354, 128.521, 125.053, 117.130, 80,485, 36.559, 25.915; HRMS (ESI) calc. m/z 304.1292 (M + H$^+$), found m/z 304.1293 (M + H$^+$).

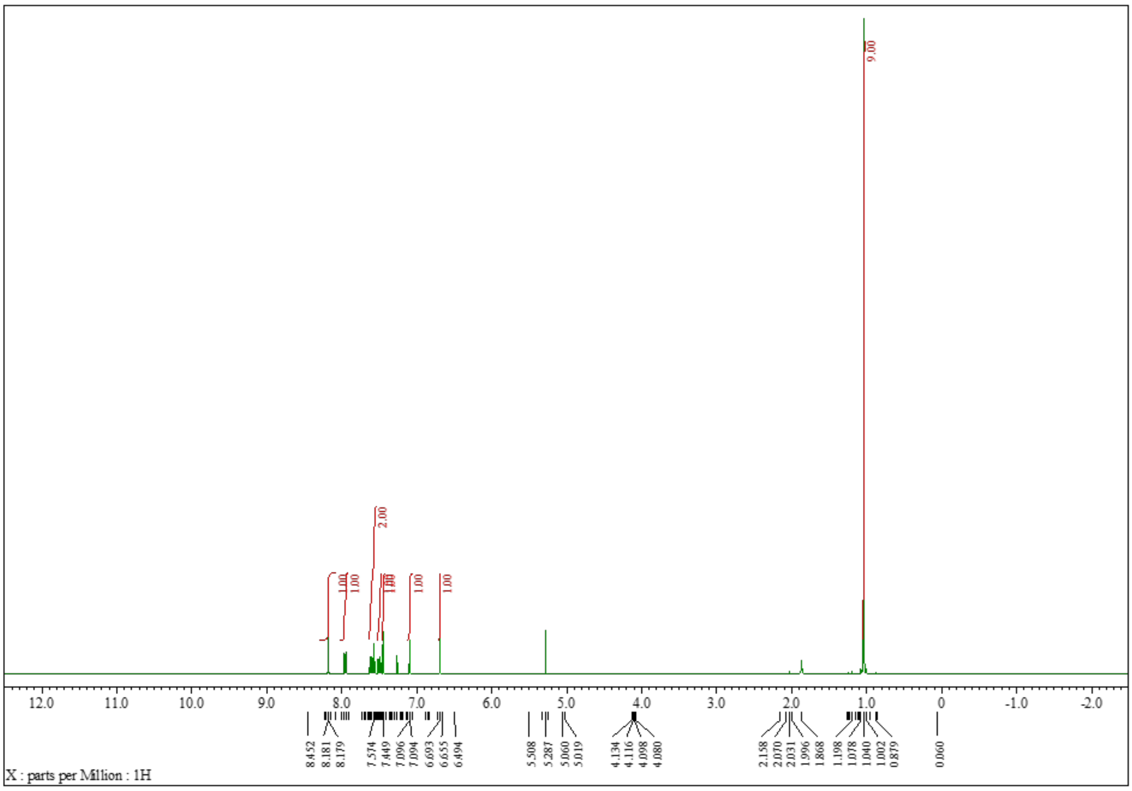
9.00
2.00
1.00
8.452
8.181
8.179
7.574
7.449
7.096
7.094
6.693
6.655
6.494
5.508
5.287
5.060
5.019
4.134
4.116
4.098
4.080
2.158
2.070
2.031
1.996
1.868
1.198
1.078
1.040
1.002
0.879
0.060
X : parts per Million : 1H

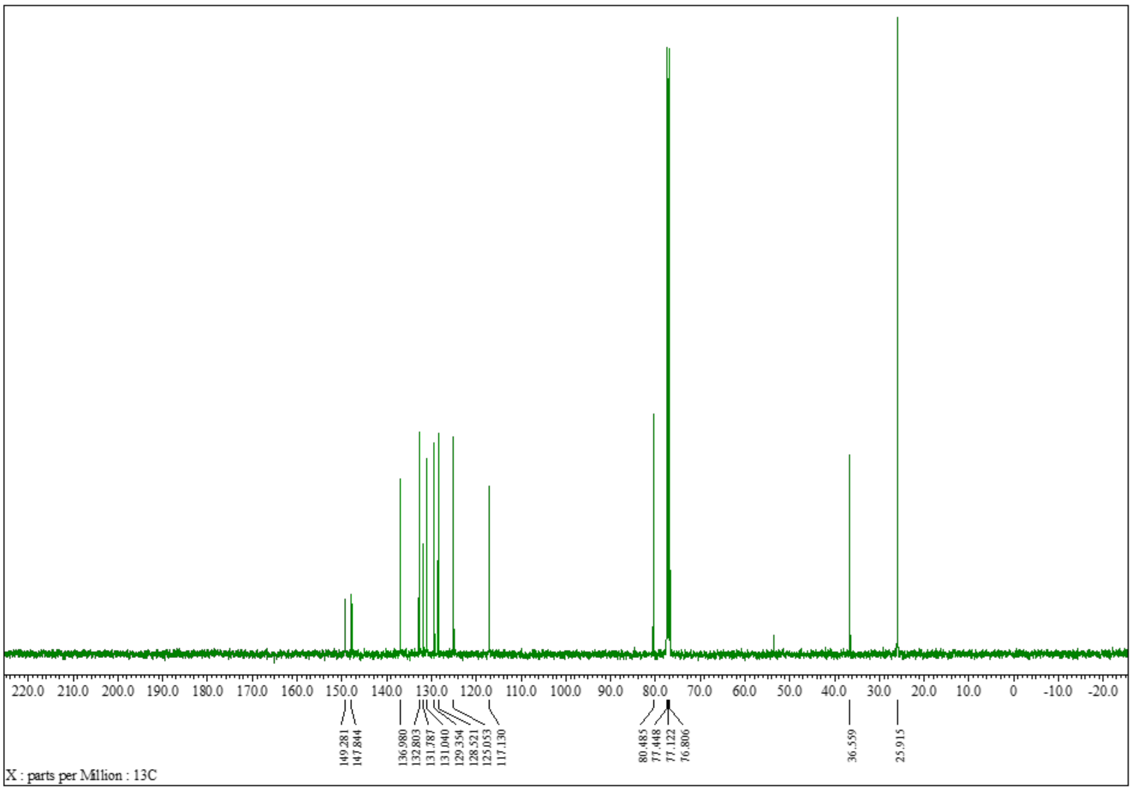
149.281
147.844
136.980
132.803
131.787
131.040
129.354
128.521
125.053
117.130
80.485
77.448
77.122
76.806
36.559
25.915
X : parts per Million : 13C

Compound 15

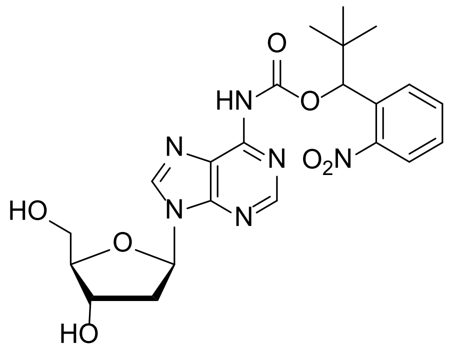

Compound **13** (854 mg, 2.81 mmol, 1.5 eq.) was dissolved in dichloromethane (18 mL). Triethyloxonium tetrafluoroborate (535 mg, 2.81 mmol, 1.5 eq.) was added and stirred at room temperature. After stirring for 2 h, Compound **12** (900 mg, 1.88 mmol, 1 eq.) was added then stirred at room temperature. After stirring for 21 h, the reaction mixture was diluted with AcOEt then washed with saturated $NaHCO_3$ aqueous solution and brine. The organic layer was dried with Na_2SO_4, filtered, and concentrated in vacuo. The crude product was dissolved in THF (18 mL) and added triethylamine trihydrofluoride (1.53 mL, 9.39 mmol, 5.0 eq.). After stirring for 16 h at room temperature, the reaction mixture was concentrated and purified by silica gel column chromatography (DCM/MeOH = 100/0→98/2→95/5) and Compound **15** (765 mg, 1.57 mmol, 84%) was obtained. ^{1}H-NMR (392 MHz, DMSO-d_6): δ 10.906 (s, 1H), 8.671 (s, 1H), 8.600 (s, 1H), 7.974 (d, J = 8.4 Hz, 1H), 7.841-7.762 (m, 2H), 7.615-7.572 (m, 1H), 6.441 (t, J = 6.8 Hz, 1H), 6.251 (s, 1H), 5.341 (d, J = 4.0 Hz, 1H), 5.002 (t, J = 5.8 Hz, 1H), 4.452-4.411 (m, 1H), 3.887 (dd, J = 7.6, 4.4 Hz, 1H), 3.649-3.593 (m, 1H), 3.552-3.495(m, 1H), 2.785-2.717 (m, 1H), 2.363-2.304 (m, 1H), 0.926 (s, 9H); ^{13}C-NMR (99 MHz, DMSO-d_6): δ 152.103, 151.817, 151.655, 149.976, 149.442, 143.195, 133.457, 133.066, 129.766, 129.652, 124.883, 123.710, 88.526, 84.292, 76.776, 71.187, 62.117, 36.414, 25.999; HRMS (ESI) calc. m/z 487.1936 (M + H^+), found m/z 487.1938 (M + H^+).

※1 peak was same position of DMSO's peak (around 40 ppm), so 1 carbon peak was missing.

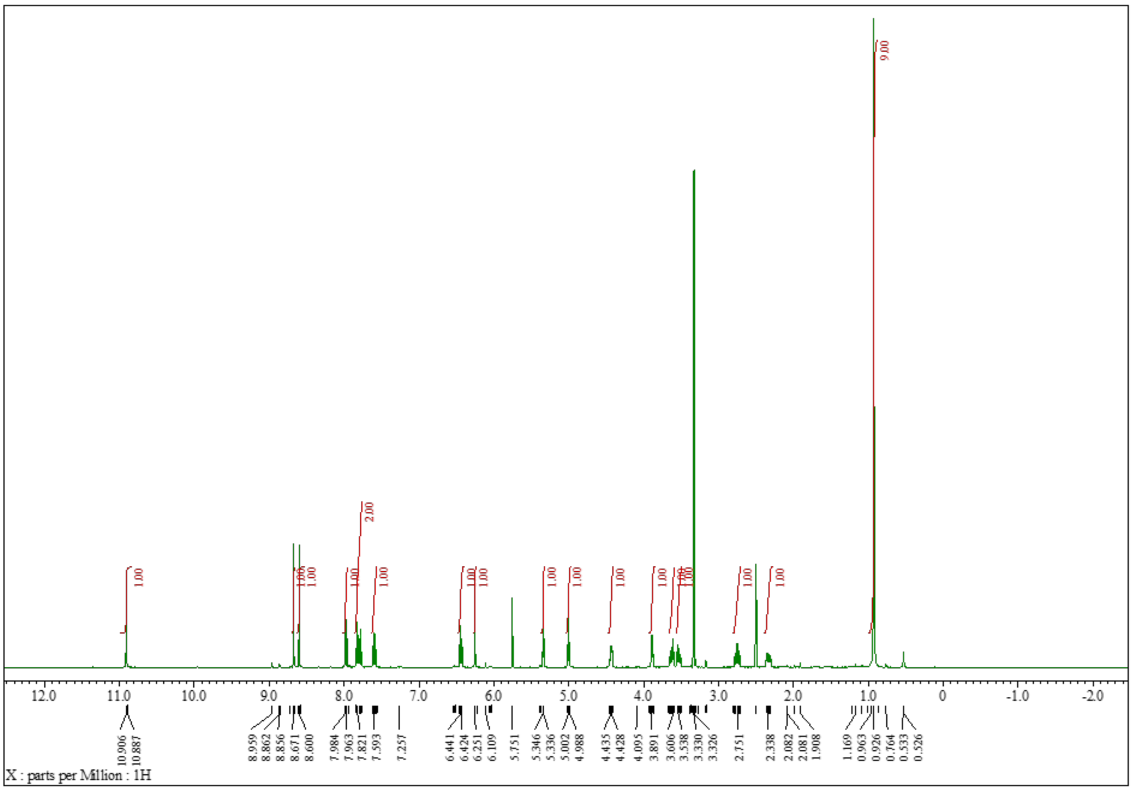

X : parts per Million : 1H
10.906 10.887 8.959 8.862 8.856 8.671 8.600 7.984 7.963 7.821 7.593 7.257 6.441 6.424 6.251 6.109 5.751 5.346 5.336 5.002 4.988 4.435 4.428 4.095 3.891 3.606 3.538 3.330 3.326 2.751 2.338 2.082 2.081 1.908 1.169 0.963 0.926 0.764 0.533 0.526

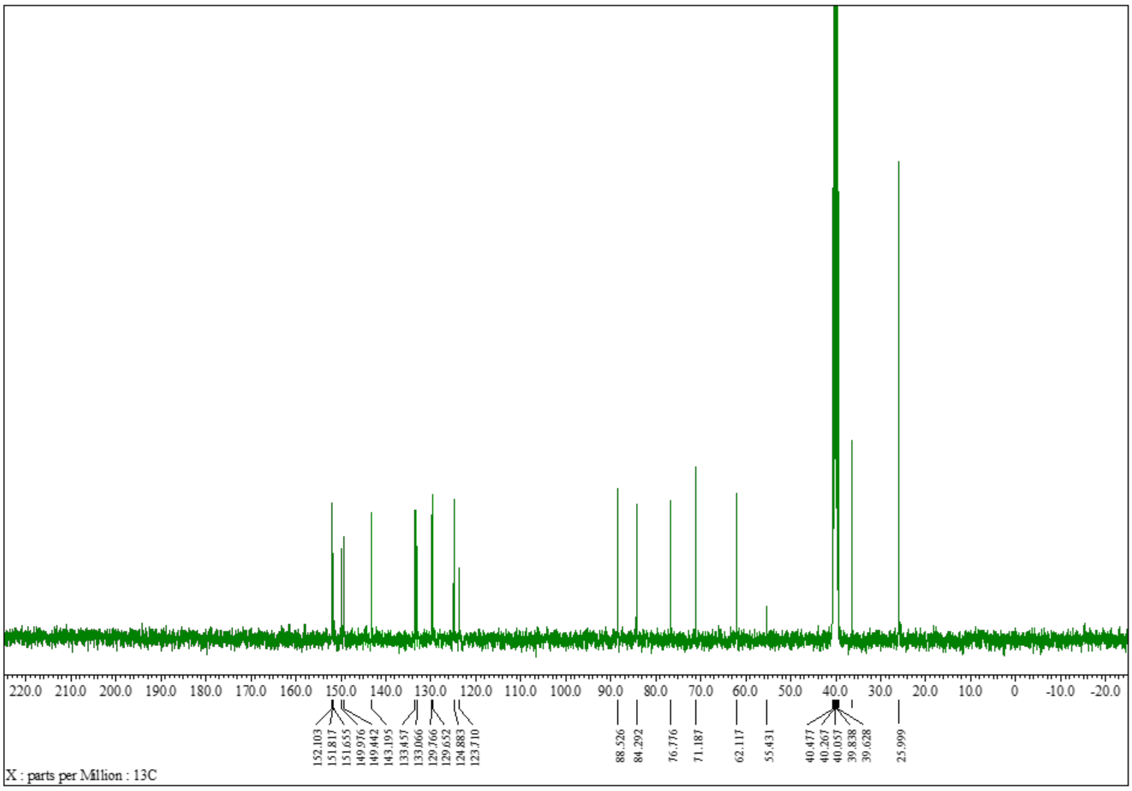

X : parts per Million : 13C
152.103 151.817 151.655 149.976 149.442 143.195 133.457 133.066 129.766 129.652 124.883 123.710 88.526 84.292 76.776 71.187 62.117 55.431 40.477 40.267 40.057 39.838 39.628 25.999

Compound 16

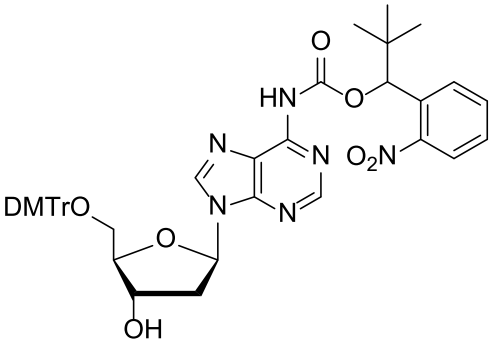

Compound **15** (260 mg, 0.534 mmol, 1.0 eq.) was dissolved in pyridine (1.3 mL). 4-dimethylaminopyridine (1.31 mg, 0.0107 mmol, 0.020 eq.) and 4,4'-dimethoxytrityl chloride (199 mg, 0.588 mmol, 1.1 eq.) were added and stirred at room temperature. After stirring for 19 h, the reaction mixture was diluted with AcOEt then washed with saturated $NaHCO_3$ aqueous solution and brine. The organic layer was dried with Na_2SO_4, filtered, and concentrated in vacuo. The crude product was purified by silica gel column chromatography (AcOEt/MeOH = 100/0→98/2) and Compound **16** (339 mg, 0.430 mmol, 81%) was obtained. ^{1}H-NMR (392 MHz, $CDCl_3$): δ 8.635 (s, 1H), 8.511 (d, *J* = 4.0 Hz, 1H), 8.130 (d, *J* = 2.4 Hz, 1H), 7.922 (d, *J* = 8.4 Hz, 1H), 7.675-7.641 (m, 1H), 7.578-7.535 (m, 1H), 7.432-7.407 (m, 1H), 7.388-7.360 (m, 2H), 7.305-7.175 (m, 7H), 6.824-6.774 (m, 4H), 6.632 (2s, 1H), 6.466 (t, *J* = 6.6 Hz, 1H), 4.724-4.689 (m, 1H), 4.152 (q, *J* = 4.4 Hz, 1H), 3.768 (2s, 6H), 3.413 (d, *J* = 4.0 Hz, 2H), 2.899-2.822 (m, 1H), 2.599-2.539 (m, 1H), 1.004 (s, 9H); ^{13}C-NMR (99 MHz, $CDCl_3$): δ 158.684, 152.838, 150.692, 150.129, 149.251, 144.473, 141.259, 135.622, 133.476, 132.485, 130.072, 129.156, 128.584, 128.164, 128.002, 127.096, 124.807, 123.853, 122.232, 113.286, 86.781, 86.314, 84.769, 78.045, 72.513, 63.634, 55.317, 40.381, 36.309, 25.961; HRMS (ESI) calc. *m*/*z* 789.3243 (M + H^+), found *m*/*z* 789.3251 (M + H^+).

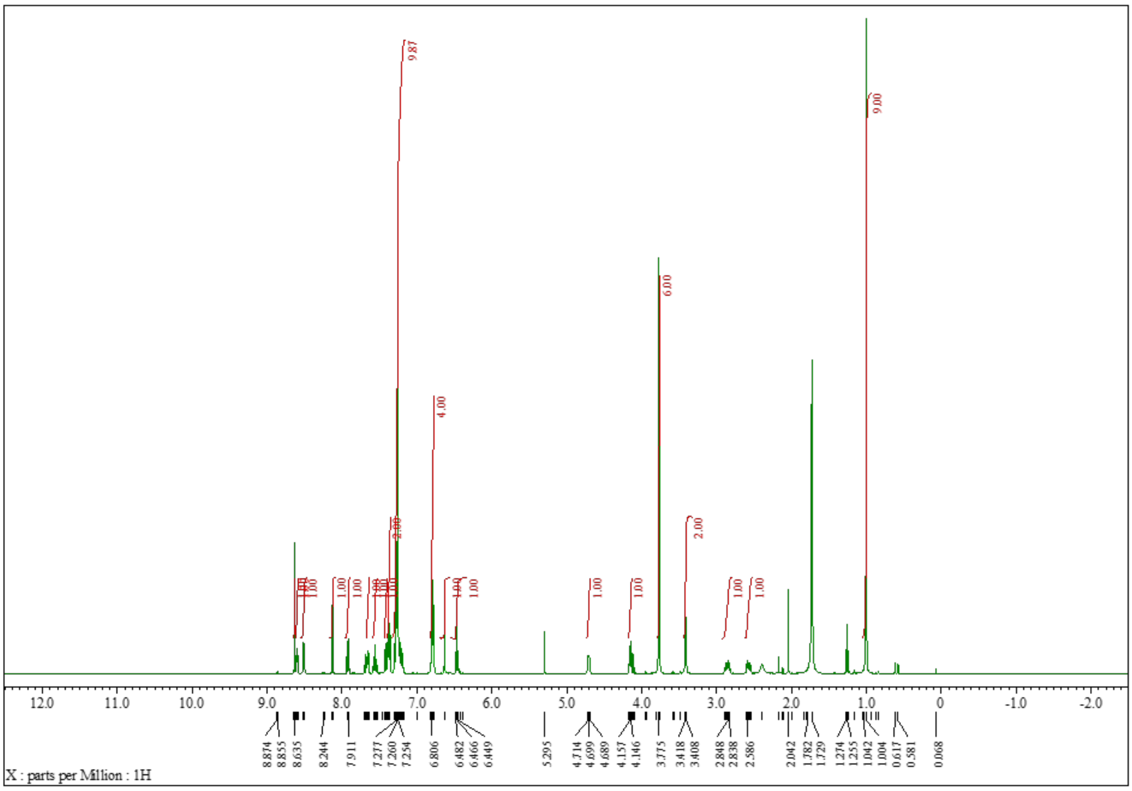

X : parts per Million : 1H

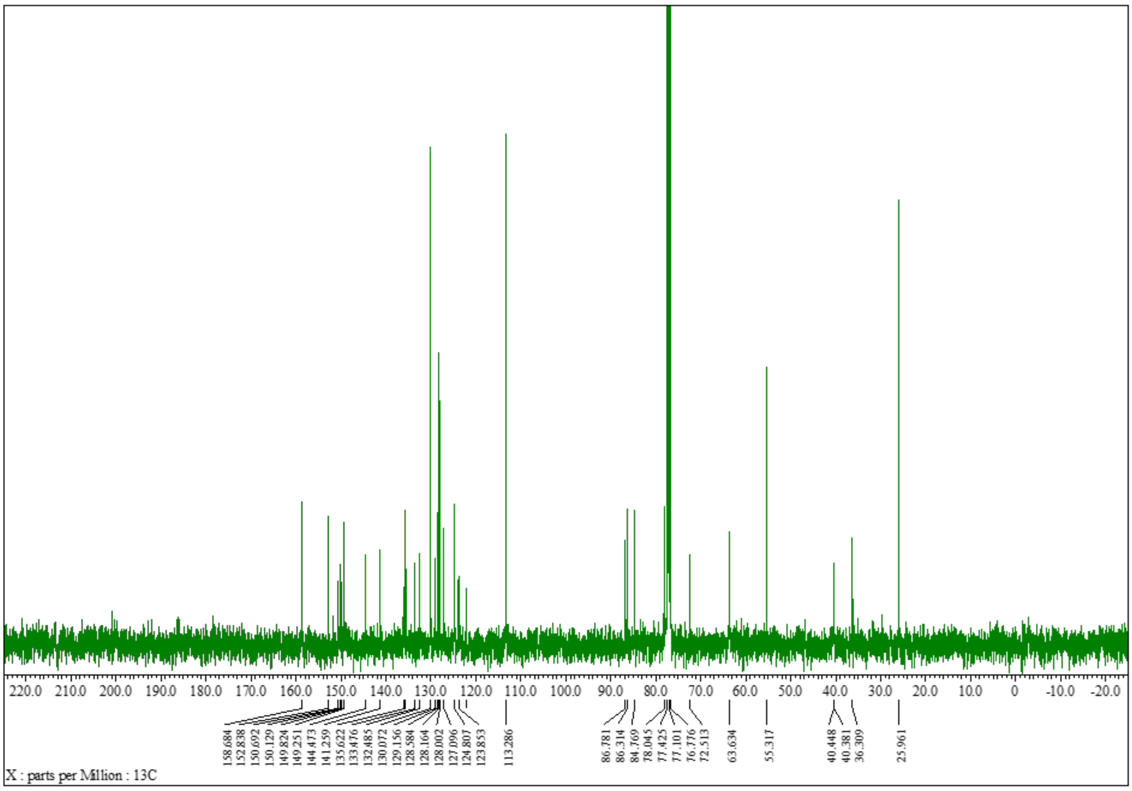

X : parts per Million : 13C

Compound 17

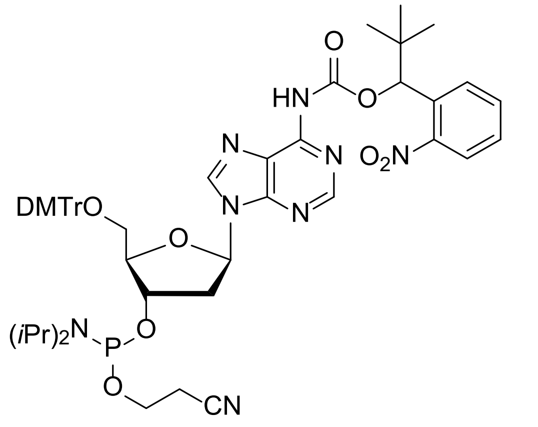

Compound **16** (300 mg, 0.380 mmol, 1.0 eq.) was dissolved in DCM (1.0 mL). 2-cyanoethyl *N*,*N*-diisopropylchlorophosphoramidite (117 mg, 0.494 mmol, 1.3 eq.) and DIPEA (165 μL, 0.947 mmol, 2.5 eq.) were added and stirred at room temperature. After stirring overnight, the reaction mixture was diluted with DCM and washed with saturated $NaHCO_3$ aqueous solution and brine. The organic layer was dried with Na_2SO_4, filtered, and concentrated in vacuo. The crude product was dissolved in small amount of AcOEt then Hexane was added to the solution. The precipitation was filtered and collected, and Compound **16** (310 mg, 0.313 mmol, 82%) was obtained. ^{1}H-NMR (392 MHz, $CDCl_3$) (diastereomixture): δ 8.647 (s, 1H), 8.413 (br, 1H), 8.194-8.163 (m, 1H), 7.928 (d, J = 8.0 Hz, 1H), 7.661-7.525 (m, 2H), 7.435-7.171 (m, 10 H), 6.798-6.756 (m, 4H), 6.636-6.632 (m, 1H), 6.495-6.454 (m, 1H), 4.808-4.774 (m, 1H), 4.321-4.282 (m, 1H), 3.895-3.558 (m, 10H), 3.489-3.309 (m, 2H), 2.945-2.880 (m, 1H), 2.773-2.695 (m, 1H), 2.616 (t, J = 6.4 Hz, 1H), 2.465 (t, J = 6.4 Hz, 1H), 1.195-1.105 (m, 12H), 1.000 (s, 9H) ; ^{13}C-NMR (99 MHz, $CDCl_3$): δ 158.617, 152.847, 150.825, 150.062, 149.213, 144.502, 141.412, 135.651, 135.505, 132.513, 130.110, 129.127, 128.584, 128.240, 128.193, 127.935, 127.039, 124.826, 122.251, 117.654, 113.209, 86.609, 86.123, 84.826, 78.016, 73.543, 63.386, 58.283, 55.317, 43.395, 39.714, 36.280, 25.961, 24.654, 20.486; ^{31}P-NMR (159 MHz, $CDCl_3$): δ 149.474, 149.367; HRMS (ESI) calc. *m*/*z* 989.4321 (M + H$^+$), found *m*/*z* 989.4312 (M + Na$^+$).

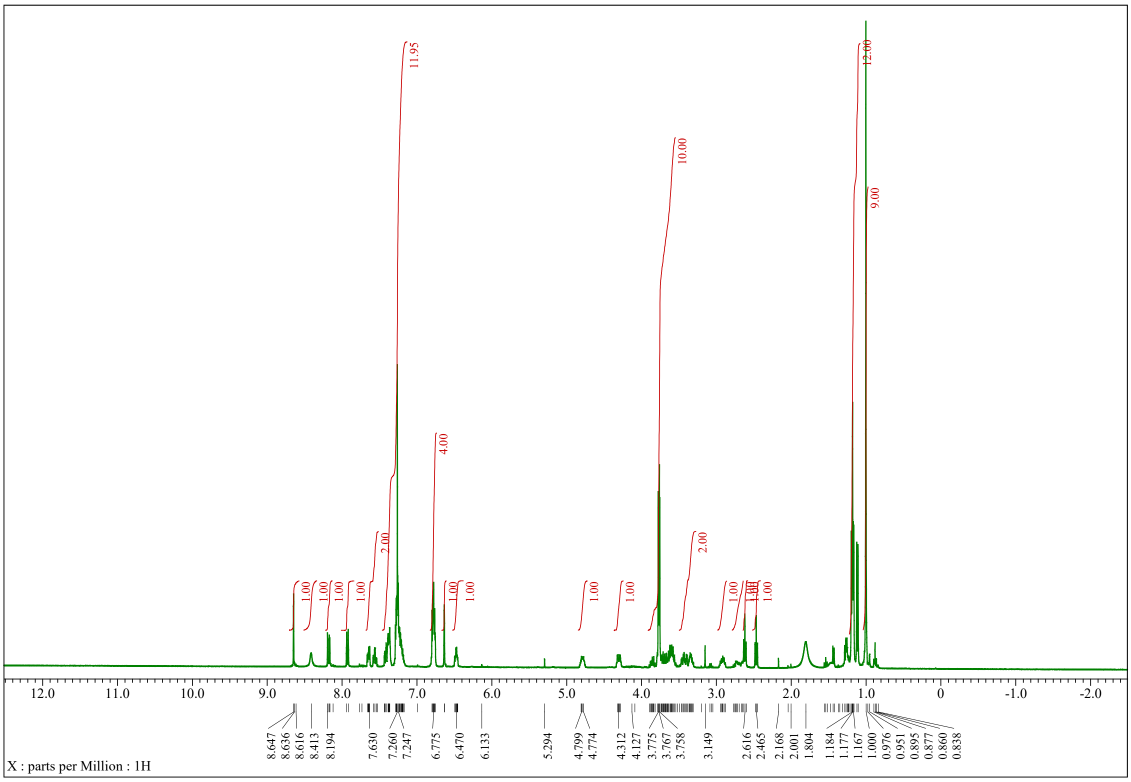

X : parts per Million : 1H

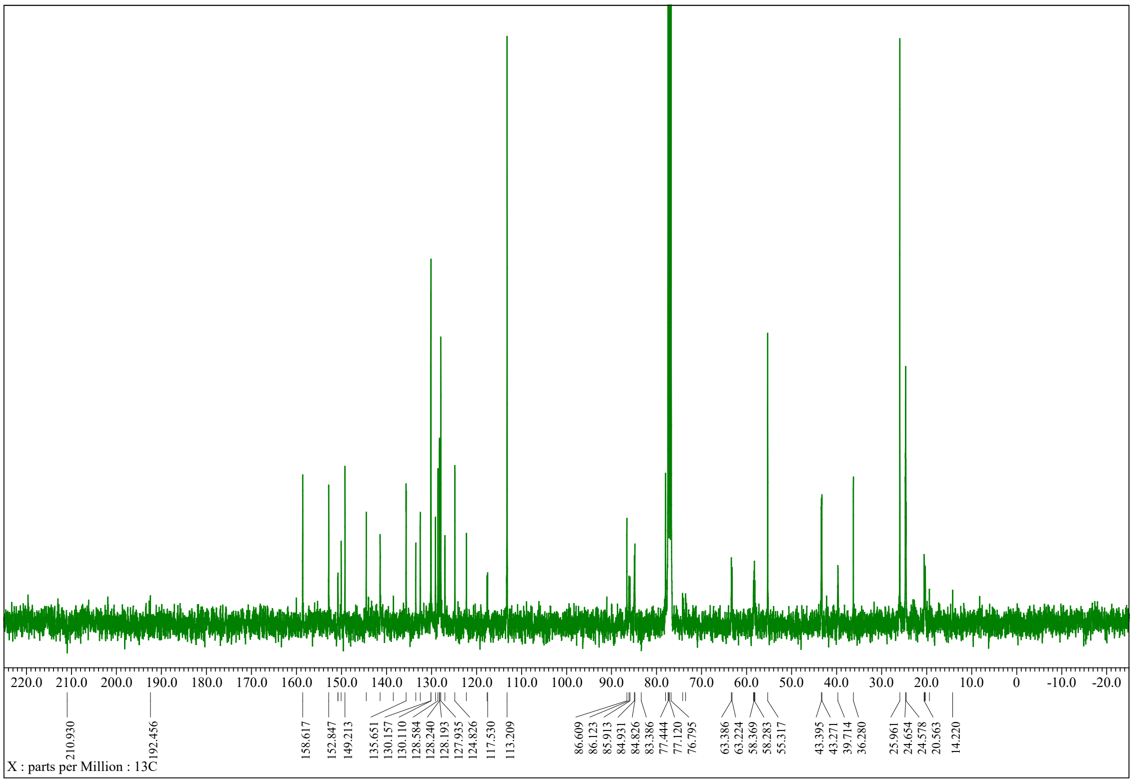

X : parts per Million : 13C

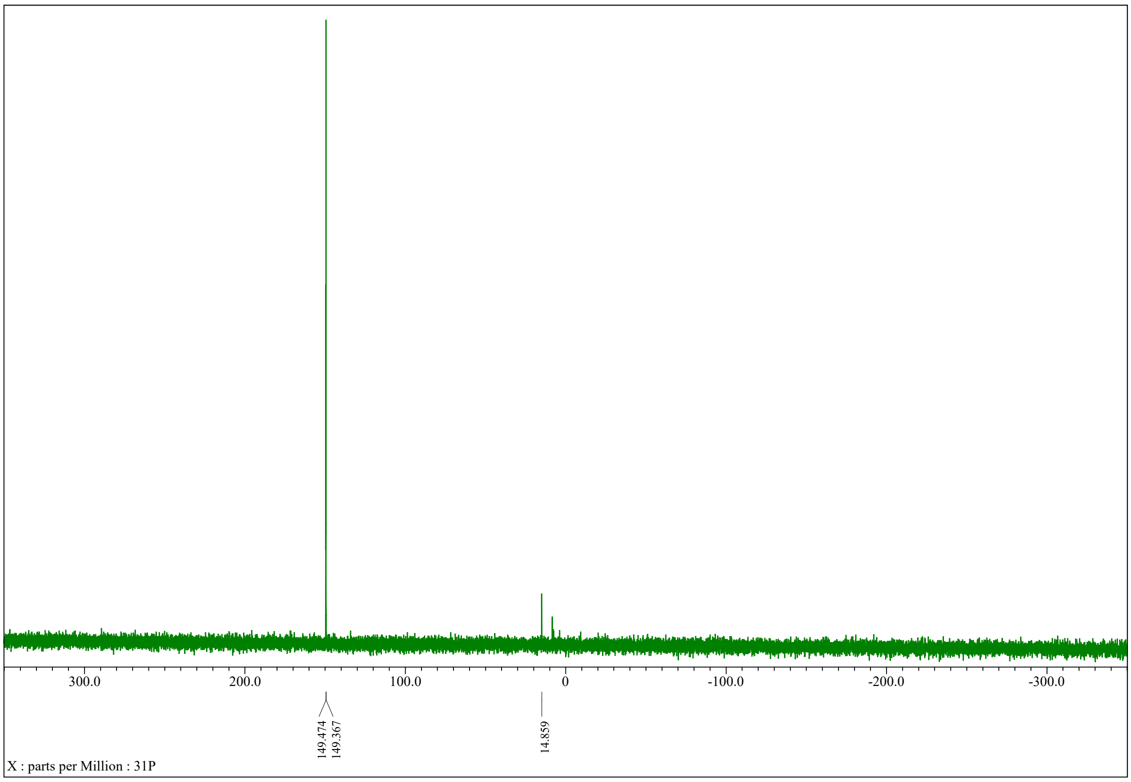

Compound 18

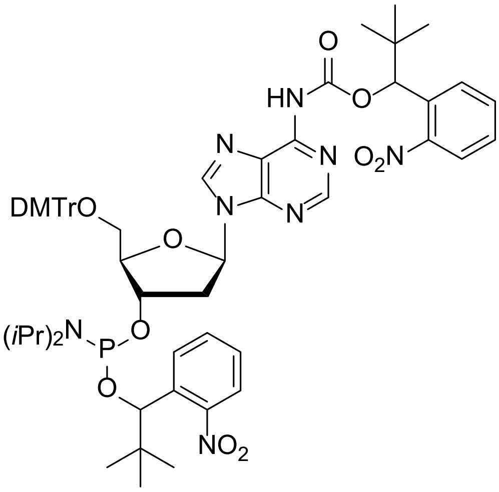

Compound **16** (300 mg, 0.380 mmol, 1.0 eq.) was co-evaporated with benzene and dissolved in DCM (0.9 mL). Compound **4** (251 mg, 0.571 mmol, 1.5 eq.) and 5-(methylthio)-1*H*-tetrazole (133 mg, 1.15 mmol, 3.0 eq.) were added at 0 °C and stirred at room temperature. After stirring for 14 h, the reaction mixture was diluted with AcOEt then washed with saturated $NaHCO_3$ aqueous solution and brine. The organic layer was dried with Na_2SO_4, filtered, and concentrated in vacuo. The crude product was purified by silica gel column chromatography (Hexane/AcOEt = 87/13→75/25→0/100 with 1% TEA)

and Compound **18** (147 mg, 0.130 mmol, 34%) was obtained. ^{1}H-NMR (392 MHz, DMSO-d_6) (diastereomixture): δ 10.922-10.895 (m, 1H), 8.622-8.425 (m, 2H), 7.987-7.966 (m, 1H), 7.884-7.447 (m, 7H), 7.305-7.097 (m, 9H), 6.806-6.677 (m, 4H), 6.537-6.310, 6.096-5.924 (m, 1H), 6.255 (s, 1H), 5.543-5.196 (m, 1H), 5.135-4.505 (m, 1H), 4.151-3.762 (m, 1H), 3.704-3.659 (m, 6H), 3.640-3.421 (m, 2H), 3.230-2.641 (m, 3H), 2.557-2.527, 2.367-2.160, 2.029-1.955 (m, 1H), 1.229-0.760 (m 30H); ^{13}C-NMR (99 MHz, DMSO-d_6): δ 158.531, 151.989, 151.559, 150.033, 149.528, 145.265, 143.739, 135.909, 133.429, 133.066, 132.780, 130.930, 130.463, 130.072, 129.766, 129.652, 129.471 128.956, 128.212, 128.097, 127.134, 124.883, 124.349, 123.834, 123.710, 113.543, 86.066, 85.207, 84.349, 76.757, 75.288, 72.923, 63.872, 55.479, 49,747, 43.214, 36.881, 36.404, 25.980, 25.846, 24.740; ^{31}P-NMR (159 MHz, DMSO-d_6): δ 151.879, 150.516, 148.405, 147.416, 141.162, 140.948, 140.601, 140.066; HRMS (ESI) calc. *m*/*z* 1127.5002 (M + H^{+}), found *m*/*z* 1127.5007 (M + H^{+}).

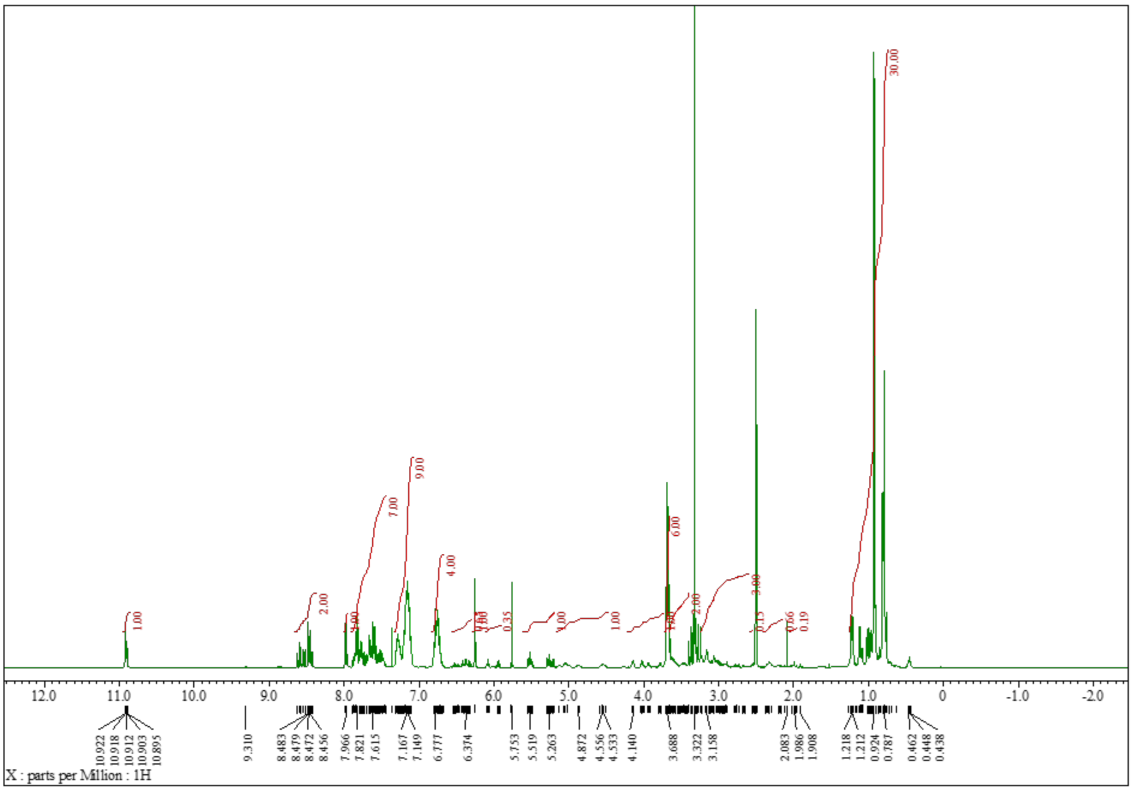

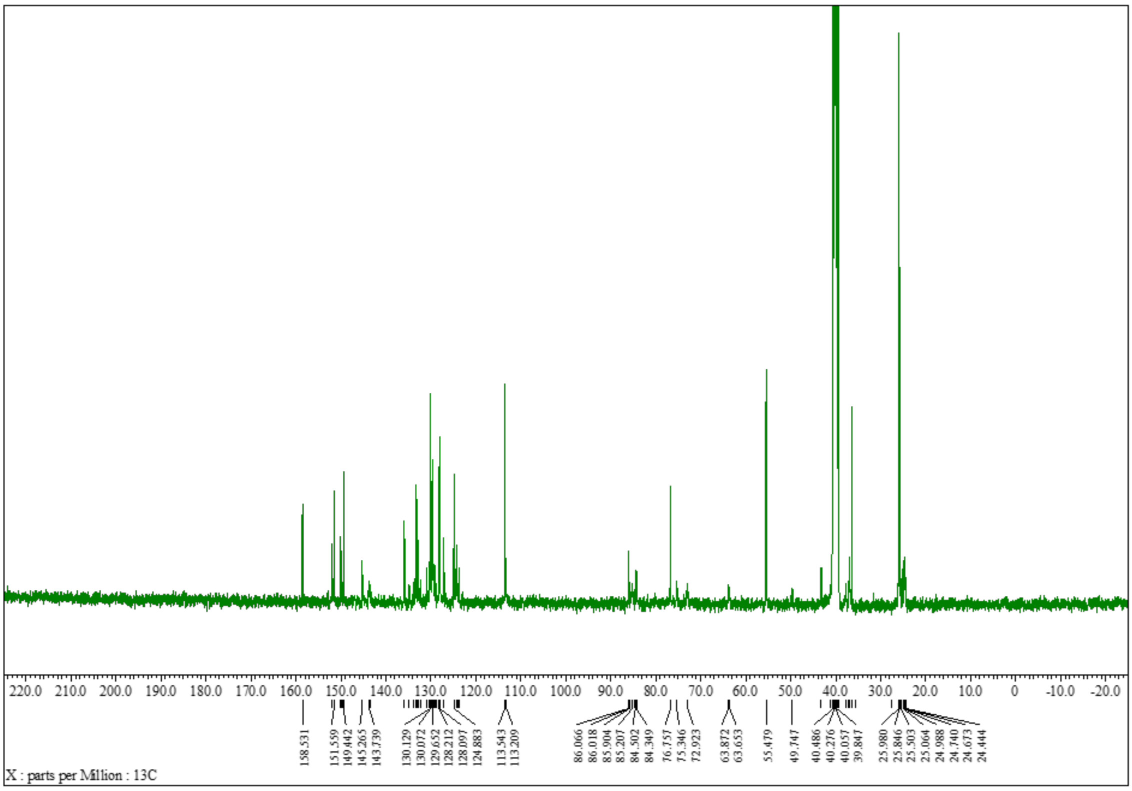
158.531
151.559
149.442
145.265
143.739
130.129
130.072
129.652
128.212
128.097
124.883
113.543
113.209
86.066
86.018
85.904
85.207
84.502
84.349
76.757
75.346
72.923
63.872
63.653
55.479
49.747
40.486
40.276
40.057
39.847
25.980
25.846
25.503
25.064
24.988
24.740
24.673
24.444
X : parts per Million : 13C

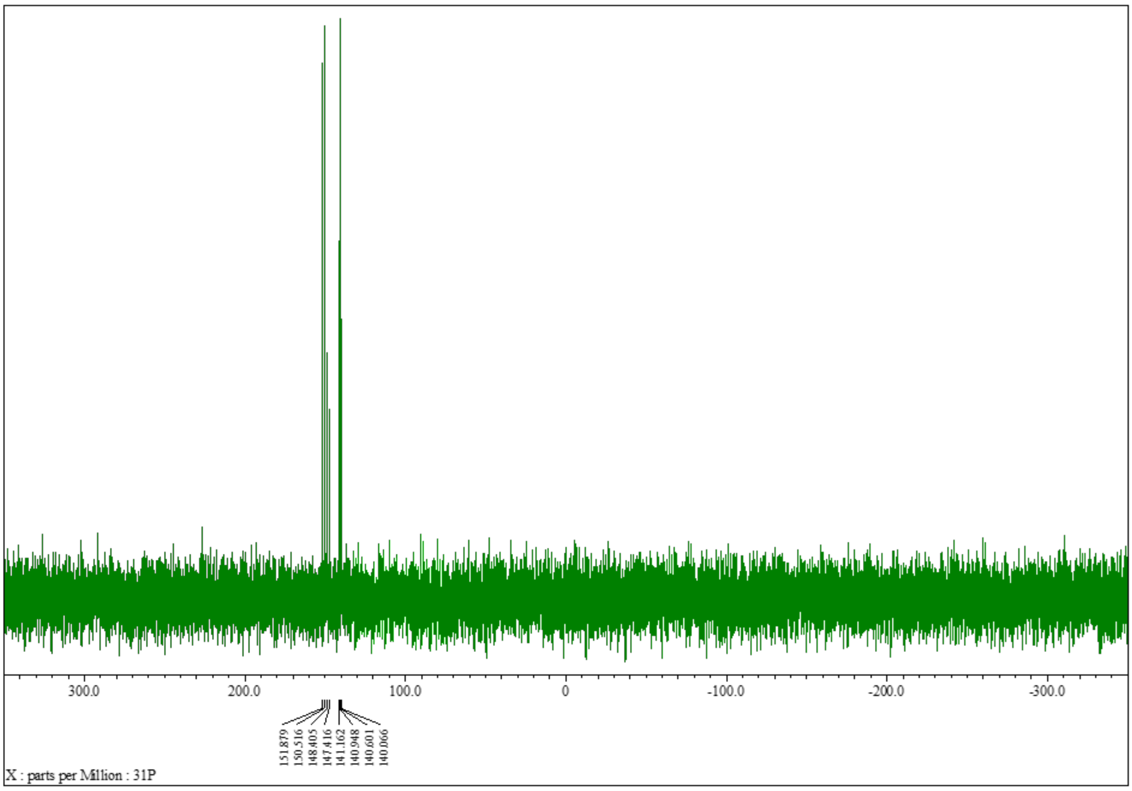
151.879
150.516
148.405
147.416
141.162
140.948
140.601
140.066
X : parts per Million : 31P

Compound 19

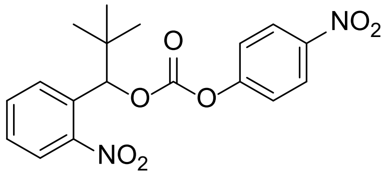

Compound **3** (100 mg, 0.478 mmol, 1 eq.) was dissolved in dichloromethane (5.00 mL). DMAP (175 mg, 1.43 mmol, 3 eq.) and 4-nitrophenyl chloroformate (231 mg, 1.15 mmol, 2.4 eq.) were added and stirred at room temperature. After stirring for 24 h, the reaction mixture was diluted with AcOEt then washed with saturated NH_4Cl aqueous solution, saturated $NaHCO_3$ aqueous solution, followed by brine. The organic layer was dried with Na_2SO_4, filtered, and concentrated in vacuo. The crude product was purified by silica gel column chromatography (Hexane/DCM = 73/27→57/43→0/100) and Compound **19** (177 mg, 0.474 mmol, 99%) was obtained. ^{1}H-NMR (392 MHz, $CDCl_3$): δ 8.257-8.218 (m, 2H), 7.939 (dd, J = 8.0, 0.8 Hz, 1H), 7.696-7.638 (m, 2H), 7.525-7.482 (m, 1H), 7.369-7.329 (m, 2H), 6.508 (s, 1H), 1.021 (s, 9H); ^{13}C-NMR (99 MHz, $CDCl_3$): δ 155.489, 151.944, 149.339, 145.459, 132.669, 132.180, 129.182, 128.779, 125.330, 124.899, 121.728, 81.529, 36.521, 25.743; HRMS (ESI) calc. m/z 397.1006 (M + Na^+), found m/z 397.1004 (M + Na^+).

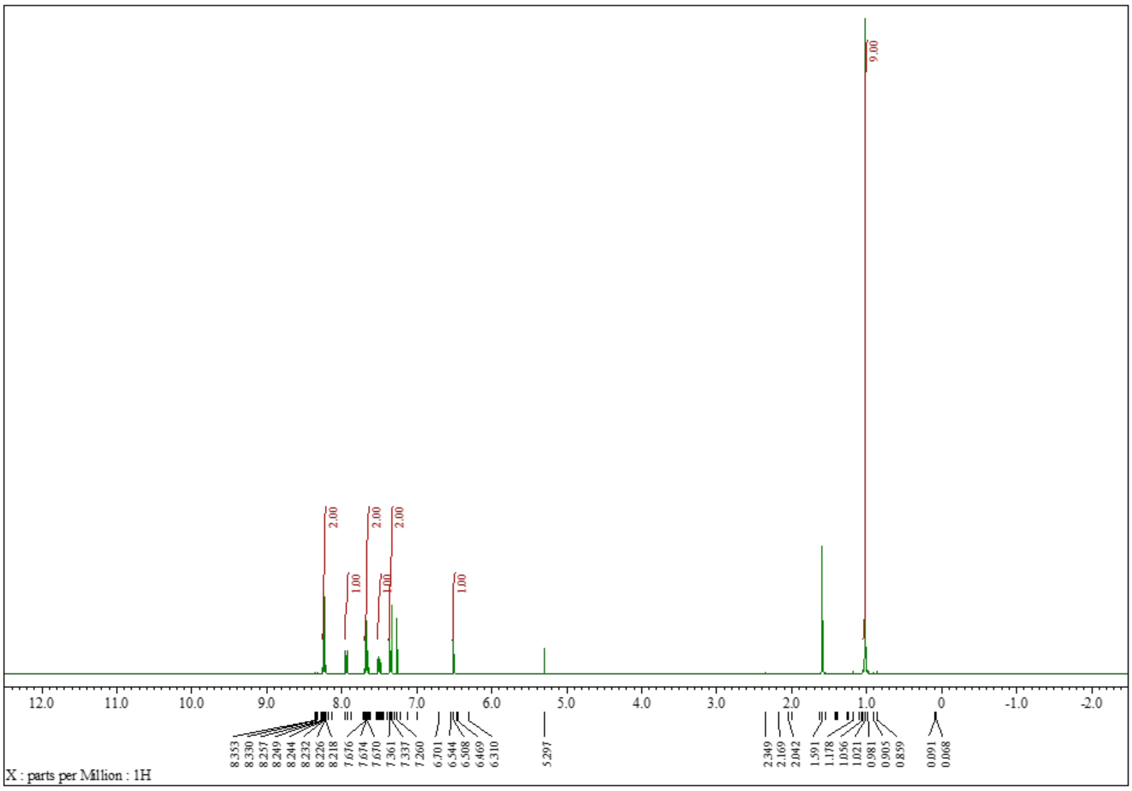

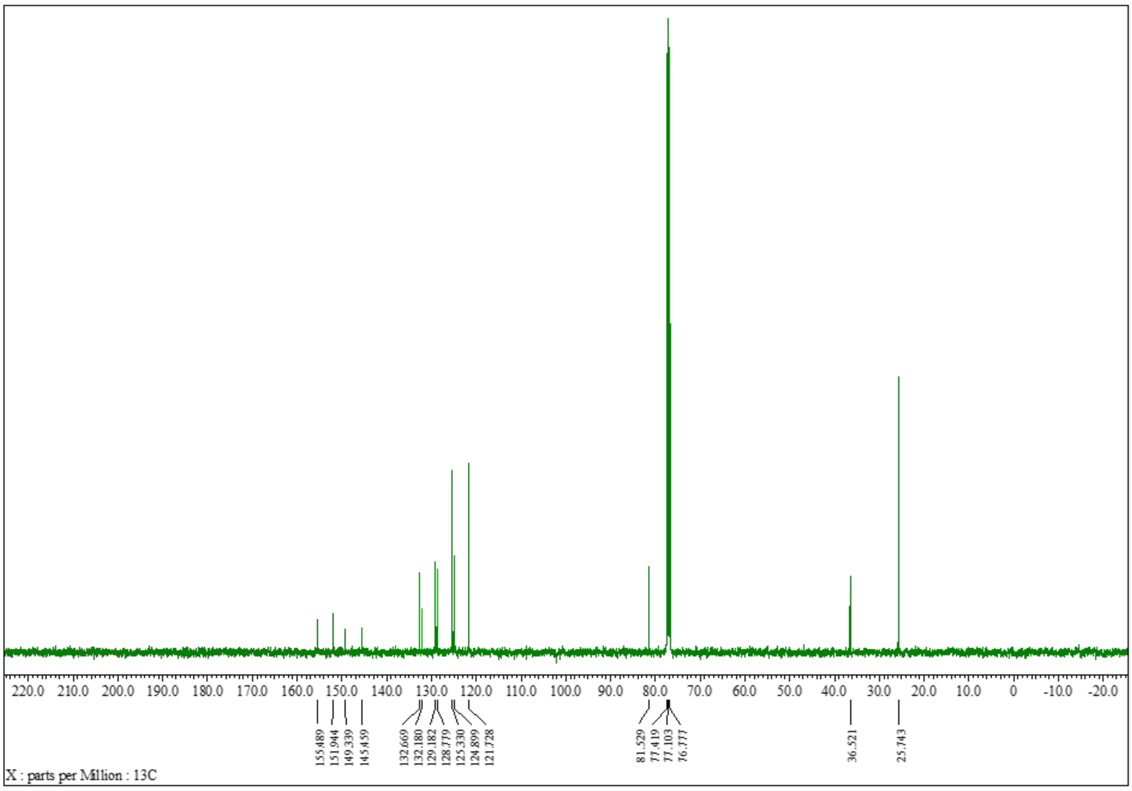

Compound 20

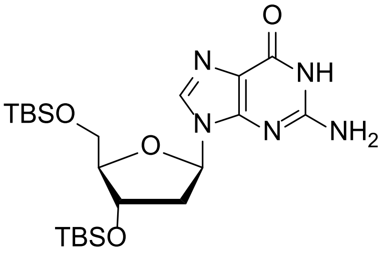

2'-deoxyguanosine hydrate (2.50 g, 8.76 mmol, 1 eq.) was dissolved in DMF (87 mL). Imidazole (3.58 g, 52.6 mmol, 6 eq.) and *tert*-butyldimethylchlorosilane (3.96 g, 26.3 mmol, 3 eq.) were added and stirred at room temperature. After stirring for 16 h, the reaction mixture was concentrated and added AcOEt then washed with saturated $NaHCO_3$ aqueous solution and brine. The precipitation was filtered and collected, and the organic layer was dried with Na_2SO_4, filtered, and concentrated in vacuo. The crude product was dissolved in dichloromethane and methanol then added hexane and AcOEt. The precipitation was collected and Compound **20** (3.84 g, 7.75 mmol, 88%) was obtained. ^{1}H-NMR (392 MHz, DMSO-d_6): δ 10.775 (br, 1H), 7.869 (s, 1H), 6.619 (br, 2H), 6.098 (dd, J = 7.8, 6.2 Hz, 1H), 4.481 (quin, J = 2.8 Hz, 1H), 3.818-3.785 (m, 1H), 3.712-3.613 (m, 2H), 2.670-2.602 (m, 1H), 2.257-2.200 (m, 1H), 0.882 (s, 9H), 0.865 (s, 9H), 0.097 (s, 6H), 0.038 (s, 3H), 0.033 (s, 3H); ^{13}C-NMR (99 MHz, DMSO-d_6): δ 157.309, 154.445, 151.523, 135.380, 117.149, 87.517, 82.640, 72.715, 63.288,

26.318, 26.212, 18.500, 18.251, -4.425, -4.933; HRMS (ESI) calc. *m*/*z* 496.2770 (M + H$^+$), found *m*/*z* 496.2788 (M + H$^+$).

※1 peak was same position of DMSO's peak (around 40 ppm), so 1 carbon peak was missing.

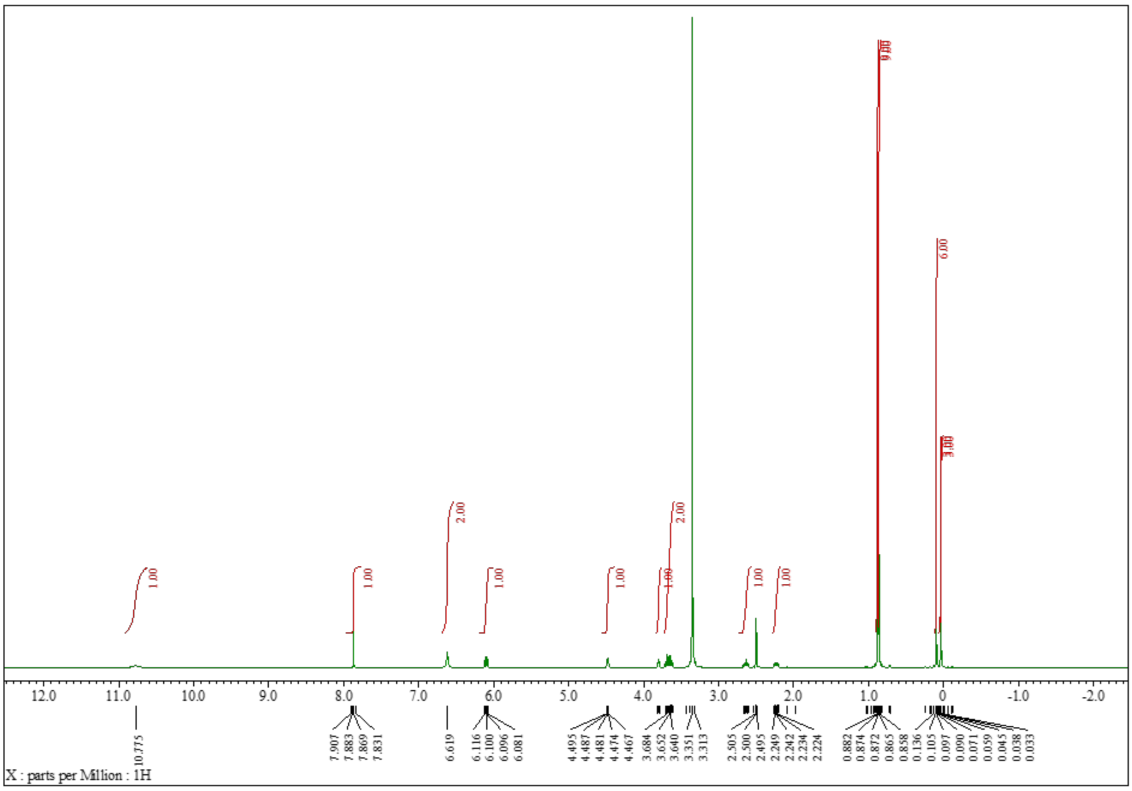

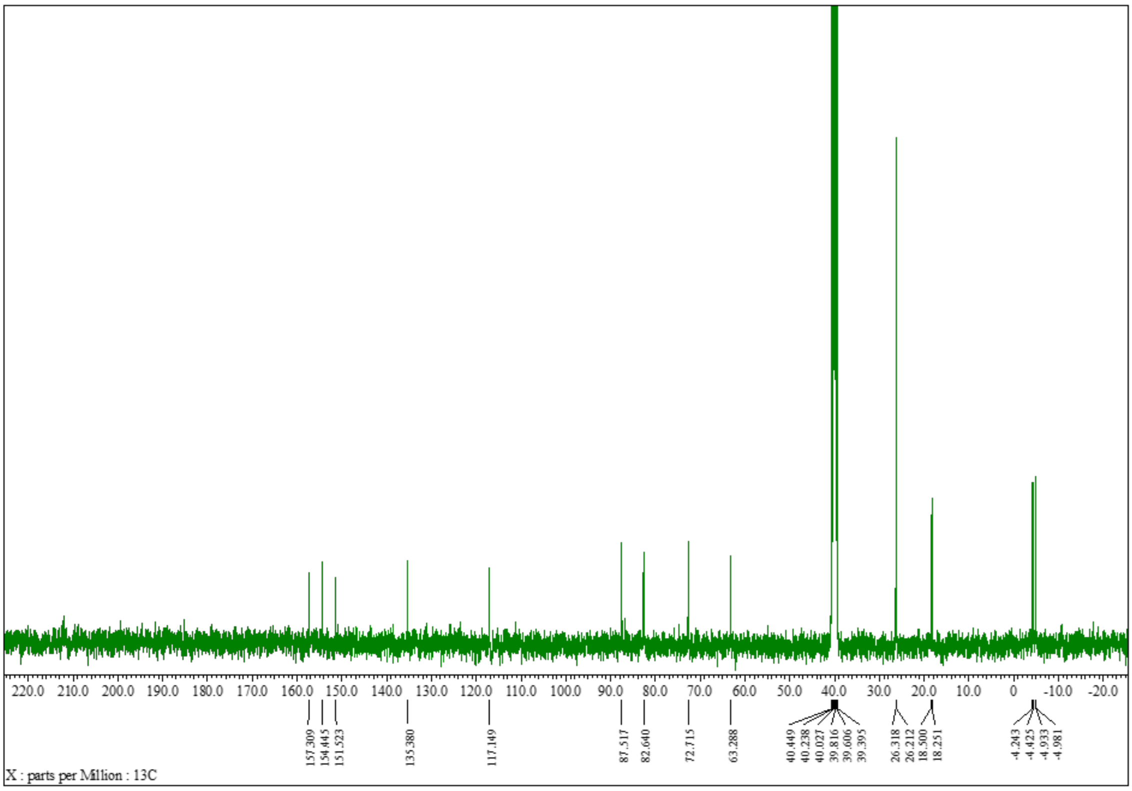

Compound 21

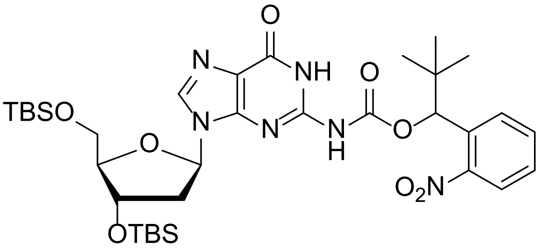

Compound **20** (100 mg, 0.202 mmol, 1.0 eq.), Compound **19** (151 mg, 0.403 mmol, 2.0 eq.), and 18-crown-6 (53.3 mg, 0.202 mmol, 1.0 eq.) were dissolved in THF (2 mL). Potassium hydride in mineral oil (30%) (27.0 mg, 0.202 mmol, 1.0 eq.) was added and stirred at room temperature. After stirring for 21 h additional, potassium hydride in mineral oil (30%) (27.0 mg, 0.202 mmol, 1.0 eq.) was added to drive the reaction to completion. After stirring for 4 h, the reaction mixture was diluted with AcOEt then washed with saturated $NaHCO_3$ aqueous solution and brine. The organic layer was dried with Na_2SO_4, filtered, and concentrated in vacuo. The crude product was purified by silica gel column chromatography (Hexane/AcOEt = 60/40→20/80) and Compound **21** (99.6 mg, 0.136 mmol, 68%) was obtained. ^{1}H-NMR (392 MHz, $CDCl_3$): δ 11.084 (br, 1H), 7.957-7.908 (m, 3H), 7.626-7.584 (m, 1H), 7.549-7.465 (m, 2H), 6.551 (s, 1H), 6.231-6.193 (m, 1H), 4.569-4.561 (m, 1H), 3.985 (q, J = 3.5 Hz, 1H), 3.750 (d, J = 3.6 Hz, 2H), 2.490-2.305 (m, 2H), 0.973 (2s, 9H), 0.916 (2s, 9H), 0.898 (s, 9H),

0.105 (s, 3H), 0.102 (s, 3H), 0.072 (2s, 3H), 0.064 (2s, 3H); ^{13}C-NMR (99 MHz, $CDCl_3$): δ 155.374, 152.615, 149.300, 147.969, 146.263, 136.741, 132.621, 132.037, 129.230, 128.616, 124.995, 121.230, 88.111, 83.771, 79.268, 72.064, 62.886, 41.321, 36.339, 26.030, 25.829, 25.781, 18.500, 18.088, -4.684, -5.307; HRMS (ESI) calc. *m*/*z* 731.3614 (M + H$^+$), found *m*/*z* 731.3610 (M + H$^+$).

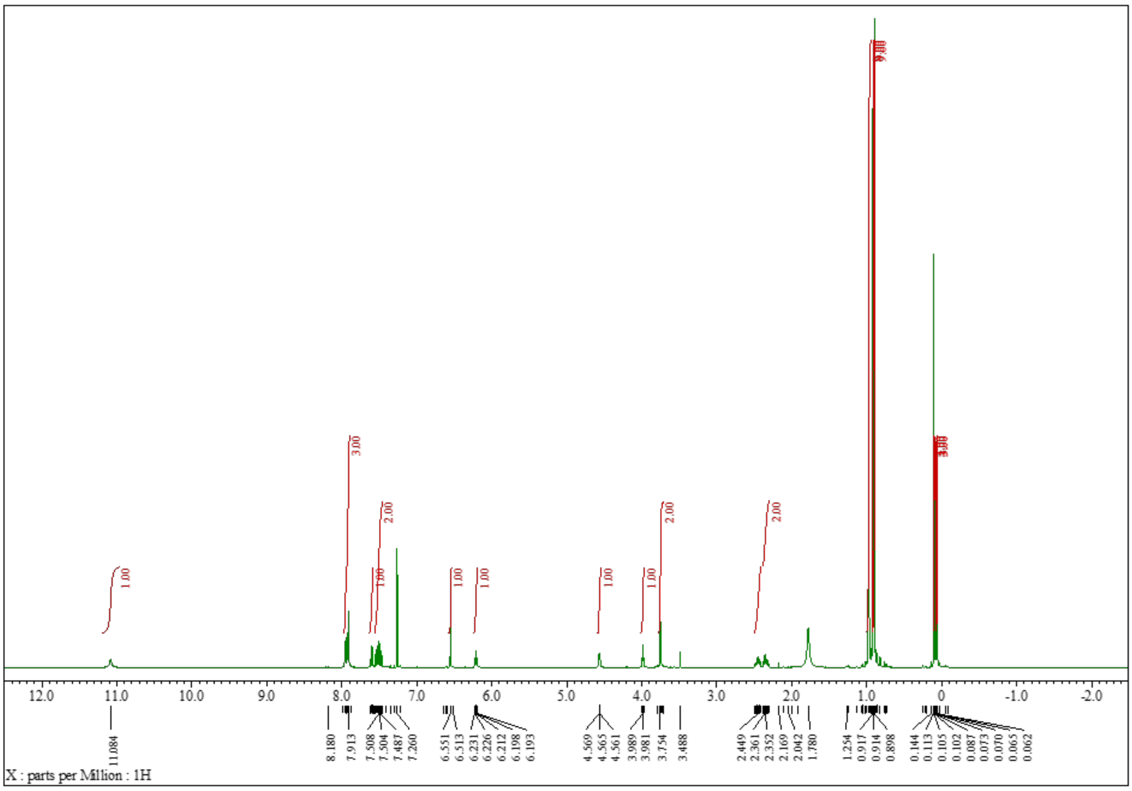

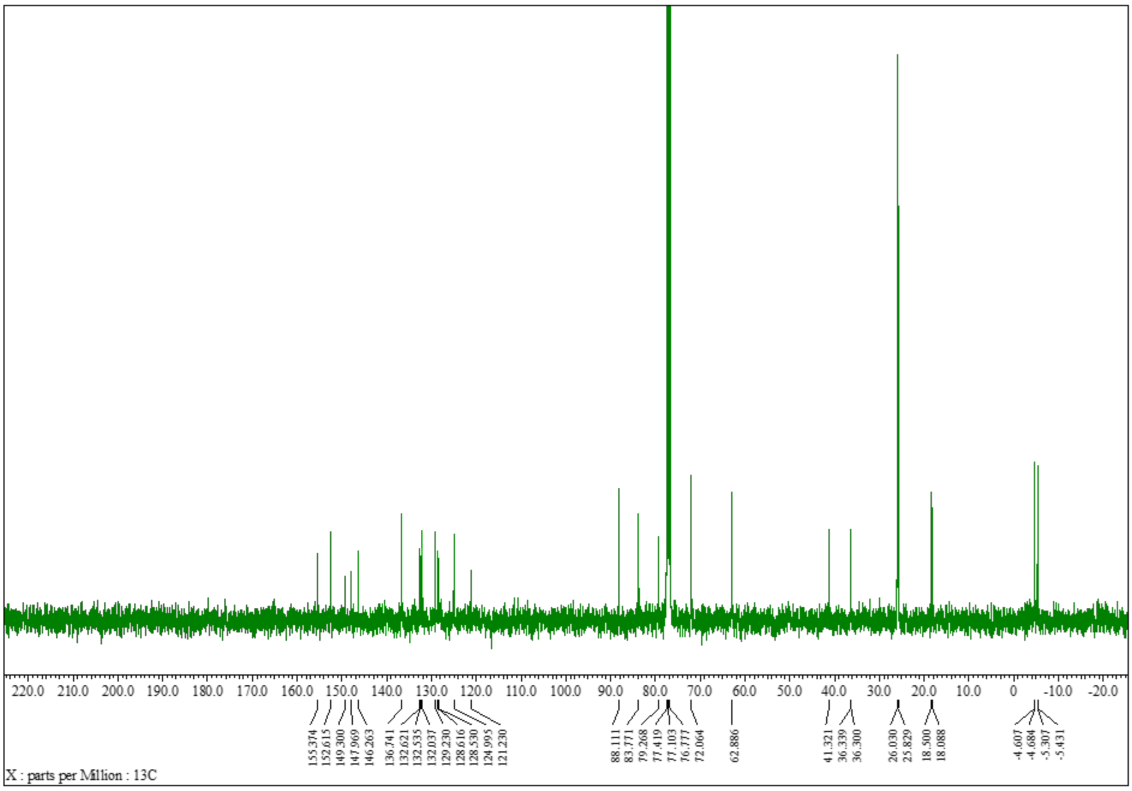

Compound 22

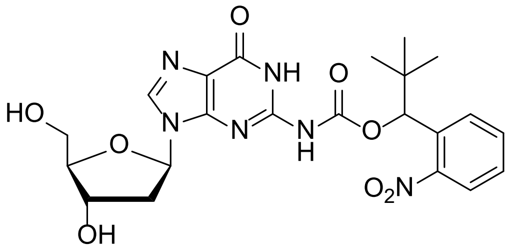

Compound **21** (85 mg, 0.116 mmol, 1.0 eq.) was dissolved in THF (1 mL). Triethylamine trihydrofluoride (28.4 μL, 0.174 mmol, 1.5 eq.) was added and stirred at room temperature. After stirring for 16 h, the reaction mixture was concentrated in vacuo. The crude product was purified by silica gel column chromatography (DCM/MeOH = 96/4→94/6) and Compound **22** (54.4 mg, 0.108 mmol, 93%) was obtained. ^{1}H-NMR (392 MHz, DMSO-d_6): δ 11.629 (br, 1H), 11.145 (br, 1H), 8.205 (s, 1H), 7.998 (dd, J = 8.2, 1.0 Hz, 1H), 7.802 (t, J = 7.4 Hz, 1H), 7.662-7.600 (m, 2H), 6.269 (s, 1H), 6.196 (t, J = 7.0 Hz, 1H), 5.313 (d, J = 4.0 Hz, 1H), 4.949 (t, J = 5.6 Hz, 1H), 4.385-4.346 (m, 1H), 3.848-3.818 (m, 1H), 3.595-3.474 (m, 2H), 2.599-2.533 (m, 1H), 2.283-2.226 (m, 1H), 0.919 (s, 9H); ^{13}C-NMR (99 MHz, DMSO-d_6): δ 155.546, 154.306, 149.318, 149.042, 147.573, 137.940, 133.658, 132.055, 129.995, 129.309, 125.084, 120.458, 88.269, 83.510, 77.682, 71.016, 62.003, 36.452,

25.837; HRMS (ESI) calc. *m*/*z* 503.1885 (M + H$^+$), found *m*/*z* 503.1874 (M + H$^+$).

※1 peak was same position of DMSO's peak (around 40 ppm), so 1 carbon peak was missing.

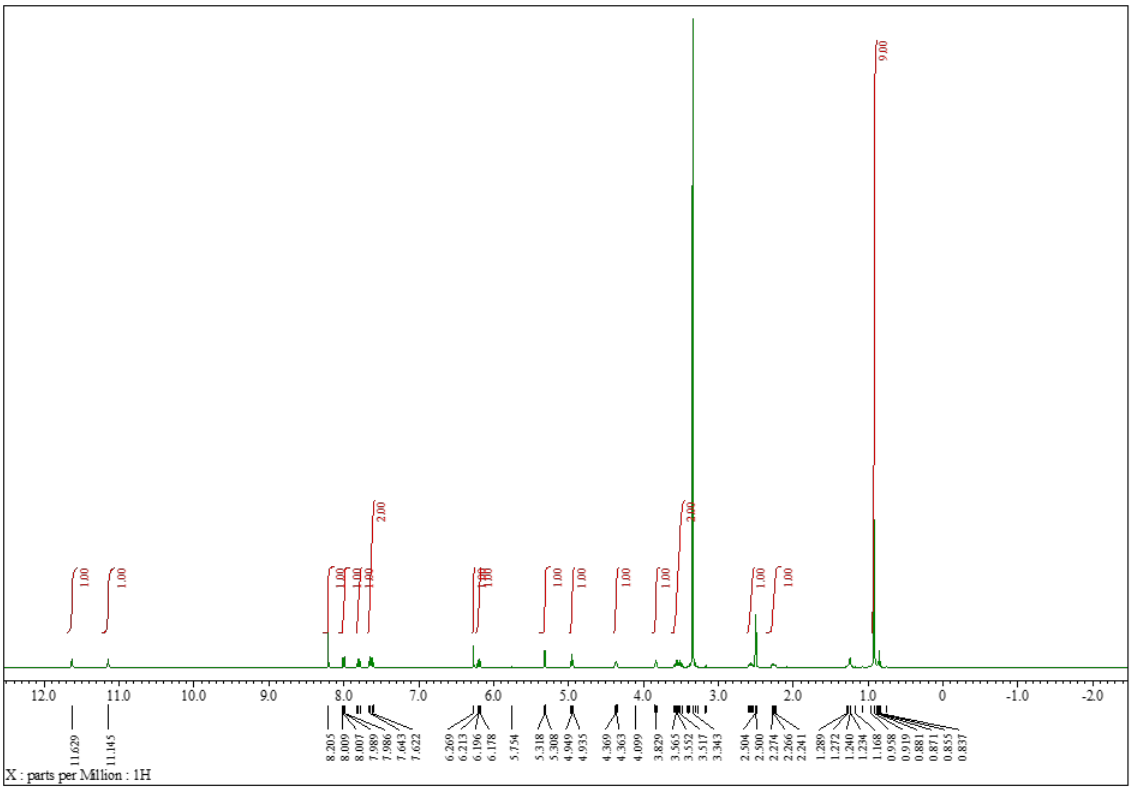

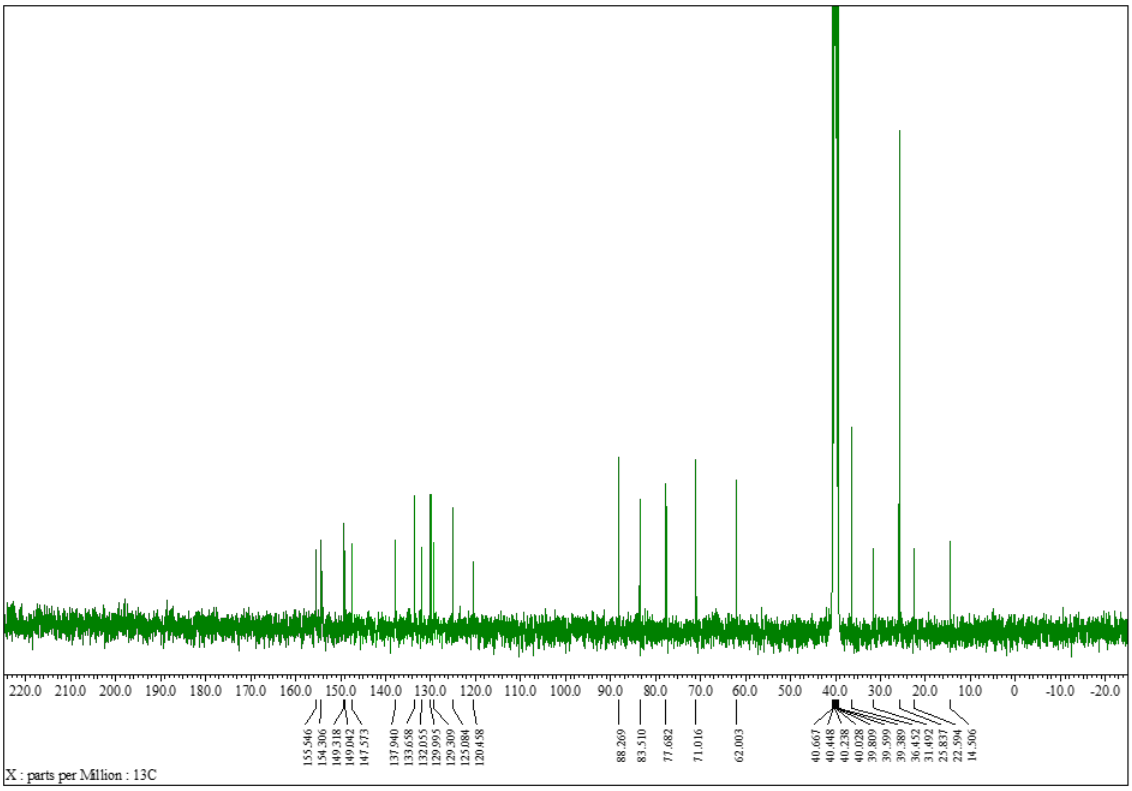

Compound 23

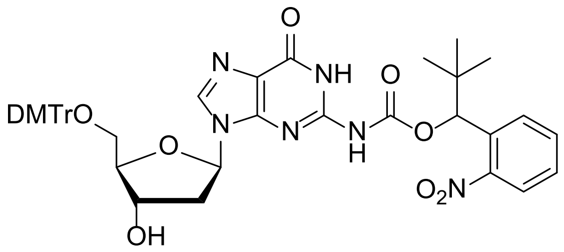

Compound **22** (350 mg, 0.697 mmol, 1.0 eq.) was dissolved in pyridine (1.75 mL). 4-dimethylaminopyridine (1.70 mg, 0.0139 mmol, 0.020 eq.) and 4,4'-dimethoxytrityl chloride (260 mg, 0.766 mmol, 1.1 eq.) were added and stirred at room temperature. After stirring for 18 h, the reaction mixture was diluted with AcOEt then washed with saturated $NaHCO_3$ aqueous solution and brine. The organic layer was dried with Na_2SO_4, filtered, and concentrated in vacuo. The crude product was purified by silica gel column chromatography (AcOEt/MeOH = 100/0→98/2) and Compound **23** (438 mg, 0.544 mmol, 78%) was obtained. ^{1}H-NMR (392 MHz, $CDCl_3$): δ 11.200 (br, 1H), 8.680 (2s, 1H), 7.910-7.871 (m, 1H), 7.707 (2s, 1H), 7.582-7.525 (m, 1H), 7.495-7.423 (m, 2H), 7.402-7.362 (m, 2H), 7.289-7.127 (m, 7H), 6.774-6.726 (m, 4H), 6.483 (s, 1H), 6.179 (q, *J* = 6.4 Hz, 1H), 4.629 (dd, *J* = 5.6, 3.6 Hz, 1H), 4.109 (m, 1H), 3.736-3.714 (m, 6H), 3.303-3.292 (m, 2H), 2.707-2.622 (m, 1H), 2.461-2.395 (m, 1H), 0.890 (2s, 9H); ^{13}C-NMR (99 MHz, $CDCl_3$): δ 158.627, 155.622, 153.076, 149.185, 148.307, 146.543, 144.673, 137.435, 135.746, 132.723, 132.074, 130.062, 129.127, 128.755, 128.135, 127.964, 127.077, 124.807, 121.183, 113.267, 86.552, 86.285, 84.187, 79.065, 72.294, 64.006, 55.288, 40.219, 36.213, 25.713; HRMS (ESI) calc. *m*/*z* 805.3192 (M + H^+), found *m*/*z* 805.3205 (M + H^+).

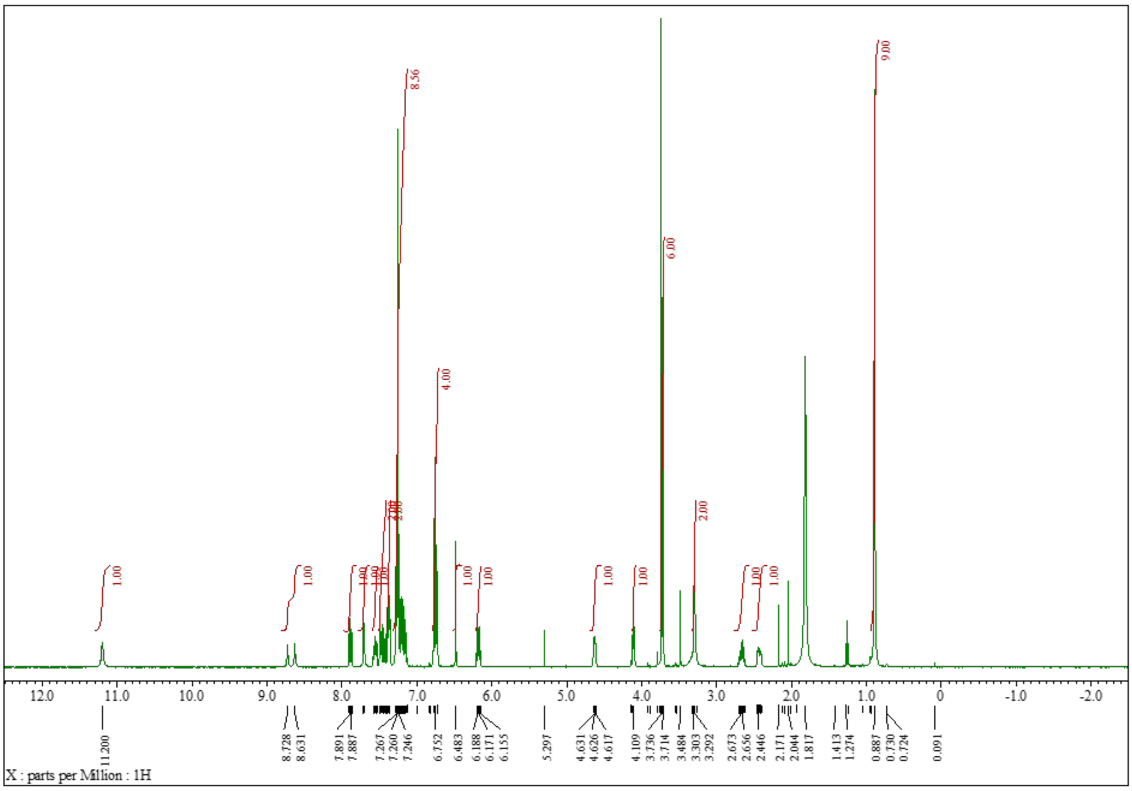

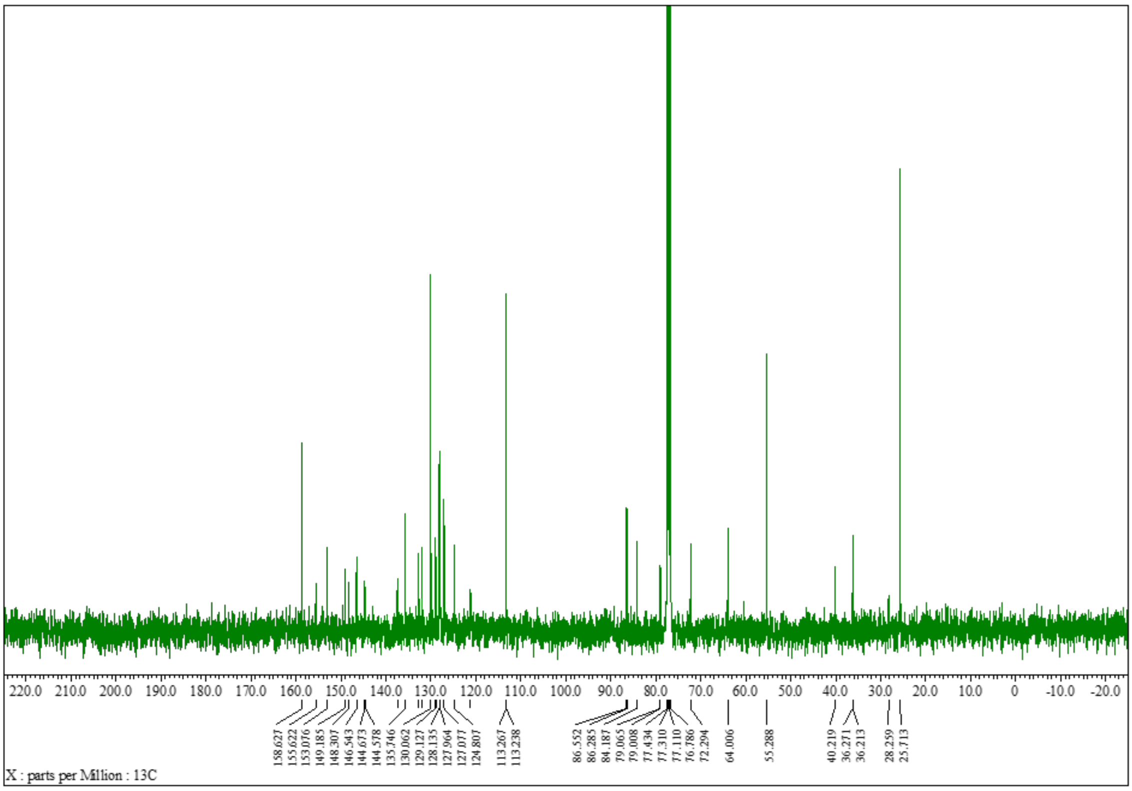

Compound 24

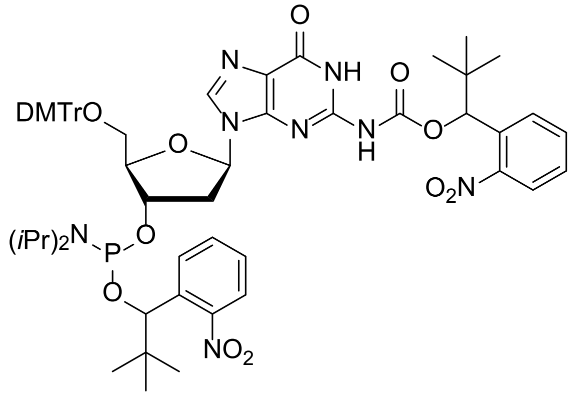

Compound **23** (400 mg, 0.497 mmol, 1.0 eq.) was co-evaporated with pyridine and dissolved in dichloromethane (1.25 mL). Compound **4** (328 mg, 0.745 mmol, 1.5 eq.) and 5-(methylthio)-1*H*-tetrazole (173 mg, 1.49 mmol, 3.0 eq.) were added at 0 °C then stirred at room temperature. After stirring for 19 h, the reaction mixture was diluted with AcOEt then washed with saturated $NaHCO_3$ aqueous solution and brine. The organic layer was dried with Na_2SO_4, filtered, and concentrated in vacuo. The crude product was purified by silica gel column chromatography (DCM/ACN = 100/0→95/5 with 1% TEA) and Compound **24** (194 mg, 0.170 mmol, 34%) was obtained. ^{1}H-NMR (392 MHz, DMSO-d_6) (diastereomixture): δ 11.272-11.108 (br, 2H), 8.115-7.434 (m, 9H), 7.285-7.089 (m, 9H), 6.825-6.620 (m, 4H), 6.284-6.255 (m, 1H), 6.218-5.774 (m, 1H), 5.518-5.163 (m, 1H), 4.863-4.311 (m, 1H), 4.122-3.818 (m, 1H), 3.712-3.667 (m, 6H), 3.637-3.469 (m, 1H), 3.223-2.668 (m, 4H), 2.462-2.117 (m, 1H), 1.249-0.740 (m, 30H); ^{13}C-NMR (99 MHz, DMSO-d_6): δ 158.550, 155.565, 154.459, 149.414, 149.337, 147.907, 145.227, 138.074, 135.842, 134.516, 133.581, 132.818, 132.208, 130.901, 130.129, 129.948, 129.509, 129.356, 128.994, 128.174, 127.163, 125.064, 124.311, 124.034, 120.858, 113.457, 86.009, 85.789, 83.586, 77.511, 75.412, 73.143, 64.282, 55.451, 50.024, 43.405, 36.862, 36.433, 25.855, 24.940, 24.692; ^{31}P-NMR (159 MHz, DMSO-d_6): δ 152.601, 152.226, 148.752, 148.324, 141.242, 141.055, 140.922, 140.735; HRMS (ESI) calc. *m*/*z* 1143.4951 (M + H$^+$), found *m*/*z* 1143.4901 (M + H$^+$).

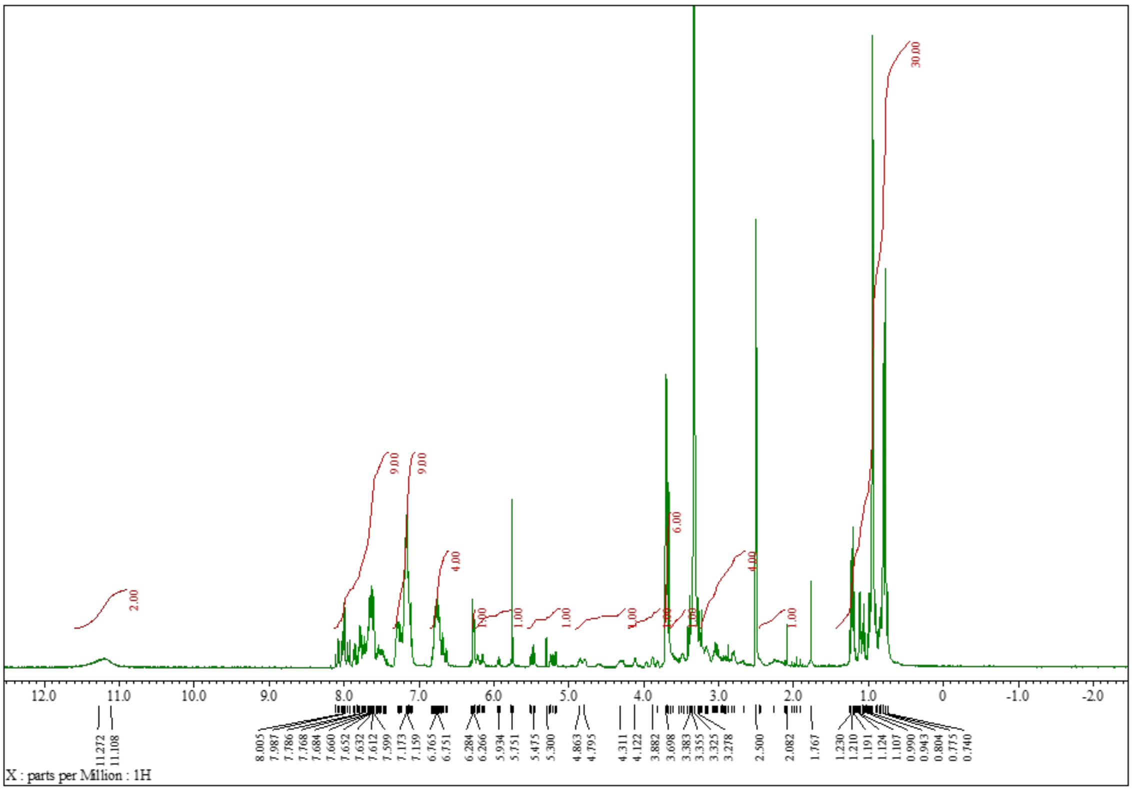

X : parts per Million : 1H

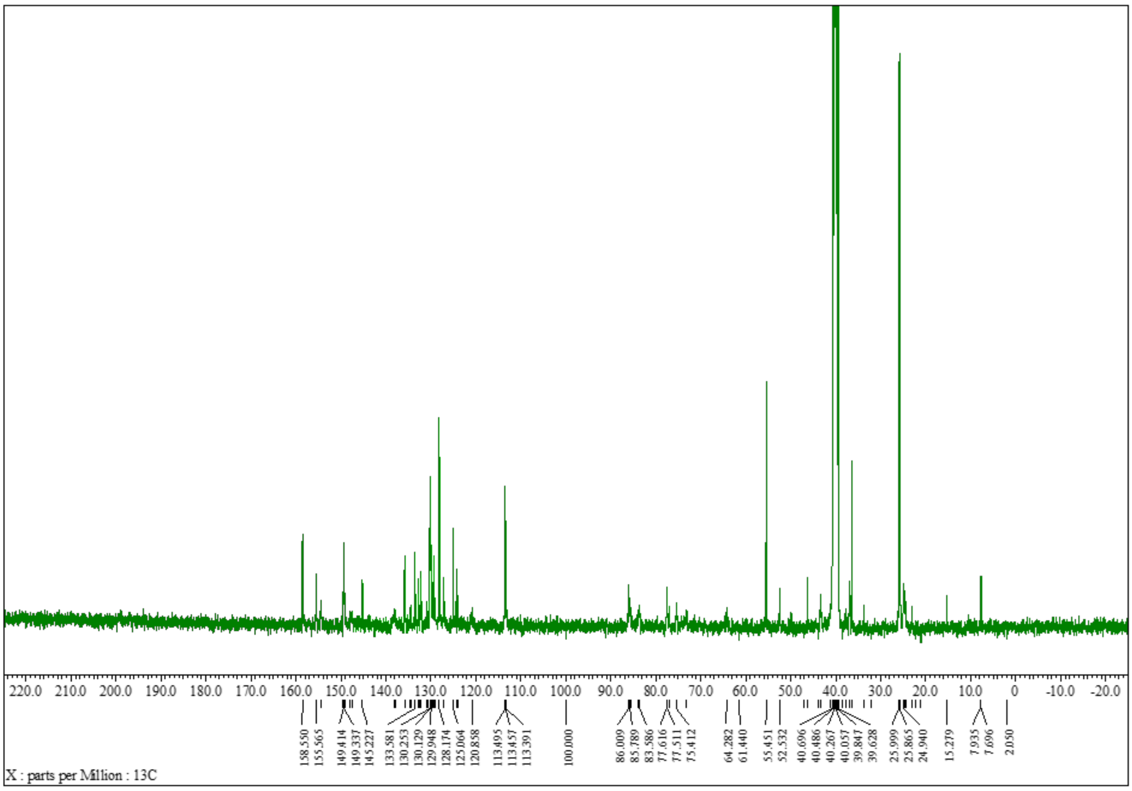

X : parts per Million : 13C

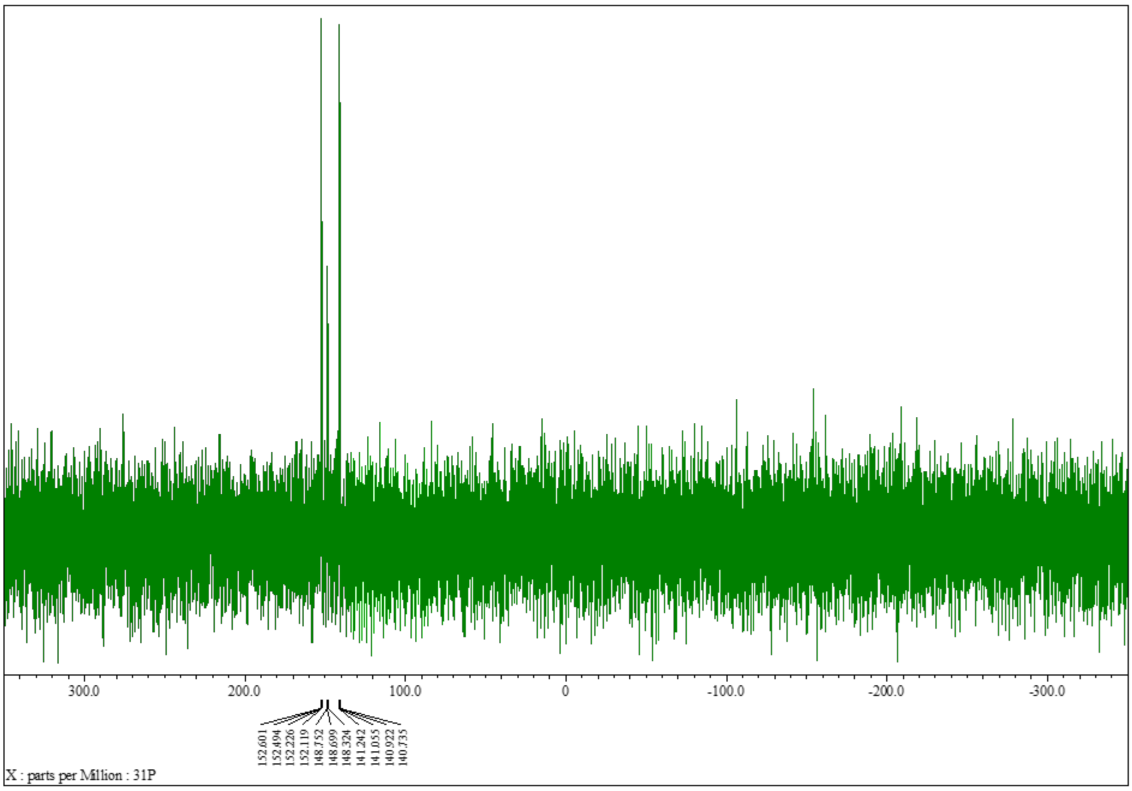

Compound 25

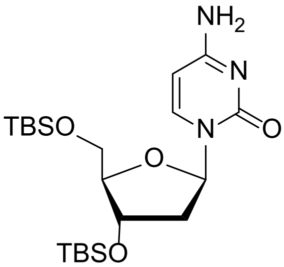

2'-deoxycytidine (2.50 g, 11.0 mmol, 1 eq.) was dissolved in DMF (110 mL). Imidazole (4.49 g, 66.0 mmol, 6 eq.) and *tert*-butyldimethylchlorosilane (4.97 g, 33.0 mmol, 3 eq.) were added and stirred at room temperature. After stirring for 20 h, the reaction mixture was diluted with AcOEt then washed with saturated $NaHCO_3$ aqueous solution and brine. The organic layer was dried with Na_2SO_4, filtered, and concentrated in vacuo. The crude product was purified by silica gel column chromatography (AcOEt/MeOH = 100/0→99:1→98:2→96:4) and Compound **25** (4.95 g, 10.7 mmol, 98%) was obtained. ^{1}H-NMR (392 MHz, $CDCl_3$): δ 7.978 (d, *J* = 7.6 Hz, 1H), 6.247 (dd, *J* = 6.4, 5.2 Hz, 1H), 5.639 (d, *J* = 7.6 Hz, 1H), 4.356 (dd, *J* = 10.4, 6.0 Hz, 1H), 3.923-3.737 (m, 3 H), 2.432-2.368 (m, 1H), 2.096-2.034 (m, 1H), 0.911 (s, 9H), 0.865 (s, 9H), 0.094 (s, 3H), 0.090 (s, 3H), 0.045 (s, 3H), 0.042 (s, 3H); ^{13}C-NMR (99 MHz, $CDCl_3$): δ 165.721, 155.949, 141.492, 93.763, 87.344, 86.013,

70.301, 62.004, 42.279, 25.992, 25.800, 18.433, 18.031, -4.847, -5.393; HRMS (ESI) calc. *m*/*z* 456.2708 (M + H^+), 478.2528 (M + Na^+), 494.2267 (M + K^+), found *m*/*z* 456.2724 (M + H^+), 478.2543 (M + Na^+), 494.2283 (M + K^+).

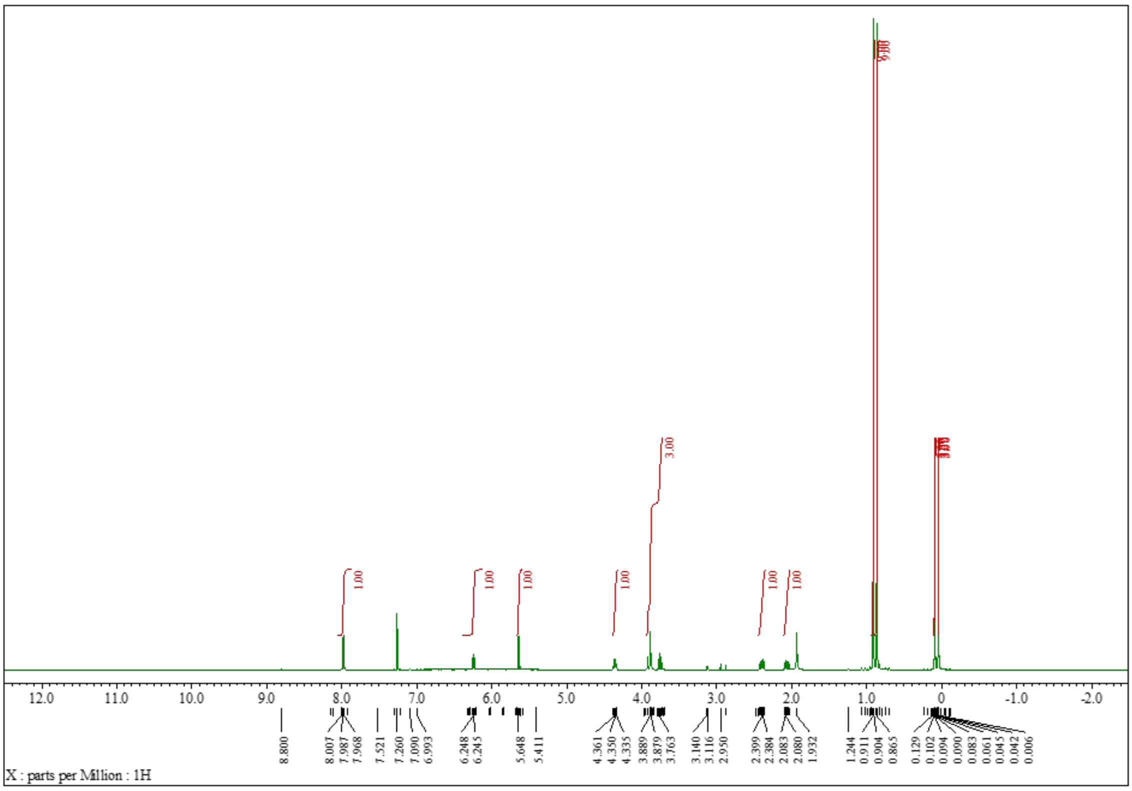

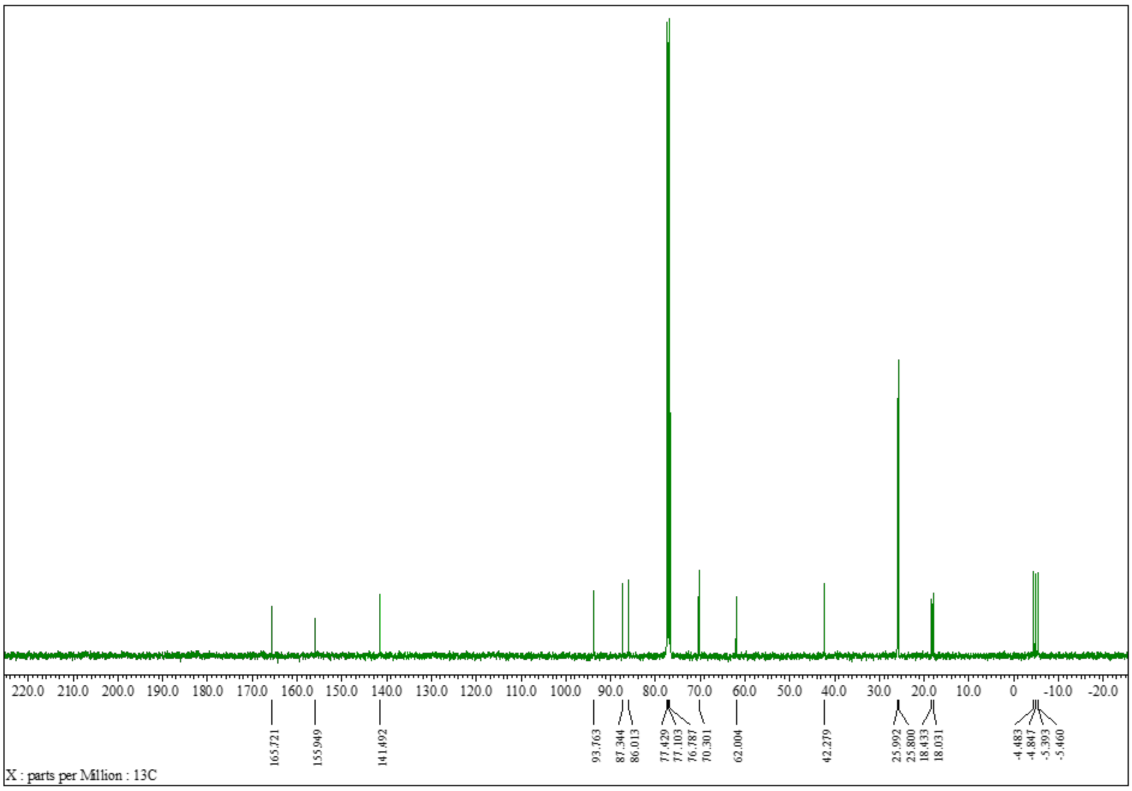

Compound 27

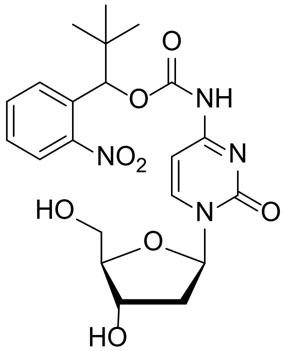

Compound **13** (998 mg, 3.29 mmol, 1.5 eq.) was dissolved in dichloromethane (20 mL). Triethyloxonium tetrafluoroborate (625 mg, 3.29 mmol, 1.5 eq.) was added and stirred at room temperature. After stirring for 2 h, Compound **26** (1.00 g, 4.78 mmol, 1 eq.) was added and stirred at room temperature. After stirring for 23 h, the reaction mixture was diluted with AcOEt then washed with saturated $NaHCO_3$ aqueous solution and brine. The organic layer was dried with Na_2SO_4, filtered, and concentrated in vacuo. The crude product was dissolved in THF (20 mL) and added triethylamine trihydrofluoride (1.79 mL, 11.0 mmol, 5.0 eq.). After stirring for 14 h at room temperature, the reaction mixture was concentrated and purified by silica gel column chromatography (DCM/MeOH =

99/1→95/5→90/10) and Compound **27** (920 mg, 1.99 mmol, 91%) was obtained. ^{1}H-NMR (392 MHz, DMSO-d_6): δ 11.008 (br, 1H), 8.274 (d, J = 7.6 Hz, 1H), 7.972 (dd, J = 8.4, 1.2 Hz, 1H), 7.804-7.765 (m, 1H), 7.668-7.639 (m, 1H), 7.621-7.575 (m, 1H), 6.907 (d, J = 5.2 Hz, 1H), 6.213 (d, J = 9.6 Hz, 1H), 6.083 (td, J = 6.4, 2.0 Hz, 1H), 5.259 (dd, J = 4.4, 2.8 Hz, 1H), 5.031 (td, J = 5.2, 2.0 Hz, 1H), 4.222-4.162 (m, 1H), 3.837 (q, J = 3.6 Hz, 1H), 3.623-3.513 (m, 2H), 2.301-2.226 (m, 1H), 2.033-1.941 (m, 1H), 0.889 (2s, 9H); ^{13}C-NMR (99 MHz, DMSO-d_6): δ 163.081, 154.697, 153.028, 149.299, 145.379, 133.591, 132.570, 129.814, 129.337, 125.007, 94.506, 88.393, 86.609, 76.853, 70.434, 61.431, 41.373, 36.385, 25.818; HRMS (ESI) calc. m/z 463.1823 (M + H$^+$), found m/z 463.1836 (M + H$^+$).

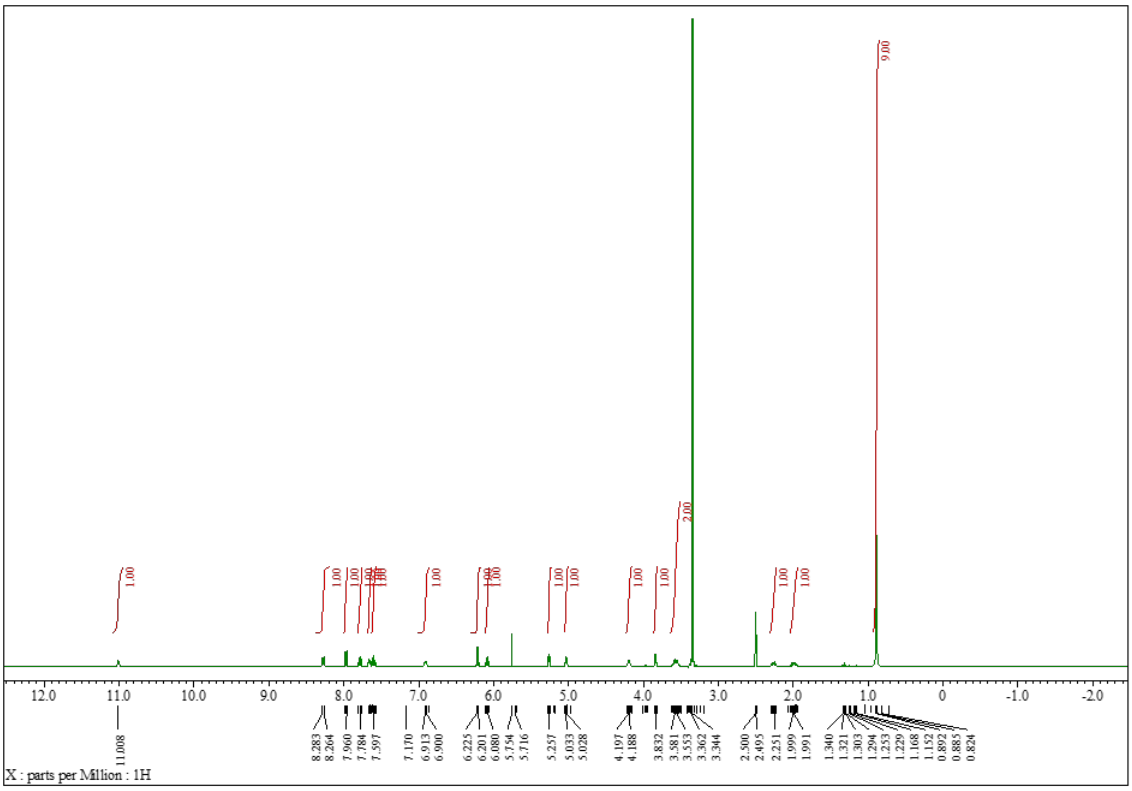

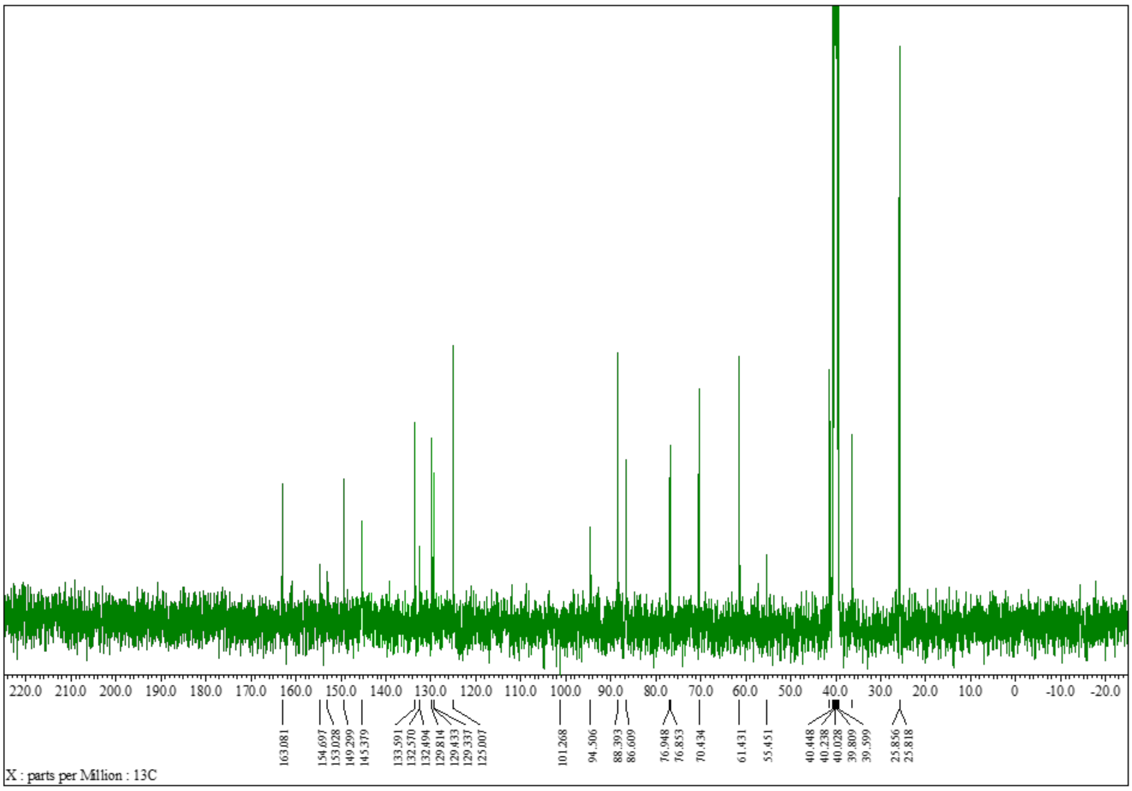

Compound 28

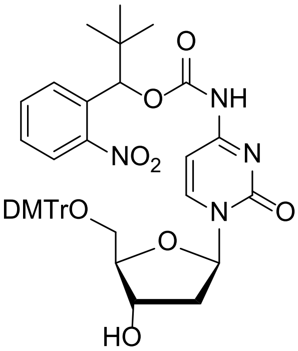

Compound **27** (900 mg, 1.95 mmol, 1.0 eq.) was dissolved in pyridine (5 mL). 4-dimethylaminopyridine (4.76 mg, 0.0390 mmol, 0.020 eq.) and 4,4'-dimethoxytrityl chloride (725 mg, 2.14 mmol, 1.1 eq.) were added and stirred at room temperature. After stirring for 24 h, the reaction mixture was diluted with AcOEt then washed with saturated $NaHCO_3$ aqueous solution and brine. The organic layer was dried with Na_2SO_4, filtered, and concentrated in vacuo. The crude product was purified by silica gel column chromatography (AcOEt/MeOH = 100/0→99/1) and Compound **28** (1.26 g, 1.64 mmol, 84%) was obtained. ^{1}H-NMR (392 MHz, $CDCl_3$): δ 8.203 (t, J = 7.6 Hz, 1H), 8.077 (br, 1H), 7.910 (d, J = 8.4 Hz, 1H), 7.619-7.581 (m, 2H), 7.470-7.432 (m, 1H), 7.374-7.346 (m, 2H), 7.286-

7.172 (m, 7H), 6.930 (m, 1H), 6.835-6.796 (m, 4H), 6.497 (s, 1H), 6.249 (q, J = 5.6 Hz, 1H), 4.491-4.442 (m, 1H), 4.108 (m, 1H), 3.787-3.734 (m, 6H), 3.506-3.479 (m, 1H), 3.401-3.355 (m, 1H), 2.721-2.640 (m, 1H), 2.258-2.149 (m, 1H), 0.945 (s, 9H); ^{13}C-NMR (99 MHz, $CDCl_3$): δ 162.270, 158.722, 155.203, 151.512, 149.814, 144.607, 144.063, 136.166, 135.498, 132.713, 130.062, 129.032, 128.221, 128.107, 127.220, 124.712, 123.882, 113.381, 94.907, 87.077, 86.438, 77.997, 70.796, 70.691, 62.613, 55.288, 41.993, 36.318, 25.789; HRMS (ESI) calc. *m*/*z* 765.3130 (M + H$^+$), found *m*/*z* 765.3157 (M + H$^+$).

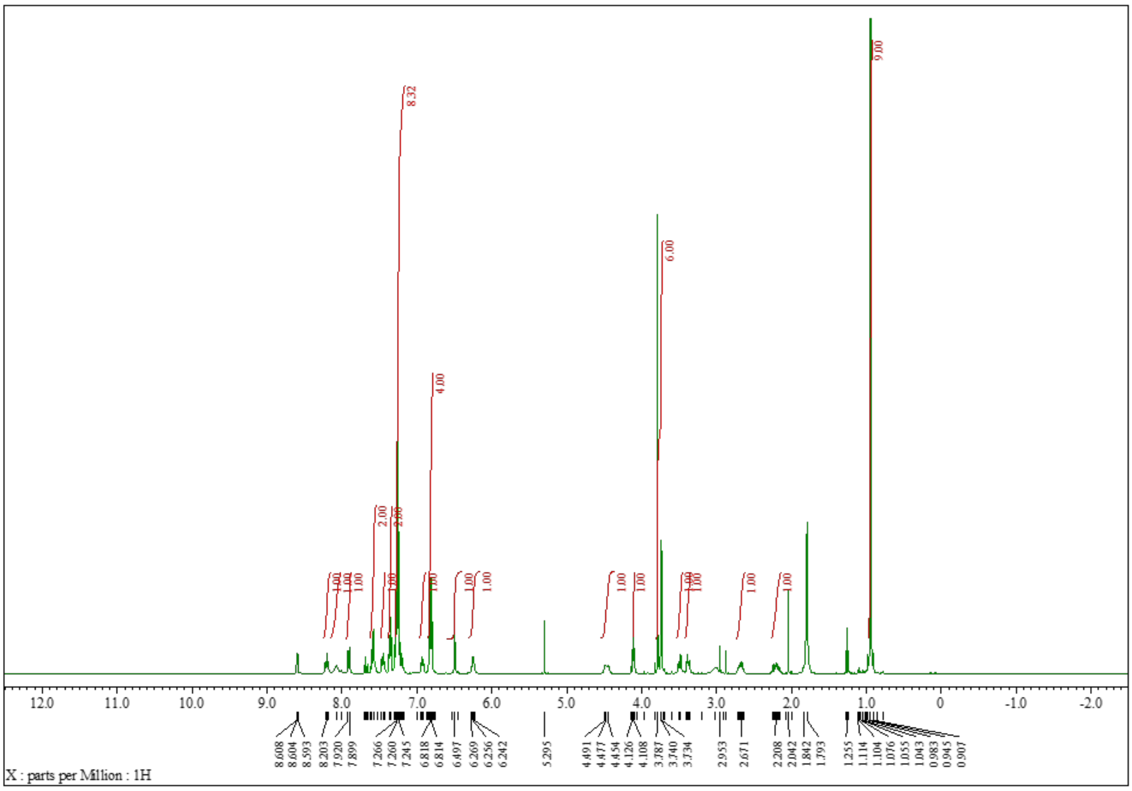

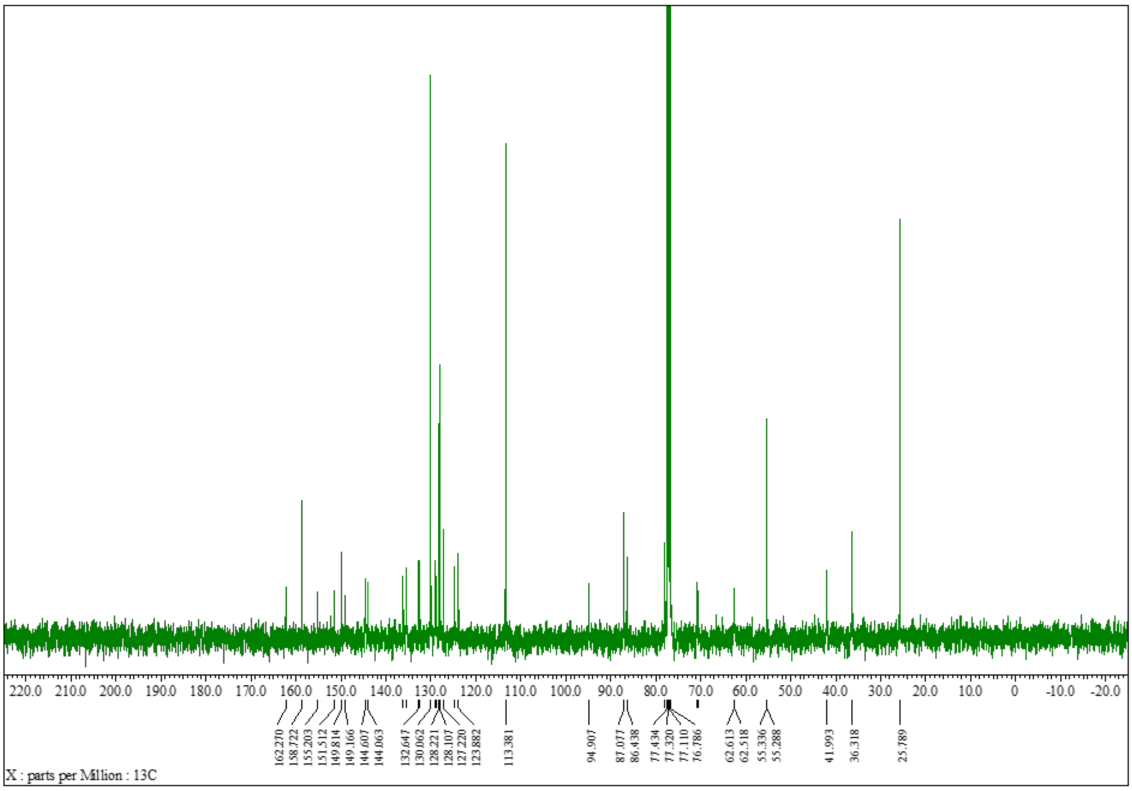

Compound 29

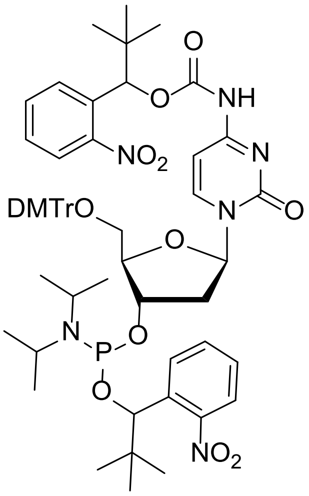

Compound **28** (500 mg, 0.654 mmol, 1.0 eq.) was co-evaporate with benzene and dissolved in dichloromethane (1.6 mL). Compound **4** (431 mg, 0.980 mmol, 1.5 eq.) and 5-(methylthio)-1*H*-tetrazole (228 mg, 1.96 mmol, 3.0 eq.) were added at 0 °C then stirred at room temperature. After stirring for 16 h, the reaction mixture was diluted with AcOEt then washed with saturated $NaHCO_3$ aqueous solution and brine. The organic layer was dried with Na_2SO_4, filtered, and concentrated in

vacuo. The crude product was purified by silica gel column chromatography (Hexane/AcOEt = 88/12→78/22→32/68 with 1% TEA) and Compound **29** (332 mg, 0.301 mmol, 46%) was obtained. ^{1}H-NMR (392 MHz, DMSO-d_6) (diastereomixture): δ 11.037 (br, 1H), 8.142-7.949 (m, 2H), 7.877-7.710 (m, 2H), 7.690-7.573 (m, 4H), 7.552-7.398 (m, 1H), 7.339-7.140 (m, 9H), 6.860-6.802 (m, 4H), 6.766-6.688 (m, 1H), 6.231-6.205 (m, 1H), 6.180-5.966, 5.777-5.665 (m, 1H), 5.513-5.448, 5.313-5154 (m, 1H), 4.816-4.502 (m, 1H), 4.233-3.836 (m, 1H), 3.719-3.655 (m, 6H), 3.612-3.055 (m, 4H), 2.599-1.767 (m, 2H), 1.271-0.718 (m, 30 H); ^{13}C-NMR (99 MHz, DMSO-d_6): δ 163.214, 158.665, 154.459, 152.933, 149.309, 144.797, 135.670, 134.707, 134.592, 133.572, 132.742, 132.485, 132.284, 130.196, 129.824, 129.404, 129.270, 128.908, 128.355, 128.202, 127.334, 124.998, 124.321, 113.686, 94.640, 86.485, 85.207, 79.161, 76.919, 75.288, 72.275, 62.918, 55.451, 49.642, 43.224, 36.900, 36.395, 26.018, 25.818, 24.654; ^{31}P-NMR (159 MHz, DMSO-d_6): δ 152.173, 151.077, 149.073, 147.496, 141.510, 141.162, 140.521, 139.906; HRMS (ESI) calc. *m/z* 1103.4889 (M + H$^+$), found *m/z* 1103.4868 (M + H$^+$).

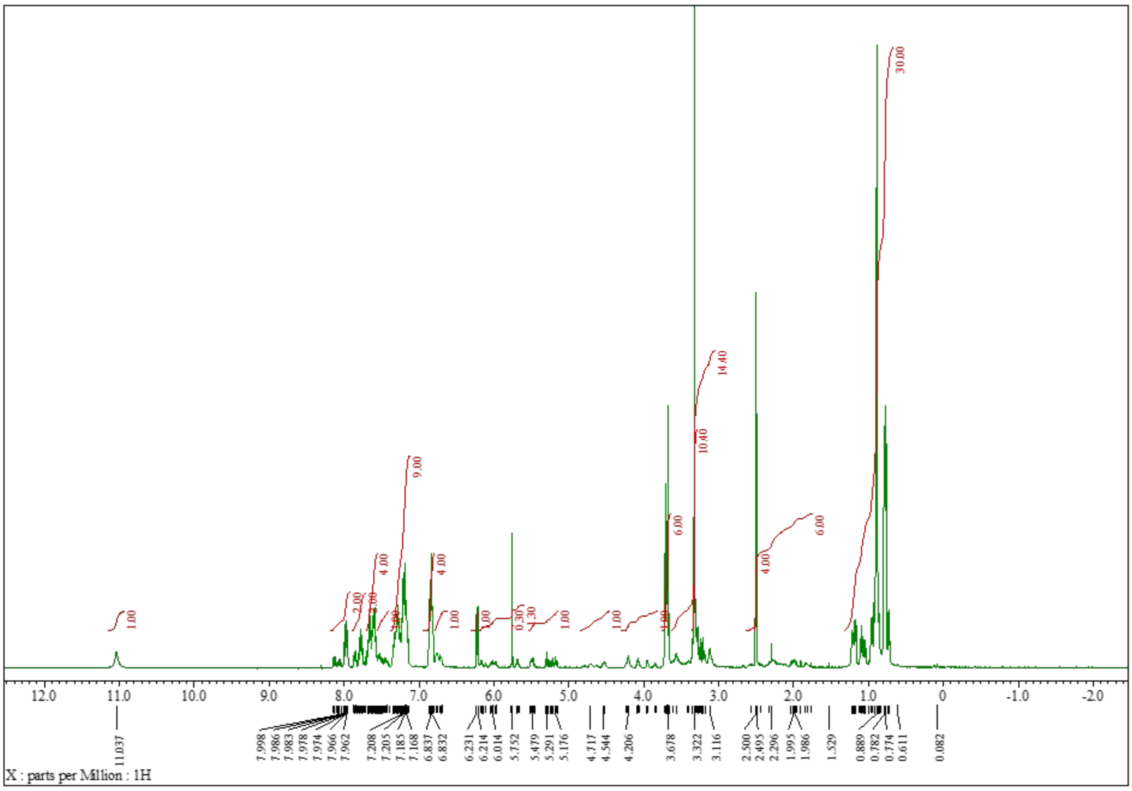

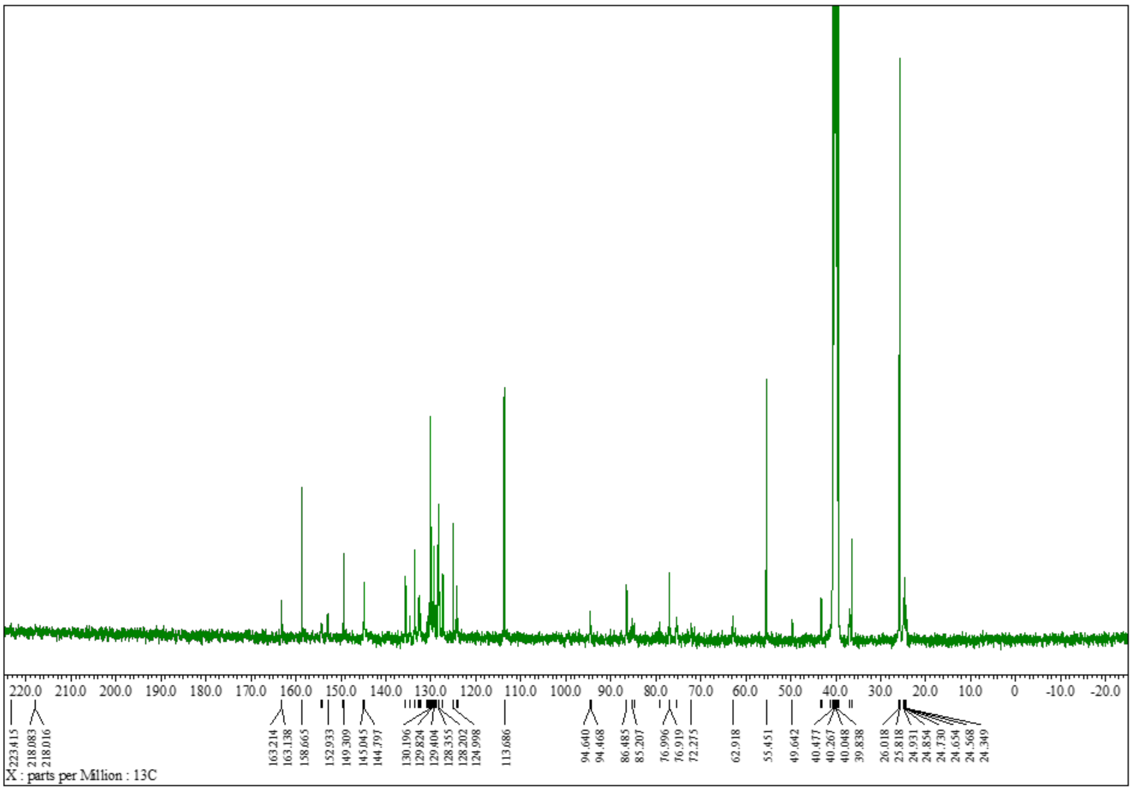
220.0 210.0 200.0 190.0 180.0 170.0 160.0 150.0 140.0 130.0 120.0 110.0 100.0 90.0 80.0 70.0 60.0 50.0 40.0 30.0 20.0 10.0 0 -10.0 -20.0
223.415
218.083
218.016
163.214
163.138
158.665
152.933
149.309
145.045
144.797
130.196
129.824
129.404
128.355
128.202
124.998
113.686
94.640
94.468
86.485
85.207
76.996
76.919
72.275
62.918
55.451
49.642
40.477
40.267
40.048
39.838
26.018
25.818
24.931
24.854
24.730
24.654
24.568
24.349
X : parts per Million : 13C

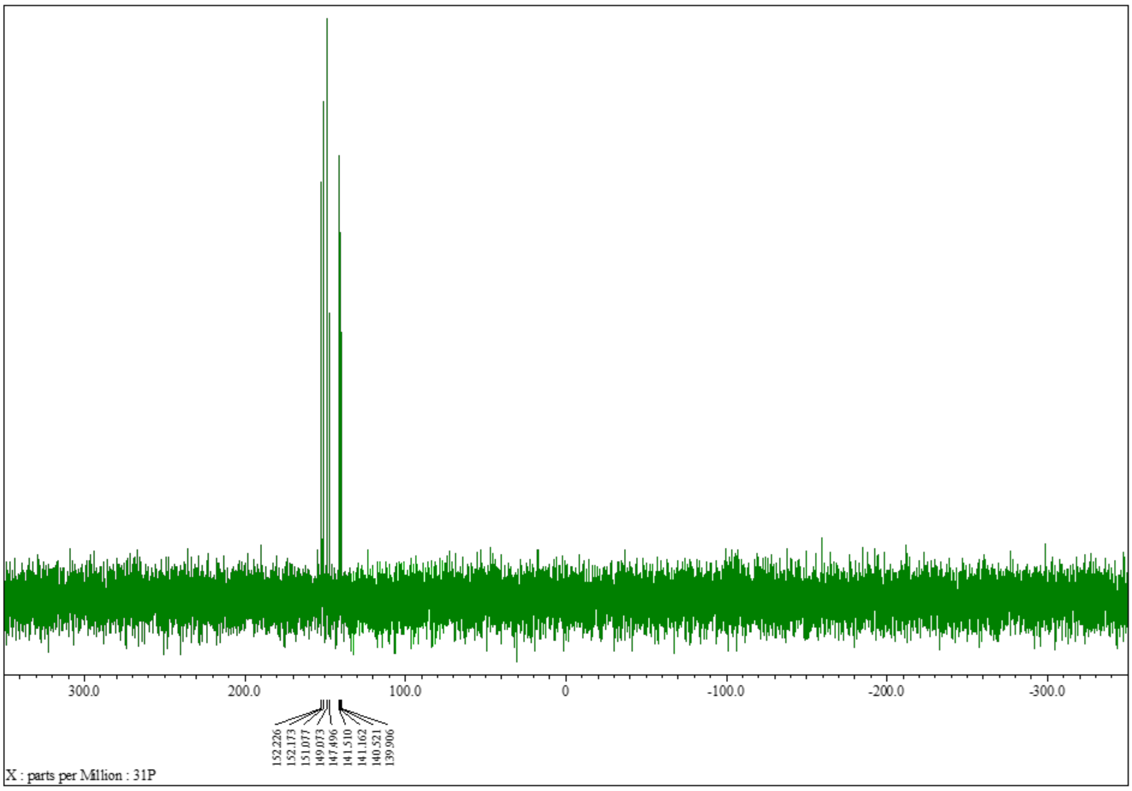
300.0 200.0 100.0 0 -100.0 -200.0 -300.0
152.226
152.173
151.077
149.073
147.496
141.510
141.162
140.521
139.906
X : parts per Million : 31P

Synthesis of DNA containing *o*-nitrobenzyl modifications

DNA containing *o*-nitrobenzyl modifications were synthesized by an automated DNA synthesizer (NRs-4A10R7NP, Nihon Techno Service). The synthesis was carried out at 0.2 μmol scale with DMTr-off using standard phosphoramidite chemistry. Since the coupling yield of amidites containing *o*-nitrobenzyl modification was low due to its steric hindrance, the coupling time was extended to 15 min. The phosphoramidite reagents for the natural nucleotide were as follows: deoxyadenosine (n-PAC) CED phosphoramidite (ANP-6661, ChemGenes), deoxyguanosine (n-tBPAC) CED phosphoramidite (ANP-5668, ChemGenes), deoxycytidine (n-acetyl) CED phosphoramidite (ANP-5560, ChemGenes), and thymidine CED phosphoramidite (ANP-5554, ChemGenes). The CPG supports for the natural nucleotide were as follows: deoxyadenosine (n-PAC) 3'-lcaa CPG 1000Å (N-P5101-10, ChemGenes), deoxyguanosine (n-PAC) 3'-lcaa CPG 1000Å (N-P-5103-10, ChemGenes), deoxycytidine (n-acetyl) 3'-lcaa CPG 1000Å (N-5212-10, ChemGenes), and thymidine 3'-lcaa CPG 1000Å (N-5104-10, ChemGenes). The 5'-phosphorylation was performed by 5'-Phosphate-ON-Reagent (CLP-1544, ChemGenes).

After synthesis, deprotection was performed by concentrated ammonia solution (1 mL) at room temperature for 16 hours. The CPG was then removed using a filter (Millex-LCR, 0.45 μm), and the solution was concentrated using a centrifugal evaporator. Subsequently, denaturing PAGE purification was performed to obtain the desired length DNA. Following that, reverse-phase HPLC purification was performed using YMC Hydrosphere C18 column (250 × 4.6 mm I.D., S-5 μm, 12 nm) with either HITACHI Chromaster system (pump: 5110, diode array detector: 5430) or HITACHI ELITE LaChrom system (pump: L-2130, diode array detector: L-2455). This separation was based on the presence or absence of the *o*-nitrobenzyl group, and the desired DNA was obtained. For this purpose, Solution A, consisting of 50 mM TEAA containing 5% ACN, and Solution B, consisting of ACN, were used as eluent. The flow rate was set at 1 mL/min, and a liner gradient was applied, increasing Solution B from 0% to 60% over 20 minutes. The column temperature was maintained at 50 °C. Each DNA was identified based on its molecular weight obtained by LCMS (1260 Infinity II Prime LC, Agilent, or ACQUITY UPLC H-Class PLUS, Waters).

Sequences and molecular weight of synthesized DNA are shown in Table S1.

ODN_23-25, 33, 36, 39, 40, 45-47, and 54, which do not contain *o*-nitrobenzyl modifications, were purchased from Eurofins and used without further purification or MS analysis.

Thermal stability evaluation of DNA containing *o*-nitrobenzyl modifications

Samples were prepared with 10 μM ODN in 1 × Thermo pol. Buffer (New England Biolabs) and subjected to a thermal cycle of [(95 °C, 1 min→50 °C, 30 sec→72 °C, 3 min)×30] using a T100 Thermal Cycler (Bio-Rad). They were then subjected to reverse-phase HPLC analysis on a YMC Hydrosphere C18 column (250 × 4.6 mm I.D., S-5 μm, 12 nm) using a HITACHI Chromaster (pump:

5110, diode array detector: 5430) or HITACHI ELITE LaChrom (pump: L-2130, diode array detector: L-2455). Solution A, consisting of 50 mM TEAA containing 5% ACN, and Solution B, consisting of ACN, were used as eluent. The flow rate was set at 1 mL/min, and a liner gradient was applied, increasing Solution B from 0% to 60% over 20 minutes. The column temperature was maintained at 50 °C.

Evaluation of removal of *o*-nitrobenzyl groups by photo irradiation

Samples were prepared with 10 μM ODN in water. and irradiated with 365 nm light at an intensity of 4 mW/cm^2 for 10 min using a MAX-350 compact xenon light source (Asahi Spectra). Samples were then subjected to reverse-phase HPLC analysis on a YMC Hydrosphere C18 column (250 × 4.6 mm I.D., S-5 μm, 12 nm) in the same method as in the experiment described above. Also, photo irradiated samples were subjected to LCMS to check Mass on ACQUITY UPLC BEH C18 Column (130 Å, 1.7 μm, 2.1 mm ×50 mm) using Agilent 1290 Infinity II and Agilent 6530 LC/Q-TOF. Solution A, consisting of 8.6 mM TEA / 100 mM HFIP, and Solution B, consisting of MeOH, were used as eluent. The flow rate was set at 0.3 mL/min, and a liner gradient was applied, increasing Solution B from 0% to 50% over 12 minutes. The column temperature was maintained at 60 °C. Obtained mass spectra were deconvoluted by Agilent MassHunter BioConfirm Software 12.0.

Primer extension assay using 6 kinds of DNA polymerases

Samples were prepared in a total volume of 20 μL with 1 μM ODN_25, 2 μM template, 0.2 mM dNTPs, 0.02 U/μL DNA polymerase in 1 × provided polymerase buffer. For DNA polymerase, KOD-Plus-Neo (Takara), PrimeSTAR HS (Takara), Pfu DNA polymerase (Promega), Phusion High Fidelity DNA polymerase (New England Biolabs), Q5 High Fidelity DNA polymerase (New England Biolabs), and Deep Vent DNA polymerase (New England Biolabs) were used and prepared the samples as per manufacturers instructions. Samples were reacted in a T100 Thermal Cycler at (95 °C, 1 min→50 °C, 30 sec→72 °C, 30 min). Afterwards, the reaction solution was mixed with an equal volume of 2×formamide loading solution (80% formamide, 10 mM EDTA (pH 8.0)), and then subjected to 20% denaturing PAGE analysis [20 (w/v)% acrylamide (ratio of acrylamide and bis-acrylamide was 19:1), 7.5 M urea, 1×Tris -borate-EDTA (TBE) buffer]. Each band was detected by fluorescence of FAM labeling of the primer using the ChemiDoc Touch MP imaging system (Bio-Rad). Stopping efficiency at the position of modification introduction was calculated using the ratio of band intensity to the positive control (ODN_23).

Primer extension assay under PCR condition

Samples were prepared in a total volume of 10 μL with 1 μM ODN_25, 2 μM template, 0.2 mM dNTPs, 0.02 U/μL Phusion High Fidelity in 1× Phusion High Fidelity buffer. ODN_4, 8-22 were used as a

template. They were subjected to a thermal cycle of [(95 °C, 1 min→55 °C, 15 sec→72 °C, 30 sec)×30] in T100 Thermal Cycler. The reaction solution was then mixed with an equal volume of 2×formamide loading solution, and then subjected to 20% denaturing PAGE analysis. Each band was detected by fluorescence of FAM labeling of the primer using the ChemDoc Touch MP imaging system. Stopping efficiency was calculated as the percentage of band intensity at each position, where the target was the position of modification introduction and the sum of five band intensities (±1, ±2 bases before and after the target) was set as 100%.

Confirmation of sticky ends formation using XhoI

PCR samples were prepared in a total volume of 800 μL with 0.1 μM ODN_35, 0.2 μM ODN_36, 0.25 ng/μL λ phage genome (New England Biolabs), 0.2 mM dNTPs, 0.04 U/μL Phusion High Fidelity in 1× Phusion High Fidelity buffer. The prepared mixture was divided into two equal parts, each with a volume of 400 μL. One was irradiated with 365 nm light at an intensity of 4 mW/cm^2 for 15 min using a MAX-350 compact xenon light source, designated as UV (+), and the other was UV (-). They were subjected to PCR with a thermal cycle of [(98 °C, 10 sec→55 °C, 30 sec→72 °C, 90 sec) × 30] in T100 Thermal Cycler. After the reaction, UV (-) was irradiated with 365 nm light at an intensity of 4 mW/cm^2 for 15 min. After alcohol precipitation, 0.8% agarose gel electrophoresis and gel extraction were performed using the Wizard SV Gel and PCR Clean-UP System (Promega) to obtain the target DNA fragments.

The obtained DNA fragments were subjected to XhoI treatment. Samples were prepared in 150 ng/μL UV (-/+) in 1× Cut Smart Buffer (New England BioLabs) in a total volume of 10 μL. To the prepared samples, 0.75 μL of XhoI (Takara, 10 U/μL) was added and incubated at 37 °C for 2 hours. The reaction solution was then analyzed by 16% native PAGE. Each band was stained with SYBR Green II and detected with a ChemiDoc Touch MP imaging system.

Ligation of 2 kbp and 3 kbp DNA fragments synthesized by stop primer

PCR samples were prepared with 0.1 μM stop primer, 0.2 μM primer, 0.25 ng/μL λ phage genome, 0.2 mM dNTPs, 0.04 U/μL Phusion High Fidelity in 1 × Phusion High Fidelity buffer in a total volume 200 μL in 1 × Phusion High Fidelity buffer. The primer combinations are as follows; 2 kbp fragment (50 nt sticky ends): ODN_33 and ODN_34, 3 kbp fragment (50 nt sticky ends): ODN_35 and ODN_36, 2 kbp fragment (20 nt sticky ends): ODN_33 and ODN_37, 3 kbp fragment (20 nt sticky ends): ODN_36 and ODN_38. They were subjected to PCR with a thermal cycle of [(98 °C, 10 sec→55 °C, 30 sec→72 °C, 90 sec) × 30] in T100 Thermal Cycler. After the reaction, samples were irradiated with 365 nm light at an intensity of 4 mW/cm^2 for 15 min using a MAX-350 compact xenon light source. After alcohol precipitation, 0.8% agarose gel electrophoresis and gel extraction were performed using the Wizard SV Gel and PCR Clean-UP System (Promega) to obtain the target DNA fragments.

The above obtained DNA fragments were used for ligation experiment. Samples were prepared with 10 nM 2 kbp DNA fragment, 15 nM 3 kbp DNA fragment, 0.2 mM dNTP in 1 × T4 DNA ligase buffer in a total volume of 30 μL. The DNA was then annealed at [80 °C, 5 min→-0.02 °C/sec to 15 °C] using T100 Thermal Cycler. This was divided into three equal parts, each with a volume of 10 μL. To one part, 0.2 μL of T4 DNA ligase (New England Biolabs, 2000 U/μL) was added, resulting in the Polymerase (-) Ligase (+) sample. To another part, 0.2 μL of both T4 DNA ligase and T4 DNA polymerase (New England Biolabs, 3 U/μL) was added, resulting in the Polymerase (+) Ligase (+) sample. The remaining part was left without any additions, resulting in the Polymerase (-) Ligase (-) sample. The samples were incubated at 16°C for 2 hours. After the reaction, 0.5 μL of Klenow Fragment (3'→5' exo-) (New England Biolabs, 50 U/μL) was added to each sample and incubated at 37 °C for 30 minutes and 68 °C for 10 minutes. The reaction solution was analyzed by 0.8% agarose gel. Each band was detected as fluorescence of FAM labeling of primer using a ChemiDoc Touch MP imaging system.

Ligation of 2 kbp and 3 kbp DNA fragments by Golden Gate assembly

PCR samples were prepared with 0.2 μM primer, 0.25 ng/μL λ phage genome, 0.2 mM dNTPs, 0.04 U/μL Phusion High Fidelity in 1 × Phusion High Fidelity buffer in a total volume of 50 μL. The primer combinations were as follows; 2 kbp fragment: ODN_33 and ODN_39, 3 kbp fragment: ODN_36 and ODN_40. They were subjected to PCR with a thermal cycle of [(98 °C, 10 sec→55 °C, 30 sec→72 °C, 90 sec) × 30] in T100 Thermal Cycler. After the reaction, the DNA was purified with the Wizard SV Gel and PCR Clean-Up System.

Golden Gate Assembly was performed using the obtained DNA fragments. Samples were prepared with 10 nM 2 kbp DNA fragment, 15 nM 3 kbp DNA fragment in 1 × T4 DNA ligase buffer in a total volume of 20 μL. They were divided into two equal parts, each with a volume of 10 μL. One was added 0.25 μL of T4 DNA ligase (2000 U/μL) and 0.25 μL of Bsa I HFv2 (New England Biolabs, 20 U/μL) as Ligase (+), and the other was left without any addition as Ligase (-). The samples were subjected to a thermal cycle of [(37 °C, 1 min→16 °C, 2 min) × 30]. After the reaction, the reaction solution was analyzed by 0.8% agarose gel. Each band was detected as fluorescence of FAM labeling of primer using a ChemiDoc Touch MP imaging system.

Plasmid construction with stop primer and confirmation by colony formation

PCR samples were prepared with 0.3 μM stop primer, 0.2 ng/μL plasmid, 0.2 mM dNTPs, 0.04 U/μL Phusion High Fidelity in 1 × Phusion High Fidelity buffer in a total volume of 400 μL. The primer combinations were as follows; fragment_LacZ: ODN_41 and ODN_42, fragment_AMPr: ODN_43 and ODN_44. The following plasmids were used as template; fragment_LacZ: pSV-β-Galactosidase Control Vector (Promega), fragment_AMPr: pUC19. The prepared samples were divided into two

equal parts, each with a volume of 200 μL. One was irradiated with 365 nm light at an intensity of 4 mW/cm^2 for 15 minutes using a MAX-350 compact xenon light source, designated as UV (+), and the other was UV (-). They were subjected to PCR with a thermal cycle of [(98 °C, 10 sec→55 °C, 30 sec→72 °C, 90 sec) × 35] in T100 Thermal Cycler. After the reaction, 20 μL of 10 × CutSmart Buffer and 2 μL of Dpn I (20 U/μL, New England BioLabs) were added and incubated at 37 °C for 2 hours. The UV (-) was then irradiated with 365 nm light at an intensity of 4 mW/cm^2 for 15 minutes. After alcohol precipitation, 0.8% agarose gel electrophoresis was performed, and gel extraction was performed using the Wizard SV Gel and PCR Clean-UP System to obtain the target DNA fragments.
The obtained DNA fragments were used for ligation experiments. Samples were prepared in a total volume of 10 μL with 18 nM fragment_LacZ, 2 nM fragment_AMPr, 0.2 mM dNTP in 1 × T4 DNA ligase buffer. The prepared samples were annealed using T100 Thermal Cycler at [80 °C, 5 min→-0.02 °C/sec to 15 °C]. Then, 0.2 μL of T4 DNA polymerase (3 U/μL) and 0.2 μL of T4 DNA ligase (2000 U/μL) were added and incubated at 16 °C for 2 hours. After the reaction, 3 μL of the reaction solution was added to 30 μL of ECOSTM Competent *E. coli* JM109 (NIPPON GENE) and kept on ice for 45 minutes, followed by heat shock at 42 °C for 2 minutes. 200 μL of LB medium was added to the sample, mixed, and precultured at 37 °C for 20 min. Centrifuged at 8000 rpm for 5 min, the supernatant was removed and resuspended in 50 μL of medium and cultured on agar medium (0.1 mg/mL ampicillin, 0.05 mg/mL X-gal, 0.4 mM IPTG) for 24 h.

Sequence analysis of constructed plasmid

Blue colonies were placed in 3 mL of LB medium with ampicillin (100 μg/mL) and shaken at 180 rpm for 15 h at 37°C. Plasmids were extracted using the Wizard® Plus SV Minipreps DNA Purification System.
Samples used for sequence analysis were prepared with 0.5 μM primer and 40 ng/μL plasmid in a total volume of 10 μL. The primers used were 5'-CGTTATCCCCTGATTCTGTGGA-3' and 5'-CCACCTGACGTCTAAGAAACCA-3'. The sequencing mixtures were analyzed by 3500 Genetic Analyzer (Applied Biosystems) at Nagoya University Center for Gene Research.

Hybridization of 19 kbp DNA fragments

PCR samples were prepared with 0.1 μM stop primer, 0.2 μM primer, 0.25 ng/μL λ phage genome, 0.2 mM dNTPs, 0.04 U/μL Phusion High Fidelity in 1 × Phusion High Fidelity buffer in a total volume of 200 μL. The primer combinations were as follows; 19 kbp former: ODN_34 and ODN_45, 19 kbp latter: ODN_35 and ODN_46. The prepared samples were subjected to PCR with a thermal cycle of [(98 °C, 10 sec→55 °C, 20 sec→72 °C, 9.5 min) × 30] in T100 Thermal Cycler. After the reaction, the samples were irradiated with 365 nm light at an intensity of 4 mW/cm^2 for 15 min using a MAX-350 compact xenon light source. After alcohol precipitation, 0.8% agarose gel electrophoresis and gel

extraction were performed using the Wizard SV Gel and PCR Clean-UP System to obtain the target DNA fragments.

Hybridization experiments were performed using the obtained DNA fragments. Samples were prepared with 1 nM 19 kbp former fragment, 1 nM 19 kbp latter fragment, 0.2 mM dNTP in 1 × T4 DNA ligase buffer in a total volume of 10 μL. The prepared samples were annealed using T100 Thermal Cycler at [80 °C, 5 min→-0.02 °C/sec to 15 °C]. The reaction solution was analyzed by 0.75% agarose gel using a pulsed-field electrophoresis system (Pippin Pulse (sage science)) at protocol 2 for 15 h. λ DNA-Mono Cut Mix (New England Biolabs) was used as the ladder marker. Each band was stained with ethidium bromide using the ChemiDoc Touch MP imaging system.

Design of multiple DNA fragments and stop primers

We designed four DNA fragments (fragment_1-4) and six stop primers (ODN_48-53) to evaluate the feasibility of constructing the lambda phage genome from multiple DNA fragments. ODN_48 and ODN_49 possess complementary annealing regions that bridge fragment_1 and _2. Similarly, ODN_50 and ODN_51 contain complementary annealing regions that connect fragment_2 and _3, while ODN_52 and ODN_53 have complementary annealing regions linking fragment_3 and _4. The position and sequence of the stop primers were determined such that the length of DNA fragments was similar, and binding energy between annealing regions was as low as possible. In addition, binding specificity between annealing regions were also considered to avoid the possibility of mis-ligation in simultaneous ligation of multiple DNA fragments.

First, we extracted candidate primer regions from both strands of the lambda phage genome with the MFEprimer program. Then, we selected approximately 26,500 pairs of candidate primers having complementary annealing regions. Subsequently, we explored a suitable set of primer pairs with the help of NSGA-II, a multi-objective genetic algorithm. Specifically, we used binding energy (minimum binding energy for correct annealing regions) and binding specificity (minimum difference in binding energy between correct and incorrect annealing regions) for criteria of exploring better solutions. During the exploration, we set a maximum constraint of 16 kb for the length of each DNA fragment. The binding energy between annealing regions was calculated using the hybrid-min program from the UNAfold package.

Simultaneous ligation of multiple long DNA fragments

PCR samples were prepared with 0.3 μM primer, 0.25 ng/μL λ phage genome, 0.2 mM dNTPs, 0.04 U/μL Phusion High Fidelity in 1 × Phusion High Fidelity buffer in a total volume of 200 μL. The primer combinations were as follows; fragment 1: ODN_47 and ODN_48, fragment 2: ODN_49 and ODN_50, fragment 3: ODN_51 and ODN_52, fragment 4: ODN_53 and ODN_54. The prepared samples were subjected to PCR with a thermal cycle of [(98 °C, 10 sec→55 °C, 20 sec→72 °C, 9.5

min) × 30] for fragment 1, 2, and 4 and [(98 °C, 10 sec→50 °C, 20 sec→72 °C, 9.5 min) ×30] for fragment 3 in T100 Thermal Cycler. After the reaction, the samples were irradiated with 365 nm light at an intensity of 4 mW/cm^2 for 15 min using a MAX-350 compact xenon light source. After alcohol precipitation, 0.8% agarose gel electrophoresis and gel extraction were performed using the Wizard SV Gel and PCR Clean-UP System to obtain the target DNA fragments.

Hybridization experiments were performed using the obtained DNA fragments. Samples were prepared with 1 nM DNA fragment 1-4, 0.2 mM dNTP in 1 × T4 DNA ligase buffer in a total volume of 10 μL. The prepared samples were left at room temperature or 50 °C for 7 days. Reaction solution was analyzed by 0.75% agarose gel using a pulsed-field electrophoresis system (Pippin Pulse) at protocol 2 for 15 h. Each band was stained with ethidium bromide using the ChemiDoc Touch MP imaging system.

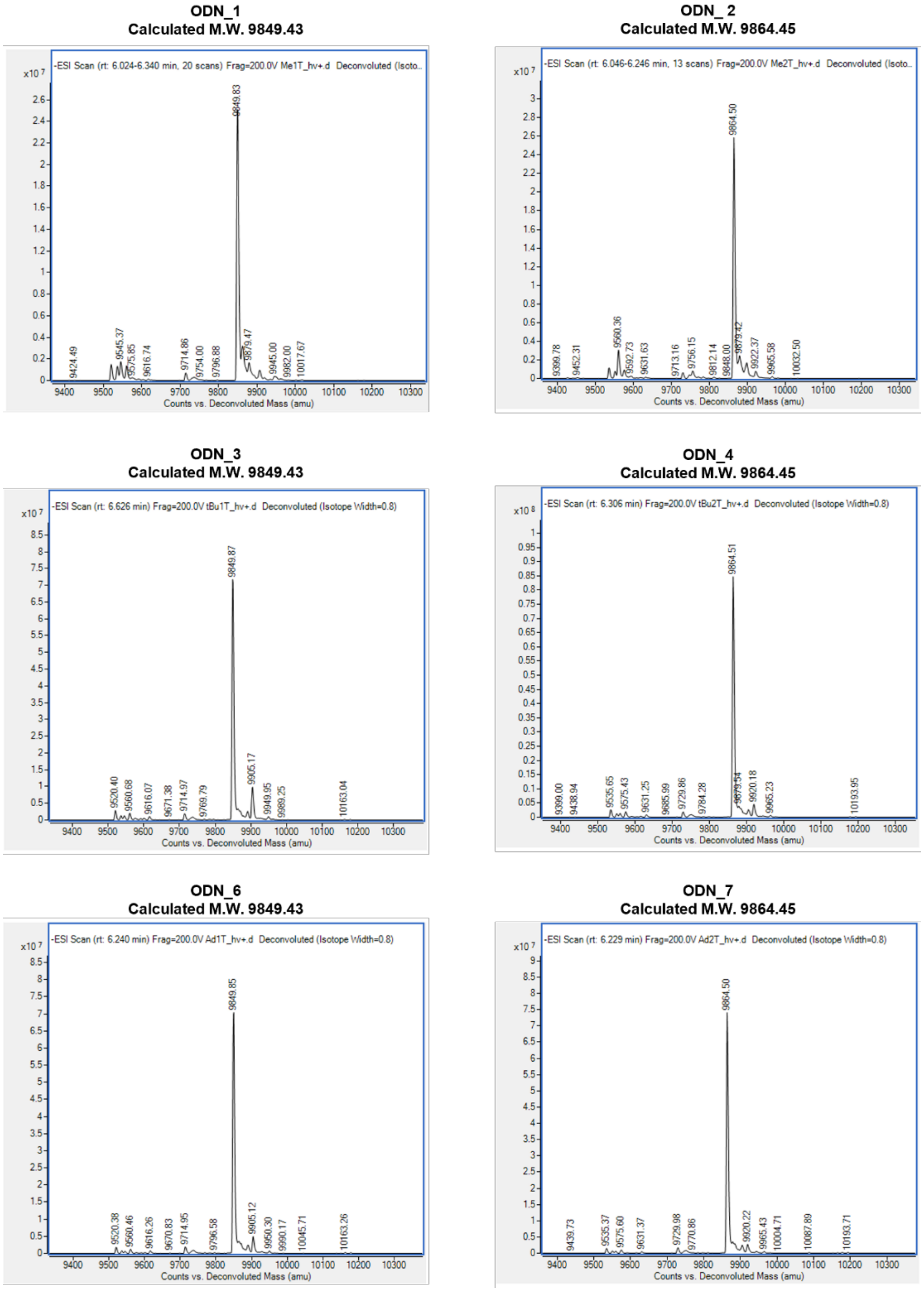

Fig. S1 Mass spectra of photo irradiated DNA oligo. The ESI-MS analysis of photo irradiated ODN_1-4, 6, and 7 were performed by LCMS (Agilent). The obtained mass spectra were deconvoluted by Agilent MassHunter BioConfirm Software 12.0.

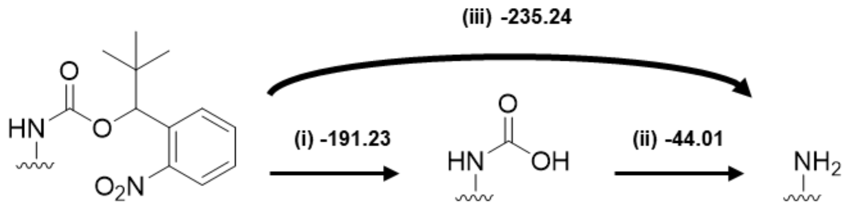

Sample	Target M.W.	Observed M.W.	Δ M.W.	Degradation
ODN_26	10093.68	9858.40	-235.28	(iii)
		9901.20	-192.48	(i)
ODN_27	10352.95	9882.40	-470.55	(iii) × 2
		9925.40	-427.55	(i) + (iii)
		9968.20	-384.75	(i) × 2
ODN_28	10284.91	10049.60	-235.31	(iii)
		10092.50	-192.41	(i)
ODN_29	10735.41	10265.20	-470.21	(iii) × 2
		10308.10	-427.31	(i) + (iii)
		10351.00	-384.41	(i) × 2
ODN_30	10751.40	10558.74	-192.66	(i)
ODN_31	10727.38	10493.11	-234.27	(iii)
ODN_32	10727.38	10493.08	-234.30	(iii)
		10536.11	-191.27	(i)

Fig. S2 Degradation of *o*-nitrobenzyl groups by ammonia water. During deprotection after DNA synthesis, *o*-nitrobenzyl group was removed by ammonia water. This is the cause of low stopping efficiency of chain elongation in the case of using *o*-nitrobenzyl group at base part.

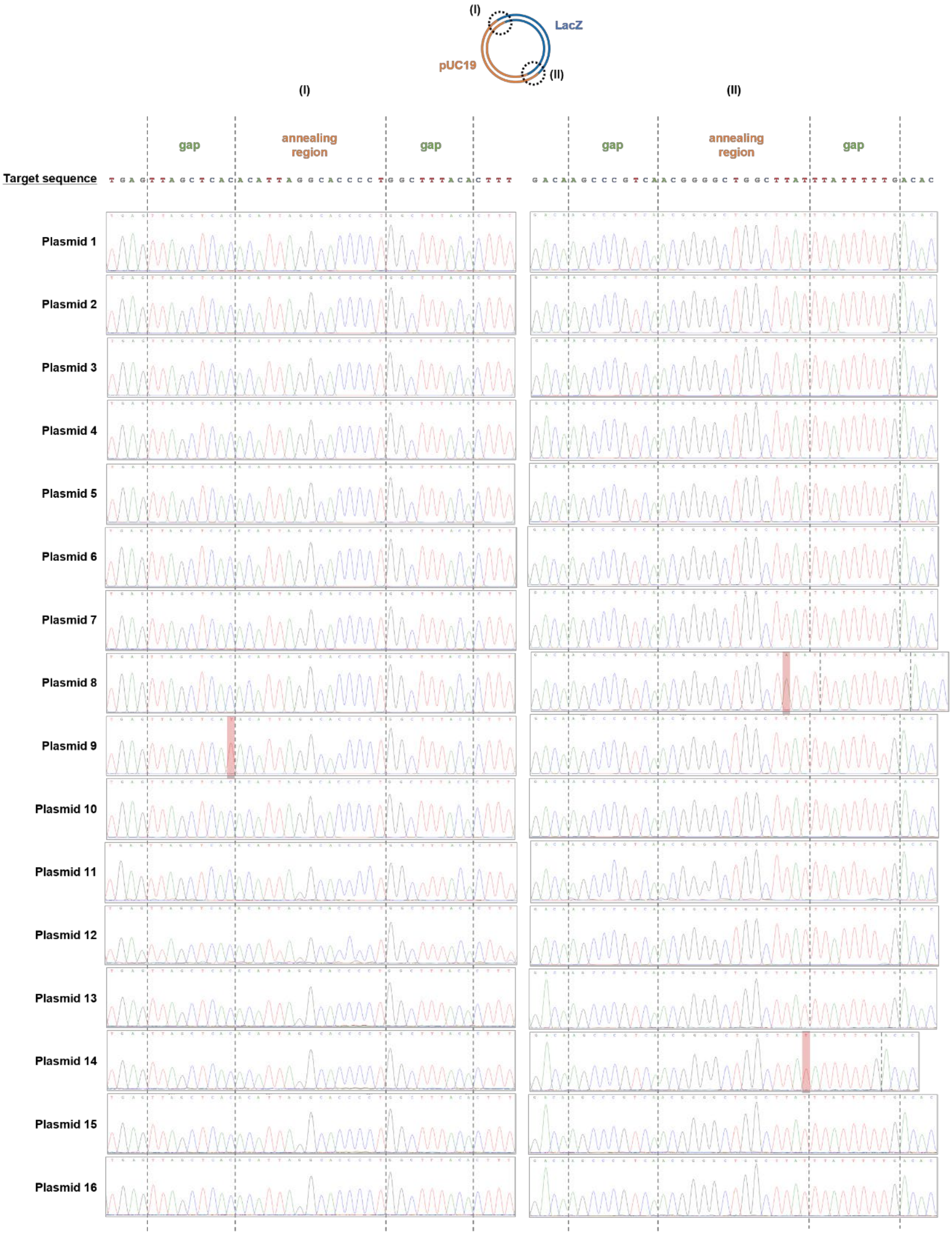

Fig. S3 Sanger sequencing analysis of constructed plasmids. 16 blue colonies of Figure 5 were collected, and plasmids were extracted. For each plasmid, the sequences of the ligation regions were analyzed by Sanger sequencing. Areas where errors were seen are indicated in red. Plasmid 8 had an A insert in the annealing region. Plasmid 9 had a C to T mutation in the gap region. Plasmid 14 skipped two bases in the gap region.
